# Supplementary material for: Multiplex base editing of BCL11A regulatory elements to treat sickle cell disease
Source: Cell Rep Med. 2025 Sep 26;6(10):102376. doi: 10.1016/j.xcrm.2025.102376 (PMC12629801; doi:10.1016/j.xcrm.2025.102376)
Supplement: Document S1. Figures S1–S8 and Table S1 [file mmc1.pdf]

Cell Reports Medicine, Volume *6*

Supplemental information

Multiplex base editing of *BCL11A* regulatory elements to treat sickle cell disease

Letizia Fontana, Pierre Martinucci, Simone Amistadi, Tristan Felix, Margaux Mombled, Alexandra Tachtsidi, Guillaume Corre, Anne Chalumeau, Giulia Hardouin, Jeanne Martin, Oriana Romano, Mario Amendola, Panagiotis Antoniou, and Annarita Miccio

SUPPLEMENTARY FIGURE TITLES AND LEGENDS

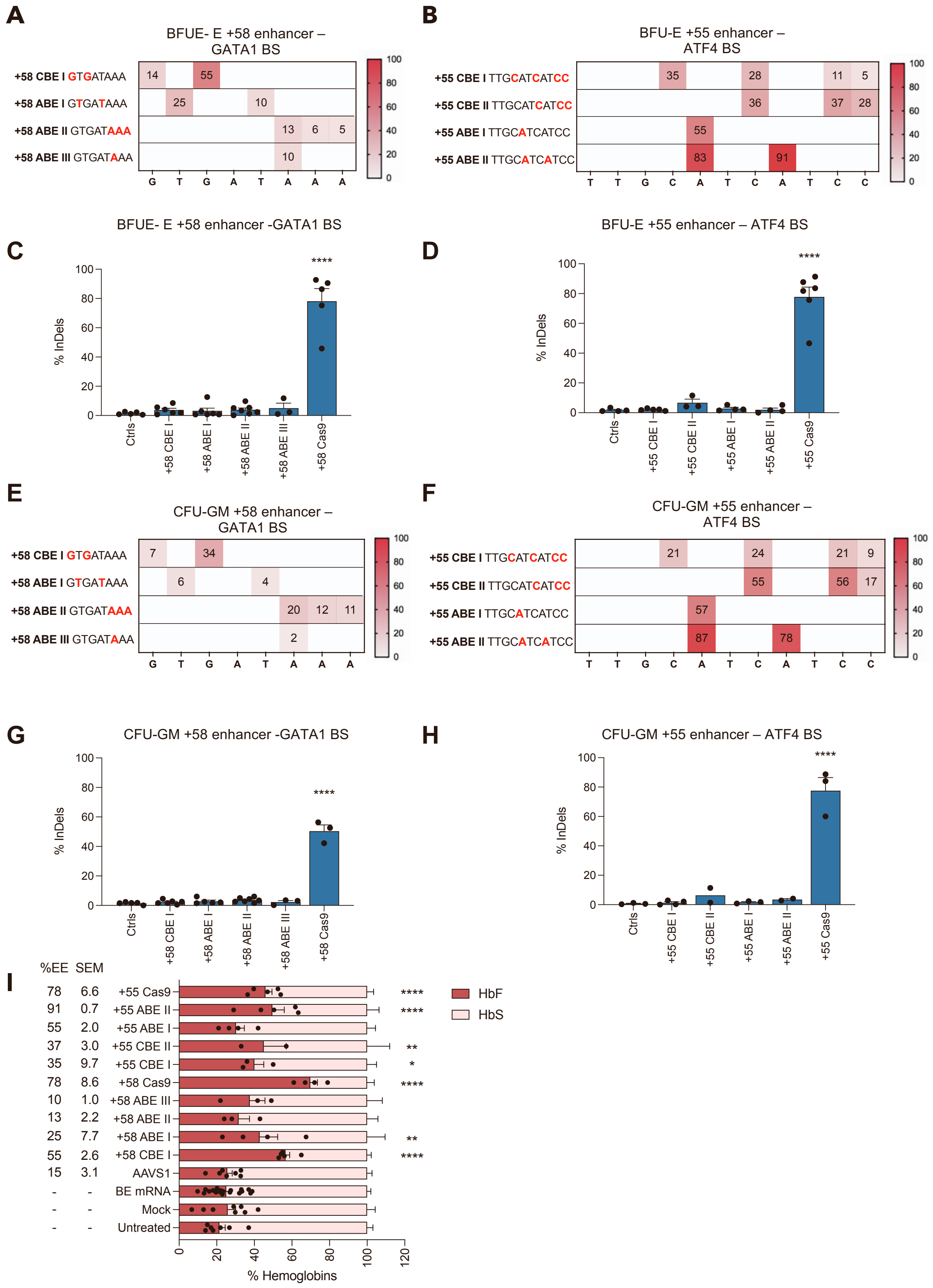

Figure S1. Base editing of the erythroid-specific *BCL11A* enhancers in SCD HPSC-derived BFU-E and CFU-GM colonies.

A-B. C-G to T-A or A-T to G-C base-editing efficiency calculated by the EditR software in samples subjected to Sanger sequencing in pools of BFU-E colonies derived from SCD HSPCs edited in the +58-kb (**A**) or +55-kb (**B**) regions. Data are expressed as mean (n = 2 to 4 biologically independent experiments, 2 to 5 donors).

C-D. Frequency of InDels, measured by TIDE analysis, in samples subjected to Sanger sequencing in pools of BFU-E colonies derived from SCD HSPCs edited in the +58-kb (**C**) or +55-kb (**D**) regions. Data are expressed as mean ± SEM (n = 2 to 4 biologically independent experiments, 2 to 5 donors). ****P ≤ 0.0001 (One-way ANOVA. Comparison of controls vs edited samples)

E-F. C-G to T-A or A-T to G-C base-editing efficiency calculated by the EditR software in samples subjected to Sanger sequencing in pools of CFU-GM colonies derived from SCD HSPCs edited in the +58-kb (**E**) or +55-kb (**F**) regions. Data are expressed as mean (n = 2 to 4 biologically independent experiments, 2 to 5 donors).

G-H. Frequency of InDels, measured by TIDE analysis, in samples subjected to Sanger sequencing in pools of CFU-GM colonies derived from SCD HSPCs edited in the +58-kb (**G**) or +55-kb (**H**) regions. Data are expressed as mean ± SEM (n = 2 to 4 biologically independent experiments, 2 to 5 donors). ****P ≤ 0.0001 (One-way ANOVA. Comparison of controls vs edited samples)

I. Analysis of HbF and HbS by cation-exchange HPLC in pools of BFU-E colonies. We calculated the percentage of each Hb type over the total Hb tetramers. Data are expressed as mean ± SEM (n = 3 to 4 biologically independent experiments, 3 to 4 donors). *P ≤ 0.05; **P ≤ 0.01; ****P ≤ 0.0001 (two-way ANOVA with Dunnett correction for multiple comparisons. Statistical significance between mock and edited samples is depicted in the graph).

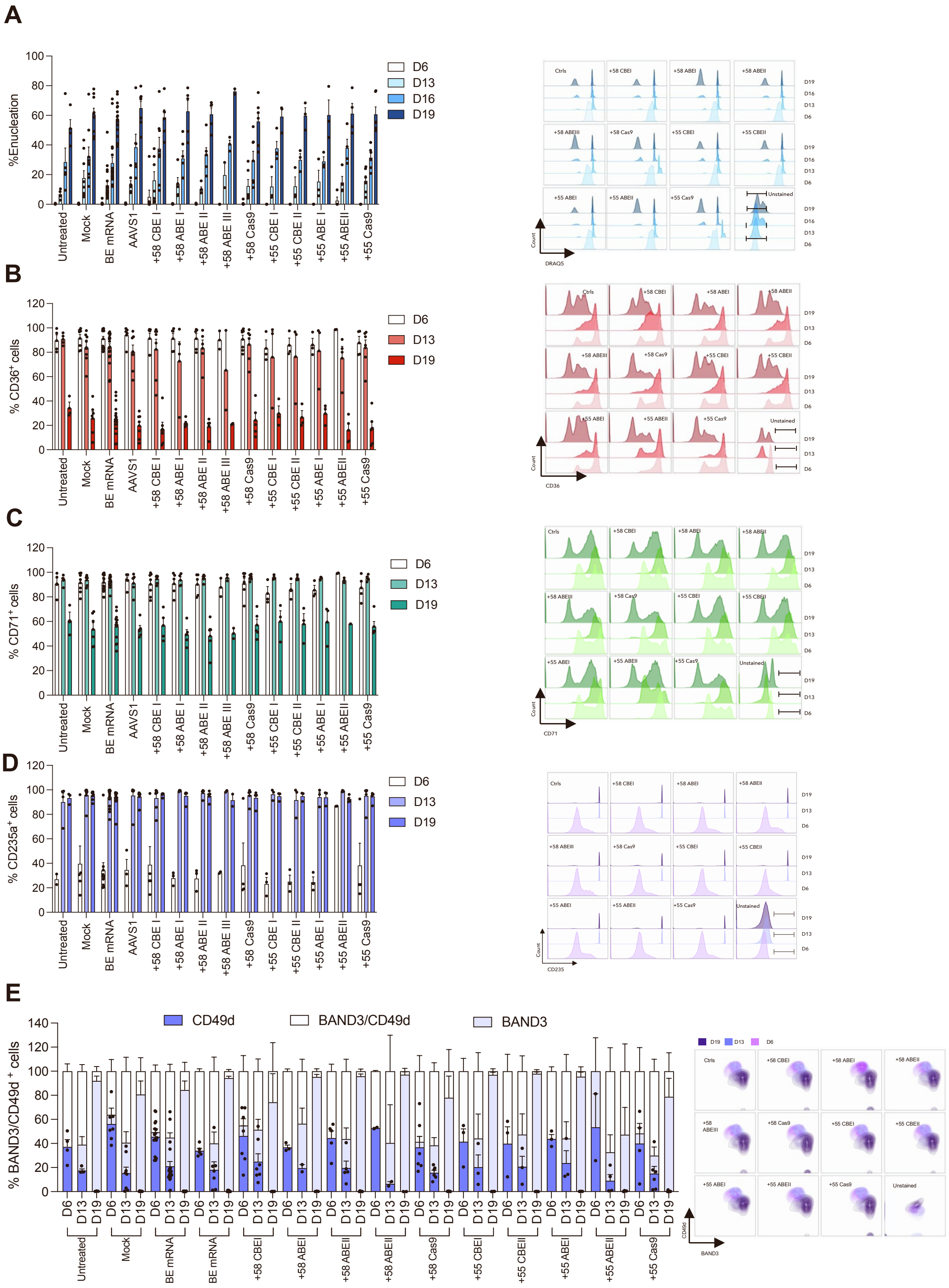

A
%Enucleation
D6
D13
D16
D19
Untreated
Mock
BE mRNA
AAVS1
+58 CBE I
+58 ABE I
+58 ABE II
+58 ABE III
+58 Cas9
+55 CBE I
+55 CBE II
+55 ABE I
+55 ABEII
+55 Cas9
Count
DRAQ5
B
% CD36+ cells
D6
D13
D19
CD36
C
% CD71+ cells
D6
D13
D19
CD71
D
% CD235a+ cells
D6
D13
D19
CD235
E
CD49d
BAND3/CD49d
BAND3
% BAND3/CD49d + cells
Untreated
Mock
BE mRNA
BE mRNA
+58 CBEI
+58 ABEI
+58 ABEII
+58 ABEII
+58 Cas9
+55 CBEI
+55 CBEII
+55 ABEI
+55 ABEII
+55 Cas9
CD49d
BAND3
Unstained

Figure S2. Erythroid differentiation of SCD HSPCs upon base editing of the erythroid-specific *BCL11A* enhancers.

A. Frequency of enucleated cells at day 6, 13, 16, and 19 of erythroid differentiation, as measured by flow cytometry analysis of DRAQ5 nuclear staining in control and edited samples. Data are expressed as mean ± SEM (n = 2 to 6 biologically independent experiments, 2 to 5 donors). Representative flow cytometry histograms showing the DRAQ5$^-$ cell population for control and edited samples are reported.

B-D. Frequency of CD36$^+$ (**B**), CD71$^+$ (**C**), and CD235a$^+$ (**D**) cells at day 6, 13, and 19 of erythroid differentiation, as measured by flow cytometry analysis of CD36, CD71, and CD235a erythroid markers. Data are expressed as mean ± SEM (n = 2 to 6 biologically independent experiments, 2 to 5 donors). Representative flow cytometry histograms showing the CD36$^+$ (**B**), CD71$^+$ (**C**), and CD235a$^+$ (**D**) cell population for control and edited samples are reported.

E. Frequency of CD49d$^+$, BAND3$^+$ and CD49d$^+$/BAND3$^+$ in 7AAD$^-$/CD235a$^+$ cells at day 6, 13, and 19 of erythroid differentiation, as measured by flow cytometry analysis of CD49d and BAND3 erythroid markers. Data are expressed as mean ± SEM (n = 2 to 6 biologically independent experiments, 2 to 5 donors). Representative flow cytometry contour plots showing the CD49d$^+$, BAND3$^{+,}$ and CD49d$^+$/BAND3$^+$ cell population for control and edited samples are reported.

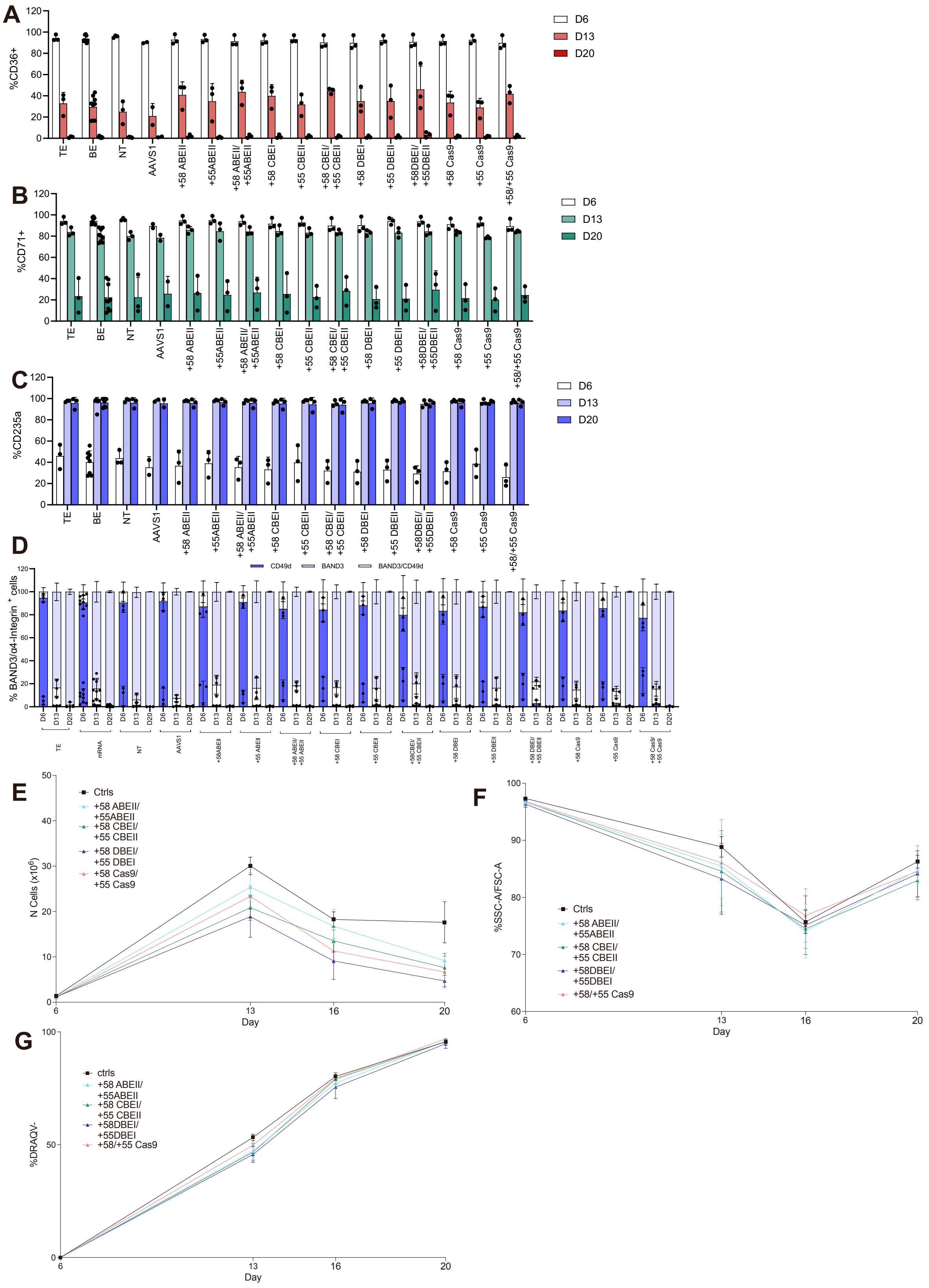
A
B
C
D
E
F
G
D6
D13
D20
%CD36+
%CD71+
%CD235a
% BAND3/α4-Integrin + cells
CD49d
BAND3
BAND3/CD49d
TE
BE
NT
AAVS1
+58 ABEII
+55ABEII
+58 ABEII/ +55ABEII
+58 CBEI
+55 CBEII
+58 CBEI/ +55 CBEII
+58 DBEI
+55 DBEII
+58DBEI/ +55DBEII
+58 Cas9
+55 Cas9
+58/+55 Cas9
mRNA
N Cells (x10⁶)
%SSC-A/FSC-A
%DRAQV-
Day
Ctrls
+58 ABEII/ +55ABEII
+58 CBEI/ +55 CBEII
+58 DBEI/ +55 DBEI
+58 Cas9/ +55 Cas9
+58DBEI/ +55DBEI
+58/+55 Cas9
ctrls

Figure S3. Erythroid differentiation and cell growth of SCD HSPCs after single- and multiplex base editing of the erythroid-specific *BCL11A* enhancers.

A-C. Frequency of $CD36^+$ (**A**), $CD71^+$ (**B**), and $CD235a^+$ (**C**) cells at day 6, 13, and 20 of erythroid differentiation, as measured by flow cytometry analysis of CD36, CD71, and CD235a erythroid markers. Data are expressed as mean ± SEM (n = 3 biologically independent experiments, 3 donors).

D. Frequency of $CD49d^+$, $BAND3^+$ and $CD49d^+/BAND3^+$ in $7AAD^-/CD235a^+$ cells at day 6, 13, and 19 of erythroid differentiation, as measured by flow cytometry analysis of CD49d and BAND3 erythroid markers. Data are expressed as mean ± SEM (n = 3 biologically independent experiments, 3 donors).

E. Analysis of the cell growth over time. The number of cells was measured at multiple time points to assess growth dynamics (day 6,13,16, and 20 of erythroid differentiation). Data are expressed as mean ± SEM (n = 3 biologically independent experiments, 3 donors).

F. Analysis of cell viability over time. We reported the percentage of live cells over the total live and dead/apoptotic cells identified based on size and granularity. Viability was assessed at days 6, 13, 16, and 20 of erythroid differentiation. Data are presented as mean ± SEM (n = 3 biologically independent experiments, from 3 donors).

G, Frequency of enucleated cells as measured by flow cytometry analysis of DRAQ5 nuclear staining in control and edited samples was reported at day 6, 13, 16, and 20 of erythroid differentiation. Data are expressed as mean ± SEM (n = 3 biologically independent experiments, 3 donors).

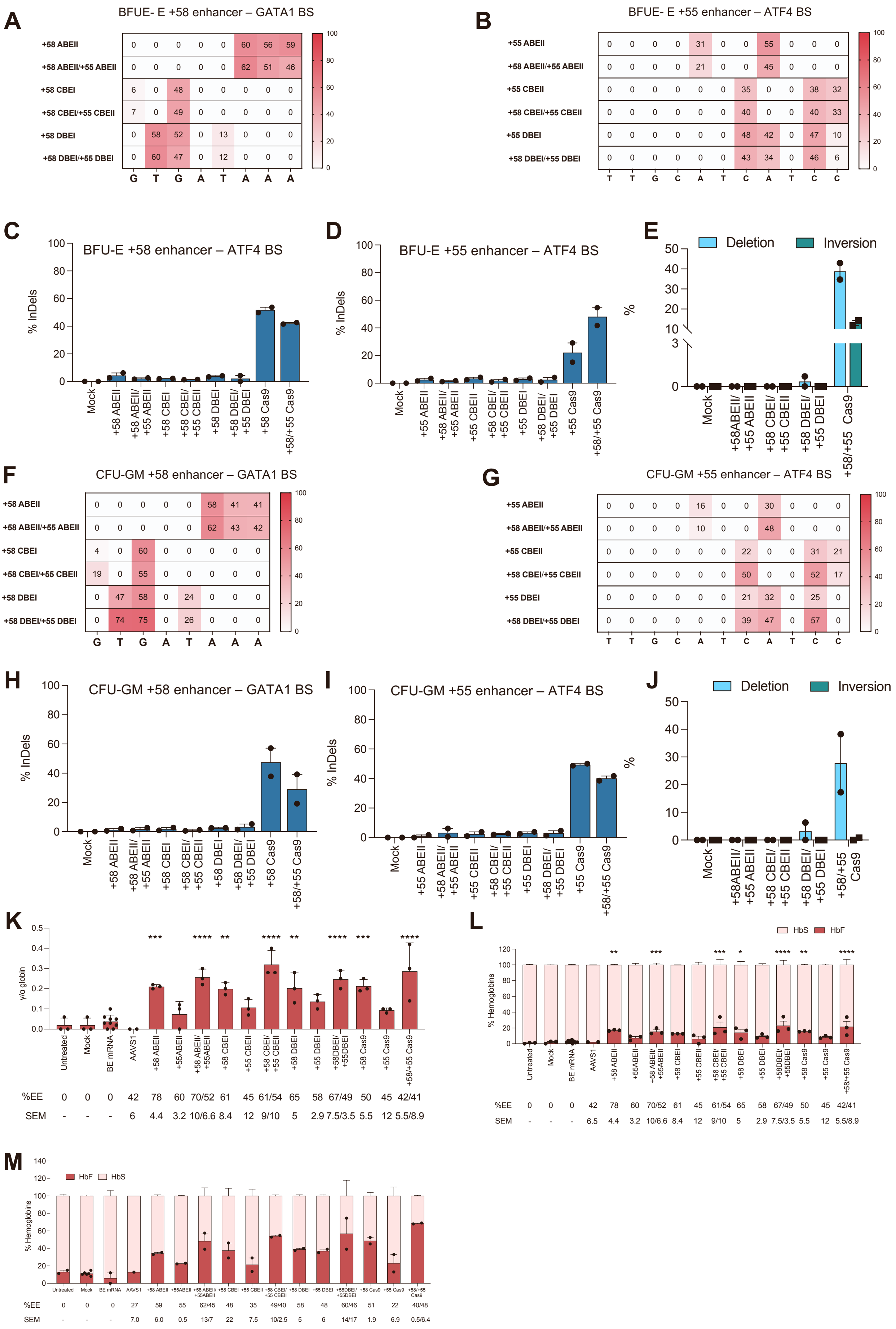
A
BFUE- E +58 enhancer – GATA1 BS
+58 ABEII
+58 ABEII/+55 ABEII
+58 CBEI
+58 CBEI/+55 CBEII
+58 DBEI
+58 DBEI/+55 DBEI
G T G A T A A A
B
BFUE- E +55 enhancer – ATF4 BS
+55 ABEII
+58 ABEII/+55 ABEII
+55 CBEII
+58 CBEI/+55 CBEII
+55 DBEI
+58 DBEI/+55 DBEI
T T G C A T C A T C C
C
BFU-E +58 enhancer – ATF4 BS
% InDels
D
BFU-E +55 enhancer – ATF4 BS
% InDels
E
Deletion
Inversion
%
F
CFU-GM +58 enhancer – GATA1 BS
G
CFU-GM +55 enhancer – ATF4 BS
H
CFU-GM +58 enhancer – GATA1 BS
% InDels
I
CFU-GM +55 enhancer – ATF4 BS
% InDels
J
Deletion
Inversion
%
K
γ/α globin
%EE 0 0 0 42 78 60 70/52 61 45 61/54 65 58 67/49 50 45 42/41
SEM - - - 6 4.4 3.2 10/6.6 8.4 12 9/10 5 2.9 7.5/3.5 5.5 12 5.5/8.9
L
HbS
HbF
% Hemoglobins
%EE 0 0 0 42 78 60 70/52 61 45 61/54 65 58 67/49 50 45 42/41
SEM - - - 6.5 4.4 3.2 10/6.6 8.4 12 9/10 5 2.9 7.5/3.5 5.5 12 5.5/8.9
M
HbF
HbS
% Hemoglobins
Untreated Mock BE mRNA AAVS1 +58 ABEII +55ABEII +58 ABEII/+55ABEII +58 CBEI +55 CBEII +58 CBEI/+55 CBEII +58 DBEI +55 DBEI +58DBEI/+55DBEI +58 Cas9 +55 Cas9 +58/+55 Cas9
%EE 0 0 0 27 59 55 62/45 48 35 49/40 58 48 60/46 51 22 40/48
SEM - - - 7.0 6.0 0.5 13/7 22 7.5 10/2.5 5 6 14/17 1.9 6.9 0.5/6.4

Figure S4. **Multiplex base editing of the erythroid-specific *BCL11A* enhancers.**
A-B. C-G to T-A or/and A-T to G-C base-editing efficiency in the +58-kb (**A**) or +55-kb (**B**) regions calculated using EditR in samples subjected to Sanger sequencing in BFU-E colonies derived from SCD HSPCs edited at either the +58-kb or the +55-kb region, or simultaneously edited at both the +58-kb and the +55-kb regions. Data are expressed as mean (n = 2 biologically independent experiments, 2 donors).
C-D. Frequency of InDels in the +58-kb (**C**) or +55-kb (**D**) regions measured by TIDE analysis, in samples subjected to Sanger sequencing in pools of BFU-E colonies differentiated from SCD HSPCs edited at either the +58-kb or the +55-kb regions, or simultaneously edited at both the +58-kb and the +55-kb regions. Data are expressed as mean ± SEM (n = 2 biologically independent experiments, 2 donors).
E. Frequency of the 3.2-kb deletion/inversion, measured by ddPCR, in BFU-E colonies simultaneously edited at the +58-kb and +55-kb regions. Data are expressed as mean ± SEM (n = 2 biologically independent experiments, 2 donors).
F-G. C-G to T-A or/and A-T to G-C base-editing efficiency in the +58-kb (**F**) or +55-kb (**G**) regions calculated using EditR in samples subjected to Sanger sequencing in CFU-GM colonies derived from SCD HSPCs edited at either the +58-kb or the +55-kb region, or simultaneously edited at both the +58-kb and the +55-kb regions. Data are expressed as mean (n = 2 biologically independent experiments, 2 donors).
H-I. Frequency of InDels in the +58-kb (**H**) or +55-kb (**I**) regions measured by TIDE analysis, in samples subjected to Sanger sequencing in pools of CFU-GM colonies, differentiated from SCD HSPCs edited at either the +58-kb or the +55-kb regions, or simultaneously edited at both the +58-kb and the +55-kb regions. Data are expressed as mean ± SEM (n = 2 biologically independent experiments, 2 donors).
J. Frequency of the 3.2-kb deletion/inversion, measured by ddPCR, in CFU-GM colonies simultaneously edited at the +58-kb and +55-kb regions. Data are expressed as mean ± SEM (n = 2 biologically independent experiments, 2 donors). *P ≤ 0.05; (two-way ANOVA with Sidak correction for multiple comparisons. Comparison of mock vs edited samples).
K. Expression of γ ($^{G}\gamma$ + $^{A}\gamma$)-globin chains measured by RP-HPLC in RBCs derived from SCD HSPCs. γ-globin expression was normalized to α-globin. The EE ± SEM is indicated for each sample in the lower part of the panel. Data are expressed as mean ± SEM (n=3 biologically independent experiments, 3 donors). **P ≤ 0.01; ***P ≤ 0.001; ****P ≤ 0.0001 (one-way ANOVA with Dunnett correction for multiple comparisons. Statistical significance between mock and edited samples is depicted in the graph).
L. Analysis of HbF and HbS by cation-exchange HPLC in RBCs derived from edited SCD HSPCs. We calculated the percentage of each Hb type over the total Hb tetramers. The EE ± SEM is indicated for each sample in the lower part of the panel. Data are expressed as mean ± SEM (n = 3 biologically independent experiments, 3 donors). *P ≤ 0.05; **P ≤ 0.01; ***P ≤ 0.001; ****P ≤ 0.0001 (two-way ANOVA with Dunnett correction for multiple comparisons. Statistical significance between mock and edited samples is depicted in the graph).
M. Analysis of HbF and HbS by cation-exchange HPLC in pools of BFU-E colonies derived from edited SCD HSPCs. We calculated the percentage of each Hb type over the total Hb tetramers. Data are expressed as mean ± SEM (n = 2 biologically independent experiments, 2 donors).

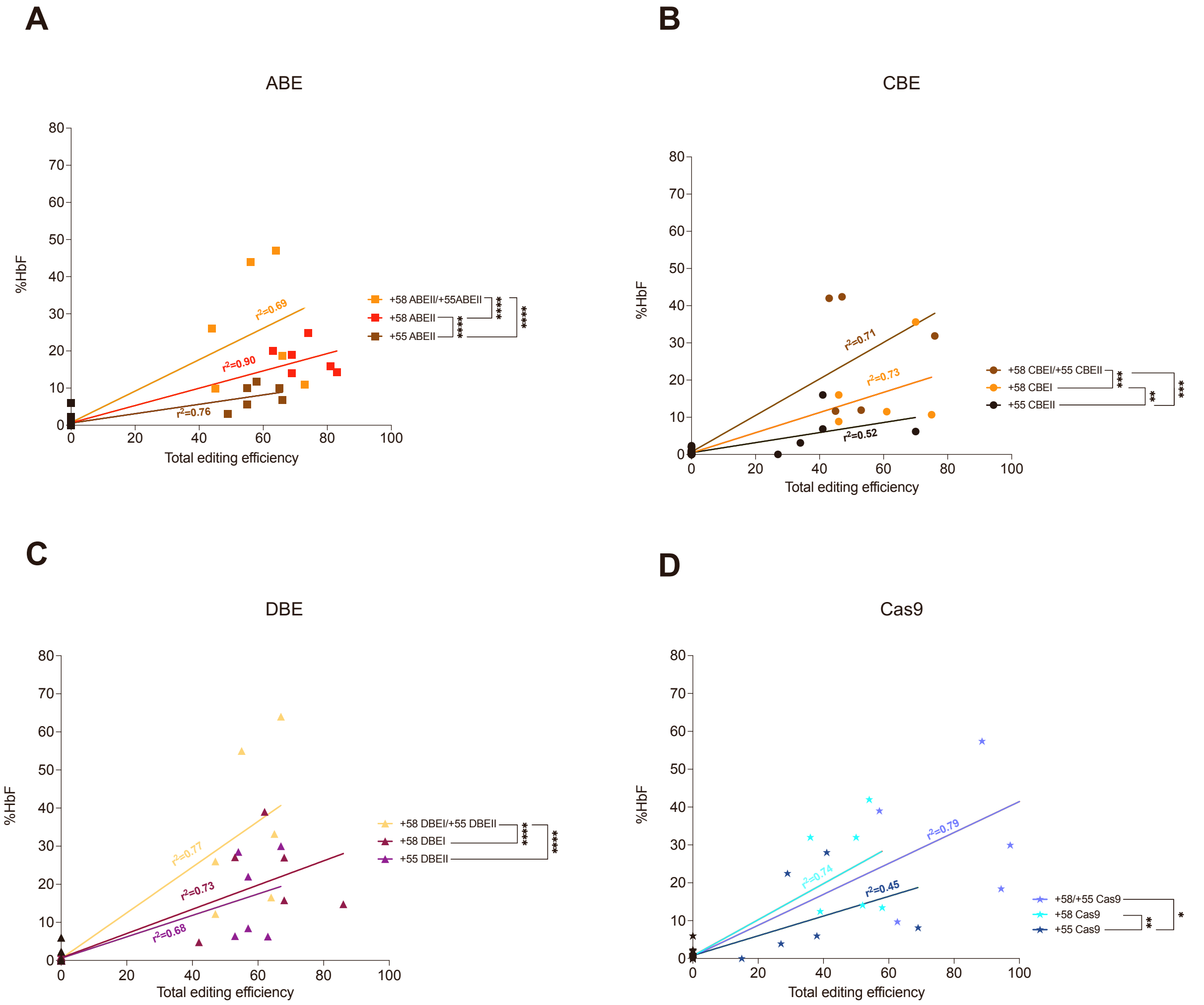

Figure S5. Correlation between HbF expression and editing efficiency obtained with ABE, CBE, DBE, and Cas9 nuclease.

Correlation between HbF expression and editing efficiency in erythroid cells derived from SCD HSPCs edited with either ABE (**A**), CBE (**B**), DBE (**C**), or Cas9-nuclease (**D**) (erythroblasts and pools of BFU-E; n = 3 biological independent experiments, 3 donors). HbF expression was measured by cation-exchange HPLC and calculated over the total Hb tetramers. Total editing efficiency was calculated by adding the base editing and Cas9 editing efficiency (determined by Sanger sequencing at the individual sites) to the frequency of the 3.2-kb deletion/inversion detected by ddPCR. Base-editing efficiency was calculated using EditR, and Cas9-editing efficiency (InDels) was calculated using TIDE in samples subjected to Sanger sequencing. *$P \leq 0.05$; **$P \leq 0.01$; ***$P \leq 0.001$; ****$P \leq 0.0001$ (Multiple t test).

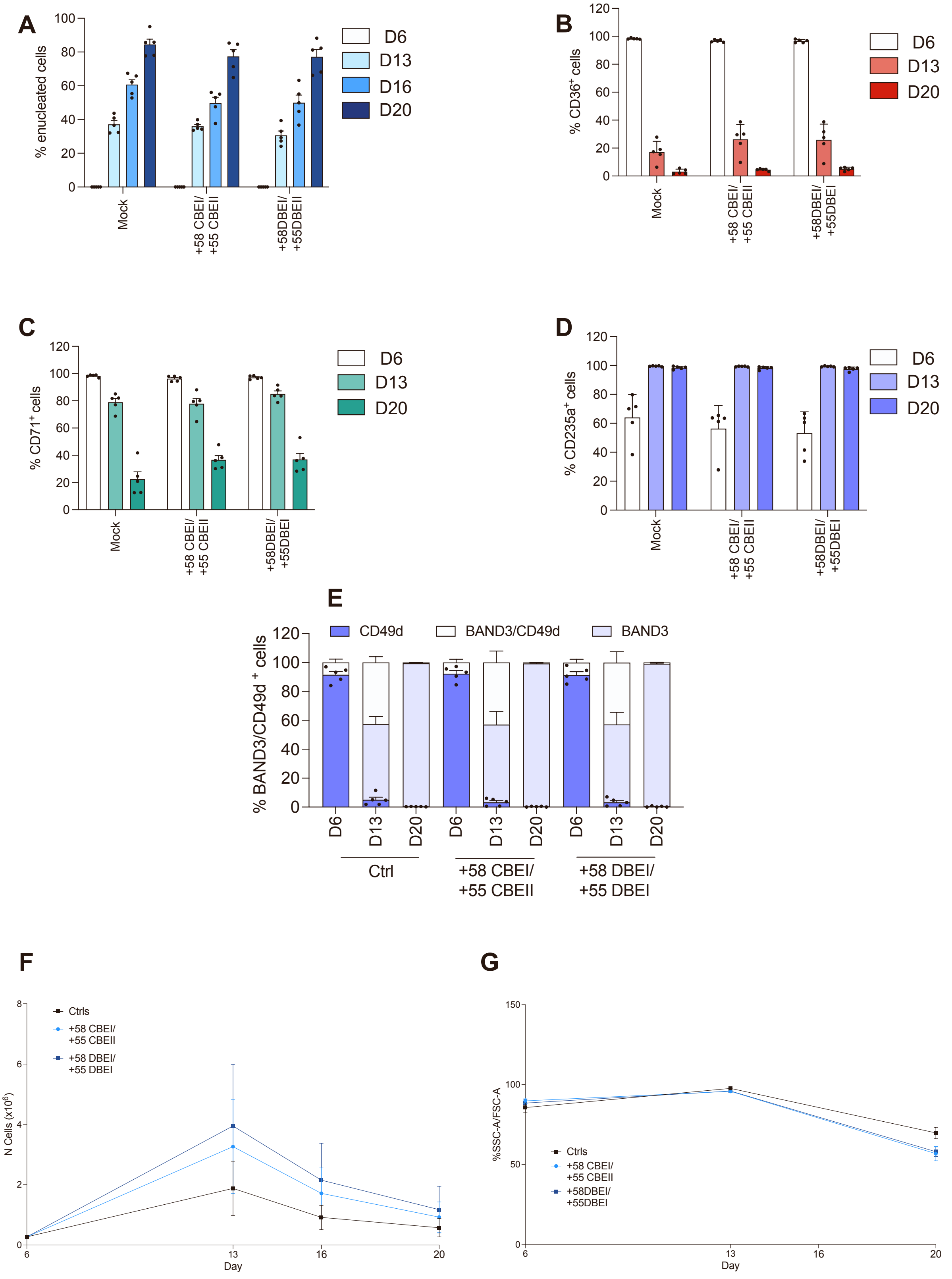
A
% enucleated cells
D6
D13
D16
D20
Mock
+58 CBEI/
+55 CBEII
+58DBEI/
+55DBEII
B
% CD36+ cells
D6
D13
D20
Mock
+58 CBEI/
+55 CBEII
+58DBEI/
+55DBEI
C
% CD71+ cells
D6
D13
D20
Mock
+58 CBEI/
+55 CBEII
+58DBEI/
+55DBEI
D
% CD235a+ cells
D6
D13
D20
Mock
+58 CBEI/
+55 CBEII
+58DBEI/
+55DBEI
E
% BAND3/CD49d + cells
CD49d
BAND3/CD49d
BAND3
D6
D13
D20
Ctrl
+58 CBEI/
+55 CBEII
+58 DBEI/
+55 DBEI
F
N Cells (x10^6)
Day
Ctrls
+58 CBEI/
+55 CBEII
+58 DBEI/
+55 DBEI
G
%SSC-A/FSC-A
Day
Ctrls
+58 CBEI/
+55 CBEII
+58DBEI/
+55DBEI

Figure S6. **Erythroid differentiation of SCD HSPCs upon multiplex base editing of the +58-kb and +55-kb regions.**

A. Frequency of enucleated cells at day 6, 13, 16, and 20 of erythroid differentiation, as measured by flow cytometry analysis of DRAQ5 nuclear staining in control and edited samples. Data are expressed as mean ± SEM (n = 1 biologically independent experiment, 5 donors).

B-D. Frequency of $CD36^+$ (**B**), $CD71^+$ (**C**), and $CD235a^+$ (**D**) cells at day 6, 13, and 20 of erythroid differentiation, as measured by flow cytometry analysis of CD36, CD71, and CD235a erythroid markers. Data are expressed as mean ± SEM (n = 1 biologically independent experiment, 5 donors).

E. Frequency of $CD49d^+$, $BAND3^+$ and $CD49d^+/BAND3^+$ in $7AAD^-/CD235a^+$ cells at day 6, 13, and 20 of erythroid differentiation, as measured by flow cytometry analysis of CD49d and BAND3 erythroid markers. Data are expressed as mean ± SEM (n = 1 biologically independent experiment, 5 donors).

F. Analysis of the cell growth over time. The number of cells was measured at multiple time points (day 6, 13, 16, and 20 of erythroid differentiation) to assess growth dynamics. Data are expressed as mean ± SEM (n = 1 biologically independent experiment, 5 donors).

G. Analysis of cell viability over time. We reported the percentage of live cells over the total live and dead/apoptotic cells identified based on size and granularity. Viability was assessed at days 6, 13, 16, and 20 of erythroid differentiation. Data are presented as mean ± SEM (n = 1 biologically independent experiment, from 5 donors).

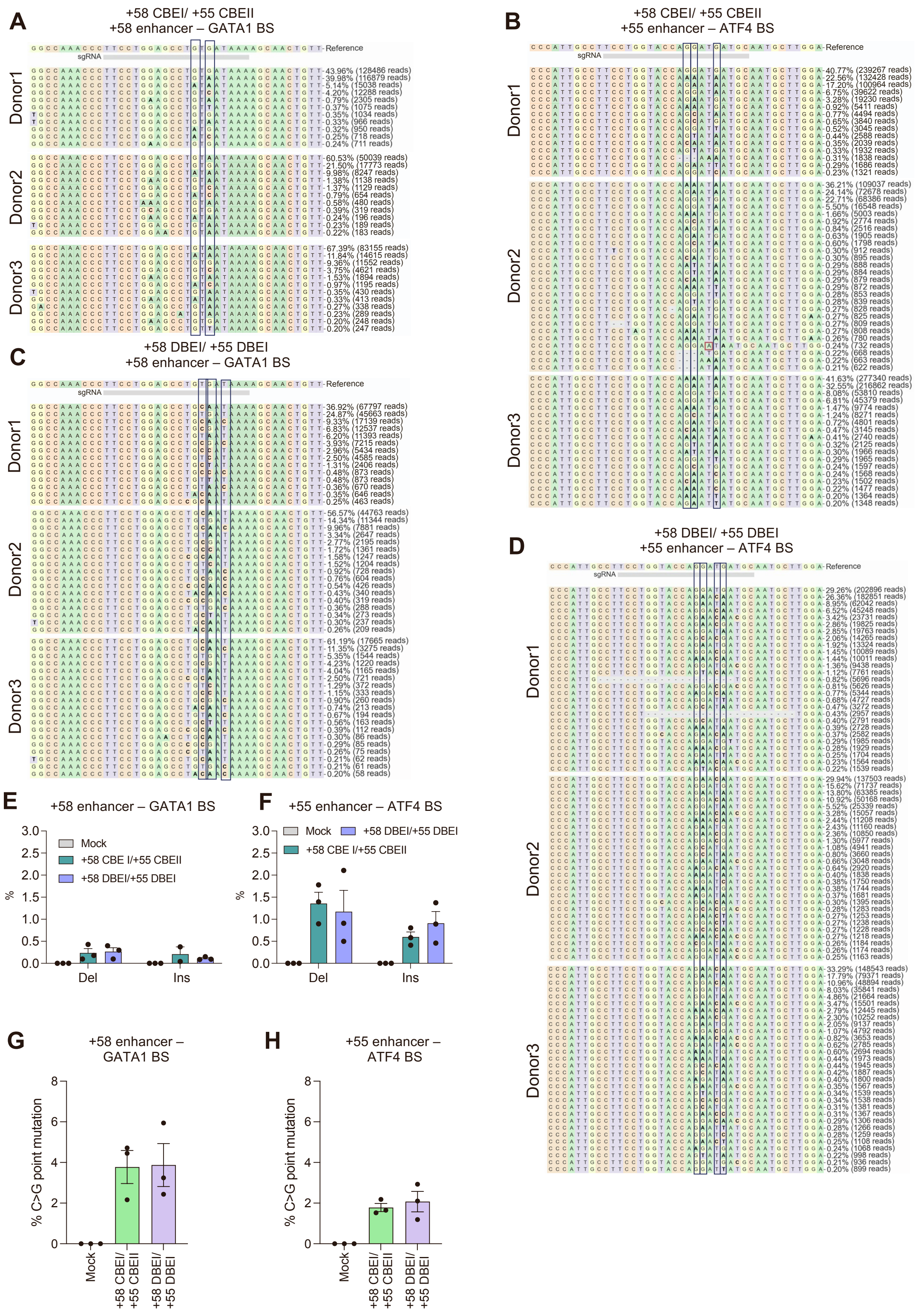

A
+58 CBEI/ +55 CBEII
+58 enhancer – GATA1 BS
Reference
sgRNA
Donor1
Donor2
Donor3
B
+58 CBEI/ +55 CBEII
+55 enhancer – ATF4 BS
Reference
sgRNA
Donor1
Donor2
Donor3
C
+58 DBEI/ +55 DBEI
+58 enhancer – GATA1 BS
Reference
sgRNA
Donor1
Donor2
Donor3
D
+58 DBEI/ +55 DBEI
+55 enhancer – ATF4 BS
Reference
sgRNA
Donor1
Donor2
Donor3
E
+58 enhancer – GATA1 BS
Mock
+58 CBE I/+55 CBEII
+58 DBEI/+55 DBEI
%
Del
Ins
F
+55 enhancer – ATF4 BS
Mock
+58 DBEI/+55 DBEI
+58 CBE I/+55 CBEII
%
Del
Ins
G
+58 enhancer – GATA1 BS
% C>G point mutation
Mock
+58 CBEI/ +55 CBEII
+58 DBEI/ +55 DBEI
H
+55 enhancer – ATF4 BS
% C>G point mutation
Mock
+58 CBEI/ +55 CBEII
+58 DBEI/ +55 DBEI

Figure S7. NGS analysis of on-target editing in erythroid cells derived from SCD HSPCs upon multiplex base editing of the +58-kb and +55-kb regions.

A-D. Frequency and sequence of modified and unmodified alleles in edited SCD samples, for +58 CBE**I**/ +55 CBE**II** (**A** and **B**) and +58 DBE**I**/ +55 DBE**I** (**C** and **D**) profiles at the +58-kb (**A** and **C**) and +55-kb (**B** and **D**) regions, as measured by targeted NGS. Target base positions are highlighted with a blue box. The red box indicates inserted bases. Grey squares indicate deletions. (n = 3 biologically independent experiments, 3 donors).

E-F. Deletions and **I**nsertions frequency in edited SCD samples for +58 CBE**I**/ +55 CBE**II** and +58 DBE**I**/ +55 DBE**I** profiles at the +58-kb (**E**) and +55-kb (**F**) regions, as measured by targeted NGS. Data are expressed as mean ± SEM (n = 3 biologically independent experiments, 3 donors).

G-H. C-G to G-C base-editing frequency in the +58-kb (**G**) or +55-kb (**H**) regions as measured by targeted NGS in SCD HSPCs simultaneously edited at the +58-kb and the +55-kb region. Data are expressed as mean ± SEM (n = 3 biologically independent experiments, 3 donors).

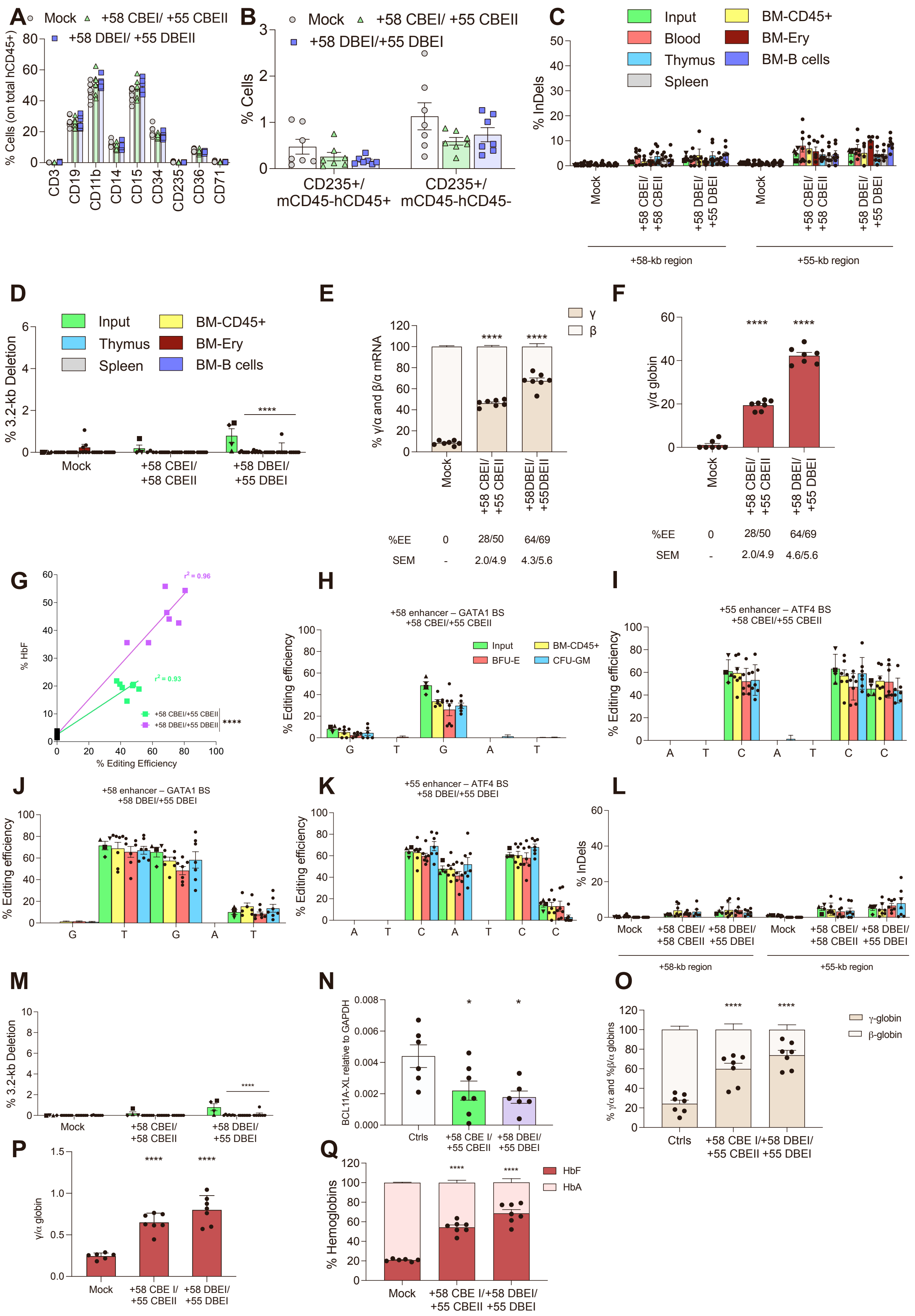

A
Mock
+58 CBEI/ +55 CBEII
+58 DBEI/ +55 DBEII
% Cells (on total hCD45+)
CD3 CD19 CD11b CD14 CD15 CD34 CD235 CD36 CD71
B
Mock
+58 CBEI/ +55 CBEII
+58 DBEI/+55 DBEI
% Cells
CD235+/ mCD45-hCD45+
CD235+/ mCD45-hCD45-
C
Input
Blood
Thymus
Spleen
BM-CD45+
BM-Ery
BM-B cells
% InDels
Mock
+58 CBEI/ +58 CBEII
+58 DBEI/ +55 DBEI
+58-kb region
+55-kb region
D
% 3.2-kb Deletion
Input
Thymus
Spleen
BM-CD45+
BM-Ery
BM-B cells
Mock
+58 CBEI/ +58 CBEII
+58 DBEI/ +55 DBEI
****
E
γ
β
% γ/α and β/α mRNA
Mock
+58 CBEI/ +55 CBEII
+58DBEI/ +55DBEI
%EE 0 28/50 64/69
SEM - 2.0/4.9 4.3/5.6
F
γ/α globin
Mock
+58 CBEI/ +55 CBEII
+58 DBEI/ +55 DBEI
%EE 0 28/50 64/69
SEM - 2.0/4.9 4.6/5.6
G
% HbF
% Editing Efficiency
$r^2 = 0.96$
$r^2 = 0.93$
+58 CBEI/+55 CBEII
+58 DBEI/+55 DBEII
****
H
+58 enhancer – GATA1 BS
+58 CBEI/+55 CBEII
% Editing efficiency
Input
BFU-E
BM-CD45+
CFU-GM
G T G A T
I
+55 enhancer – ATF4 BS
+58 CBEI/+55 CBEII
% Editing efficiency
A T C A T C C
J
+58 enhancer – GATA1 BS
+58 DBEI/+55 DBEI
% Editing efficiency
G T G A T
K
+55 enhancer – ATF4 BS
+58 DBEI/+55 DBEI
% Editing efficiency
A T C A T C C
L
% InDels
Mock
+58 CBEI/ +58 CBEII
+58 DBEI/ +55 DBEI
+58-kb region
+55-kb region
M
% 3.2-kb Deletion
Mock
+58 CBEI/ +58 CBEII
+58 DBEI/ +55 DBEI
****
N
BCL11A-XL relative to GAPDH
Ctrls
+58 CBE I/ +55 CBEII
+58 DBEI/ +55 DBEI
O
γ-globin
β-globin
% γ/α and %β/α globins
Ctrls
+58 CBE I/ +55 CBEII
+58 DBEI/ +55 DBEI
P
γ/α globin
Mock
+58 CBE I/ +55 CBEII
+58 DBEI/ +55 DBEI
Q
HbF
HbA
% Hemoglobins
Mock
+58 CBE I/ +55 CBEII
+58 DBEI/ +55 DBEI

Figure S8. Simultaneous base editing of *BCL11A* enhancers in repopulating HSCs and erythroid and granulo-monocytic progenitors derived from engrafting HSPCs.

A. Frequency of human T (CD3) and B (CD19) lymphoid, myeloid (CD11b, CD14, and CD15), HSPC (CD34), and erythroid (CD235a, CD36 and CD71) cells in BM in mice transplanted with control and edited HSPCs, 16/17 weeks after the transplantation (n = 7 mice per condition). Each data point represents an individual mouse. Data are expressed as mean ± SEM. (two-way ANOVA; not significant).

B. Frequency of human CD235a cells in mCD45-hCD45+ and mCD45-hCD45- BM populations in mice transplanted with control and edited HSPCs, 16/17 weeks after transplantation (n = 7 mice per condition). Each data point represents an individual mouse. Data are expressed as mean ± SEM. (two-way ANOVA; not significant).

C. Frequency of InDels calculated by TIDE for the +58 CBEI/+55 CBEII and +58 DBEI/+55 DBEI profiles at the +58-kb and +55-kb regions in the input, blood-, BM-, thymus-, and spleen-derived human samples subjected to Sanger sequencing. BM samples include CD45$^+$ cells, B cells, and erythroid cells (Ery). Data are expressed as mean ± SEM [n = 1 biologically independent experiment (Input), n = 4 to 7 mice per group]. Each data point represents an individual mouse (two-way ANOVA; not significant). The frequency of Indels in the input was calculated in cells cultured in the HSPC medium (■), in liquid erythroid cultures (▲), and pools of BFU-E (◆) and CFU-GM (▼).

D. Frequency of the 3.2-kb deletion, measured by ddPCR, for samples simultaneously edited at the +58-kb and +55-kb regions for the input, BM-, thymus-, and spleen-derived human samples. BM samples include CD45$^+$ cells, B cells, and erythroid cells (Ery). Data are expressed as mean ± SEM [n = 1 biologically independent experiment (Input), n = 7 mice per group]. Each data point represents an individual mouse. ****$P \leq 0.0001$ between the input and the different human hematopoietic populations derived from engrafted HSCs (two-way ANOVA with Tukey's correction for multiple comparisons). The frequency of 3.2-kb deletion in the input was calculated in cells cultured in the HSPC medium (■), in liquid erythroid cultures (▲), and in pools of BFU-E (◆) and CFU-GM (▼).

E. RT-qPCR analysis of γ ($^G\gamma$ + $^A\gamma$)- and β-globin mRNA in BM sorted human CD235a$^+$ erythroid cells in mice transplanted with control and edited HSPCs, 16/17 weeks post-transplantation. γ- and β^S-globin mRNA expression was normalized to α-globin mRNA and expressed as a percentage of the γ- + β^S-globin mRNA. The EE ± SEM is indicated for each sample in the lower part of the panel. Each data point represents an individual mouse. Data are expressed as mean ± SEM (n = 7 mice per group). ****$P \leq 0.0001$ (two-way ANOVA with Dunnett correction for multiple comparisons. Comparison mock vs edited samples).

F. Expression of γ ($^G\gamma$ + $^A\gamma$)-globin chains measured by RP-HPLC in BM-sorted human CD235a$^+$ erythroid cells in mice transplanted with control and edited HSPCs, 16/17 weeks post-transplantation. γ-globin expression was normalized to α-globin. The EE ± SEM is indicated for each sample in the lower part of the panel. Each data point represents an individual mouse. Data are expressed as mean ± SEM (n = 7 mice per group). ****$P \leq 0.0001$ (one-way ANOVA with Dunnett correction for multiple comparisons. Comparison mock vs edited samples).

G. Correlation between HbF expression and editing efficiency in human CD235a$^+$ BM-sorted erythroid cells obtained from mice transplanted with control and edited HSPCs. HbF was measured by cation-exchange HPLC and calculated over the total Hb tetramers. Base-editing efficiency was calculated using EditR software. ****$P \leq 0.0001$ (Multiple t test).

H-K. C-G to T-A or/and A-T to G-C base-editing efficiency, calculated using EditR for the +58 CBEI/ +55 CBEII (**H** and **I**) and +58 DBEI/ +55 DBEI (**I** and **J**) profiles at the +58-kb (**G** and **I**) and +55-kb (**J** and **K**) regions in the input, BM CD45$^+$ cells, and pooled BFU-E and CFU-GM derived from BM CD45$^+$ cells and subjected to Sanger sequencing. Data are expressed as mean ± SEM [n = 1 biologically independent experiment (Input), n = 7 mice per group]. Each data point represents an individual mouse. The frequency of base editing in the input was calculated in cells cultured in the HSPC medium (■), in liquid erythroid cultures (▲), and in pools of BFU-E (◆) and CFU-GM (▼).

L. Frequency of InDels calculated by TIDE for the +58 CBEI/+55 CBEII and +58 DBEI/+55 DBEI profiles at the +58-kb and +55-kb regions in the input, BM CD45$^+$ cells, and pooled BFU-E and CFU-GM derived from BM CD45$^+$ cells and subjected to Sanger sequencing. Data are expressed as mean ± SEM [n = 1 biologically independent experiment (Input), n = 7 mice per group]. Each data point represents an individual mouse (two-way ANOVA; not significant). The frequency of Indels in the input was calculated in cells cultured in the HSPC medium (■), in liquid erythroid cultures (▲), and in pools of BFU-E (◆) and CFU-GM (▼).

M. Frequency of the 3.2-kb deletion, measured by ddPCR, for samples simultaneously edited at the +58-kb and +55-kb regions for the input, BM CD45$^+$ cells, and pooled BFU-E and CFU-GM derived from BM CD45$^+$ cells. Data are expressed as mean ± SEM [n = 1 biologically independent experiment (Input), n = 7 mice per group]. Each data point represents an individual mouse. ****$P \leq 0.0001$ between the

input and the different human hematopoietic populations derived from engrafted HSCs (two-way ANOVA with Tukey's correction for multiple comparisons). The frequency of 3.2-kb deletion in the input was calculated in cells cultured in the HSPC medium (■), in liquid erythroid cultures (▲), and in pools of BFU-E (◆) and CFU-GM (▼).

N. RT-qPCR analysis of *BCL11A-XL* expression in pooled BFU-E derived from BM $CD45^+$ cells. *BCL11A-XL* mRNA expression was normalized to *GAPDH*. Data are expressed as mean ± SEM (n = 7 mice per group). Each data point represents an individual mouse. *P ≤ 0.05 (One-way ANOVA with Dunnett's correction for multiple comparison. Comparison of mock vs edited samples).

O. RT-qPCR analysis of γ ($^G\gamma$ + $^A\gamma$)- and β-globin mRNA in pooled BFU-E derived from BM $CD45^+$ cells. γ- and β^-globin mRNA expression was normalized to α-globin mRNA and expressed as a percentage of the γ- + β- β-globin mRNA. Each data point represents an individual mouse. Data are expressed as mean ± SEM (n = 7 mice per group). ****P ≤ 0.0001 (two-way ANOVA with Dunnett)

P. Expression of γ ($^G\gamma$ + $^A\gamma$)-globin chains measured by RP-HPLC in pooled BFU-E derived from BM $CD45^+$ cells. γ-globin expression was normalized to α-globin. Each data point represents an individual mouse. Data are expressed as mean ± SEM (n = 7 mice per group). ****P ≤ 0.0001 (one-way ANOVA with Dunnett correction for multiple comparisons. Comparison mock vs edited samples).

Q. Analysis of HbF and HbS by CE-HPLC in pooled BFU-E derived from BM $CD45^+$ cells. We calculated the percentage of each Hb type over the total Hb tetramers. Data are expressed as mean ± SEM (n = 7 mice per group). Each data point represents an individual mouse. ****P ≤ 0.0001 (two-way ANOVA with Dunnett correction for multiple comparisons. Comparison of mock vs edited samples).

SUPPLEMENTARY TABLES

sgRNA	Sequence (5' to 3')	Mismatches	Position (hg38)	Strand	Score	Type
GATA_bs_1	TTCCTCCCAGGCTCCAGGAAAGG	3	Chr1:150511390-150511412	-	0.55	Exonic
	CCTATCACTGGCTCCAGGAAGGG	3	Chr4:4338932-4338954	-	0.6	Intergenic
	CATTCACAGGCTCCAGGAATGG	2	Chr12:125257599-125257620	-	0.93	Intronic
	GTTATCTCAGCCTCCAGGAATGG	3	Chr18:45013184-45013206	-	1.05	Intronic
	TTTCTCAGAGCCTCCAGGAAGGG	3	Chr10:57611578-57611600	-	1.14	Intergenic
	CTGATCACAGCTCCAGGAAAGG	2	Chr19:42519879-42519900	-	1.28	Intronic
	TTTGTCTCAGCTCCAGGAAAGG	2	Chr1:49994908-49994929	-	1.41	Intronic
	TTTGTCTCAGCTCCAGGAAAGG	2	Chr3:3404786-3404807	-	1.41	Intronic
	TTGATCTCAGGCACCAGGAACGG	3	Chr3:11574097-11574119	+	1.48	Intronic
	CTTATCATAGGCCCCAGGAAAGG	2	Chr17:73712637-73712660	-	1.49	Intergenic
ATF4_bs_2	TCTTCTTCCTGGTACCAGGAGGG	3	Chr1:184873483-184873505	-	0.48	Intergenic
	GTACCTTCCTGGTACCAGGAAGG	3	Chr7:148128963-148128985	+	0.51	Intronic
	GTGTCAACCTGGTACCAGGATGG	3	Chr7:71049683-71049705	-	0.51	Intergenic
	GCGTCTTCCGGTACCAGGAGGG	2	ChrX:154158066-154158087	-	1.37	Intronic
	ACTCATCCTTGTACCAGGAAGG	2	Chr2:211197131-211197152	+	1.48	Intergenic
	GAAACATCCTGTACCAGGAGGG	2	Chr9:33373626-33373647	+	1.51	Intergenic
	GATCTTCCTTGTACCAGGAAGG	2	Chr11:16986847-16986868	+	1.55	Intronic
	GCAGCTCCTTGTACCAGGAAGG	2	Chr15:99988930-99988951	+	1.59	Intronic
	GCATCAGGCTGGCACCAGGAAGG	3	Chr16:50373084-50373106	-	1.6	Intergenic
	GCATCATCTAGTTACCAGGAAGG	3	Chr19:54317646-54317668	+	1.65	Intronic
	GCAGCCTCCTGGTGCCAGGAAGG	3	Chr14:105646275-105646297	+	1.68	Intergenic

Supplementary table 1. Predicted off-target sites.
Excel table containing the list of the top 5 GUIDE-seq predicted off-targets and of the top 5 *in silico* predicted off-targets.
