# Supplementary material for: Multiplex base editing of BCL11A regulatory elements to treat sickle cell disease
Source: Cell Rep Med. 2025 Sep 26;6(10):102376. doi: 10.1016/j.xcrm.2025.102376 (PMC12629801; doi:10.1016/j.xcrm.2025.102376)
Supplement: Document S2. Article plus supplemental information [file mmc4.pdf]

Cell Reports Medicine

Article

Multiplex base editing of *BCL11A* regulatory elements to treat sickle cell disease

Graphical abstract

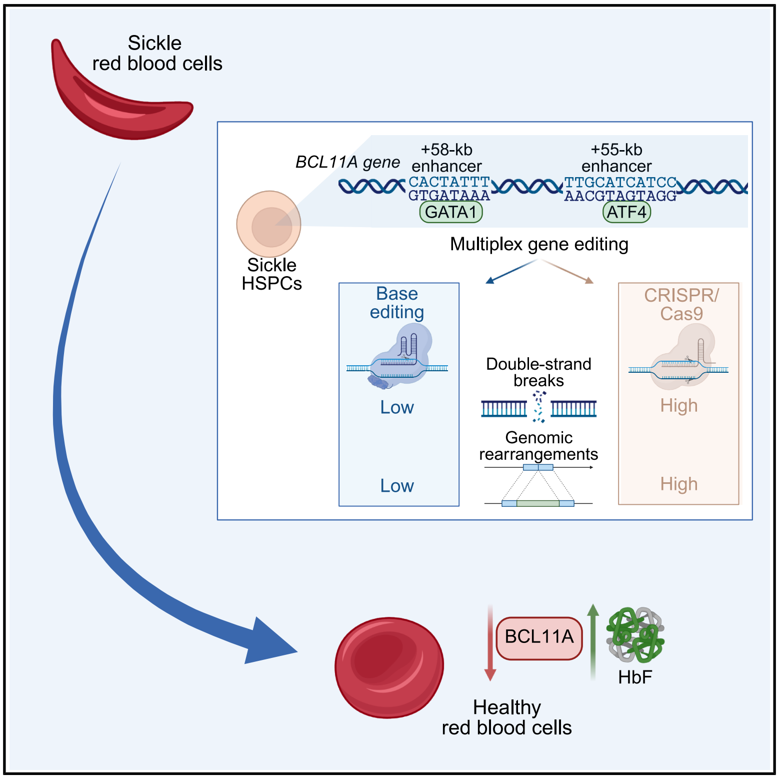

Highlights

- Base editing enables precise HbF tuning through the editing of critical DNA motifs
- Base editing of +58/+55 enhancers reduces *BCL11A* mRNA without generation of DSBs
- Combined editing of the +58-kb and +55-kb enhancers boosts HbF induction in RBCs
- Base editing is safe, efficient, and durable in long-term repopulating HSCs

Authors

Letizia Fontana, Pierre Martinucci, Simone Amistadi, ..., Mario Amendola, Panagiotis Antoniou, Annarita Miccio

Correspondence

pantonioy91@gmail.com (P.A.), annarita.miccio@institutimagine.org (A.M.)

In brief

Fontana et al. demonstrate that multiplex base editing of the *BCL11A* +58-kb and +55-kb enhancers disrupts two key transcriptional activator motifs and leads to robust HbF induction with minimal generation of double-strand breaks and genomic rearrangements. This approach is safe, efficient, and durable in long-term repopulating hematopoietic stem cells.

Fontana et al., 2025, Cell Reports Medicine 6, 102376
October 21, 2025 © 2025 The Author(s). Published by Elsevier Inc.
https://doi.org/10.1016/j.xcrm.2025.102376

CellPress

Cell Reports Medicine

CellPress
OPEN ACCESS

Article

Multiplex base editing of *BCL11A* regulatory elements to treat sickle cell disease

Letizia Fontana,[1] Pierre Martinucci,[1] Simone Amistadi,[1] Tristan Felix,[1] Margaux Mombled,[2,3] Alexandra Tachtsidi,[2,3] Guillaume Corre,[2,3] Anne Chalumeau,[1] Giulia Hardouin,[1] Jeanne Martin,[1] Oriana Romano,[4,6] Mario Amendola,[2,3,5,6] Panagiotis Antoniou,[1,*] and Annarita Miccio[1,7,*]

[1]Université Paris Cité, Imagine Institute, Laboratory of Chromatin and Gene Regulation During Development, INSERM UMR 1163, 75015 Paris, France

[2]Genethon, 91000 Evry, France

[3]Université Paris-Saclay, University Evry, Inserm, Genethon, Integrare Research Unit UMR_S951, 91000 Evry, France

[4]Department of Molecular Medicine, University of Padova, 35122 Padova, Italy

[5]Department of Clinical and Experimental Medicine, University of Foggia, 71122 Foggia, Italy

[6]These authors contributed equally

[7]Lead contact

*Correspondence: pantonioy91@gmail.com (P.A.), annarita.miccio@institutimagine.org (A.M.)

https://doi.org/10.1016/j.xcrm.2025.102376

SUMMARY

Sickle cell disease (SCD) is a genetic anemia caused by the production of an abnormal adult hemoglobin. Elevated levels of fetal hemoglobin (HbF) in adulthood reduce disease severity. A promising therapy involves the treatment of hematopoietic stem/progenitor cells (HSPCs) with CRISPR-Cas9 to downregulate the HbF repressor BCL11A via generation of double-strand breaks (DSBs) in the +58-kb enhancer. To improve safety and HbF induction, we use base editors to target both the +58-kb and +55-kb enhancers without generating DSBs. We dissect key DNA motifs recognized by transcriptional activators and identify critical nucleotides. Multiplex base editing efficiently disrupts these sites, reactivating HbF to levels exceeding those achieved with CRISPR-Cas9-induced editing, while minimizing DSBs and genomic rearrangements. Base editing is effective in long-term repopulating HSPCs and results in robust HbF reactivation *in vivo*. These findings demonstrate that multiplex base editing of *BCL11A* enhancers is a safe, efficient, and durable strategy to treat SCD.

INTRODUCTION

Sickle cell disease (SCD) is a highly prevalent recessive disorder caused by a single point mutation in the β-globin (*HBB*) gene, which leads to an amino acid substitution (Glu to Val) at position 6 of the β-globin chain. This sickle β-globin variant (β^S) combines with α-chains to form sickle hemoglobin (HbS), which tends to polymerize in low oxygen conditions, causing red blood cells (RBCs) to adopt a sickle shape and lose flexibility. Clinical manifestations are due to the short lifespan of sickle RBCs (leading to anemia) and to the obstruction of small blood vessels, causing multi-organ damage. Therefore, patients with SCD have a poor quality of life and reduced life expectancy.[1,2] Patients with SCD who lack a compatible donor for allogeneic hematopoietic stem/progenitor cell (HSPC) transplantation can benefit from gene therapy approaches based on the transplantation of autologous, genetically modified HSPCs.[3]

The severity of SCD is alleviated by the production of gamma-globin (γ-globin) chains, which compose the HbF. γ-globin exerts a critical anti-sickling effect and competes with β^S-globin for incorporation into the hemoglobin tetramer, thereby reducing the formation of HbS.[4] Thus, transplantation of autologous HSPCs genetically modified to re-express HbF in their erythroid progeny is a treatment option for patients with SCD. Currently, different approaches aiming to reactivate HbF have been developed. CRISPR-Cas9 nuclease has been used to efficiently disrupt binding sites (BSs) for transcriptional repressors in the γ-globin genes (*HBG1/HBG2)* promoters, leading to restoration of HbF expression and rescue of the sickle phenotype.[5–7] Furthermore, CRISPR-Cas9 nuclease-mediated downregulation of *BCL11A,* a major γ-globin repressor,[8] was recently approved as therapy for the treatment of β-hemoglobinopathies.[9,10] This approach targets the GATA1 activator BS within the +58-kb *BCL11A* erythroid-specific enhancer to reduce *BCL11A* expression exclusively in erythroid cells, thereby reactivating HbF production. Alternatively, targeting the ATF4 activator BS within the +55-kb *BCL11A* erythroid-specific enhancer represents another strategy to induce HbF expression in adult cells.[11]

However, the clinical study targeting the +58-kb enhancer showed variability in the extent of HbF reactivation among individuals, relatively low levels of Hb with HbS still accounting for a large proportion of the total Hb, and modest correction of ineffective erythropoiesis.[9,10] Therefore, we hypothesize that simultaneous editing of the +58-kb and +55-kb enhancers could

This is an open access article under the CC BY license (http://creativecommons.org/licenses/by/4.0/).

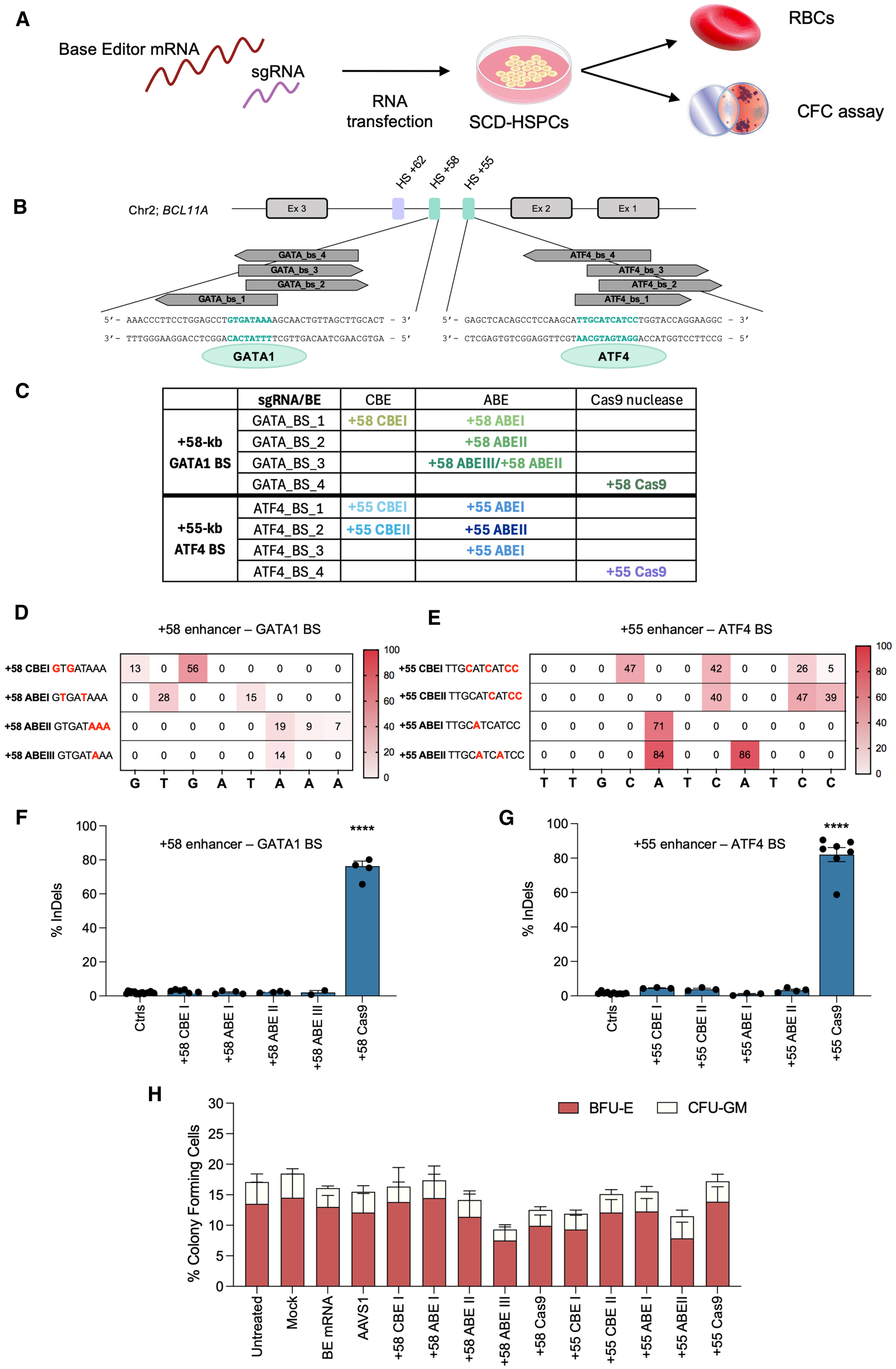

(legend on next page)

maximize *BCL11A* downregulation, resulting in higher and more consistent HbF levels. To avoid DSB-induced toxicity and the generation of large genomic rearrangements associated with the use of CRISPR-Cas9 nuclease,[12,13] we used base editors (BEs) to precisely target the GATA1 and ATF4 activator BS in the +58-kb and +55-kb *BCL11A* enhancers. In particular, we exploited cytosine BEs (CBEs), adenine BEs (ABEs), and dual BEs (DBEs) to dissect the GATA1 and ATF4 BSs in SCD HSPCs and identify the critical base conversions that induce changes in enhancers activity, *BCL11A* downregulation, and, consequently, HbF reactivation.

RESULTS

Base editing disrupts the +58-kb and +55-kb *BCL11A* enhancers without affecting SCD HSPC viability and differentiation

To disrupt the *BCL11A* erythroid enhancers, SCD HSPCs were transfected with BE mRNAs and single guide RNAs (sgRNAs) targeting the +58-kb and +55-kb regions (Figure 1A), introducing various point mutations at the GATA1 and ATF4 BS, respectively (Figure 1B). We evaluated the base editing efficiency in erythroblasts derived from transfected HSPCs. At both the +58-kb and +55-kb regions, different combinations of CBEs/ABEs and sgRNAs led to the generation of several editing profiles (Figures 1B and 1C). In the +58-kb region, we generated the +58 CBEI editing profile using CBE (GTGATAAA; targeted nucleotides are underlined) or the +58 ABEI profile (GTGATAAA) using ABE in combination with a sgRNA (GATA_BS_1), previously used to target the GATA1 BS (sg1620)[14–18] and selected as the most effective in reactivating HbF among those targeting the first nucleotides of the GATA1 BS. Furthermore, we generated the novel +58 ABEII (GTGATAAA) and +58 ABEIII profiles (GTGATAAA), which contain mutations in the last nucleotides of the GATA1 BS. These profiles were not reported in previous works and may exhibit enhanced efficacy in HbF reactivation (Figures 1B–1D). In the +55-kb region, CBEs led to the +55 CBEI (TTGCATCATCC) and +55 CBEII profiles (TTGCATCATCC), while ABEs generated the +55 ABEI (TTGCATCATCC) and the +55 ABEII profiles (TTGCATCATCC; Figures 1C–1E). For comparison, we transfected Cas9 nuclease ribonucleoprotein particle (RNP) complexes, which disrupted either the +58-kb[8] or the +55-kb[11] regions through InDel generation (Figures 1F and 1G). Low or no InDels were detected in base-edited samples, confirming the DSB-low nature of BEs (Figures 1F and 1G). A colony-forming cell (CFC) assay showed no significant differences in erythroid (BFU-E) or granulocyte/monocyte (CFU-GM) colonies between control and edited samples, despite a tendency for a lower number in +58 ABEIII and +55 ABEII samples (Figure 1H). The base editing efficiency and InDel profiles in pools of BFU-E and CFU-GM were similar to those measured in liquid erythroid cultures (Figures S1A–S1H). In conclusion, we efficiently targeted the GATA1 and ATF4 BSs at the +58-kb and +55-kb *BCL11A* enhancer regions with base editing strategies without affecting HSPCs' viability and differentiation potential.

HbF reactivation after base editing of *BCL11A* enhancers in SCD HSPCs

We differentiated transfected SCD HSPCs in liquid culture toward the erythroid lineage. Flow cytometry showed no differences in enucleation ore expression of early and late erythroid markers between control and edited groups, indicating preserved erythroid differentiation (Figures S2A–S2E). We assessed *HBG* reactivation at RNA and protein levels by quantitative reverse-transcription PCR (RT-qPCR), reverse phase high-performance liquid chromatography (RP-HPLC), and cation exchange high performance liquid chromatography (CE-HPLC) (Figures 2A–2C). Among the +58-kb edits, the +58 CBEI profile expressed the highest HbF levels among base-edited groups and was only modestly lower than the +58 Cas9 profile, despite the significantly lower editing efficiency (56.0% ± 4.7 and 76% ± 3.0 in +58 CBEI and +58 Cas9, respectively). ABE-treated samples exhibited low HbF levels, consistent with limited editing efficiency (Figures 2A–2C). Interestingly, the +58 ABEIII profile showed HbF levels comparable to ABEI and ABEII, despite the lowest editing, suggesting that this base conversion is potentially highly productive in terms of HbF (Figures 2A–2C).

Figure 1. Base editing of the erythroid-specific *BCL11A* enhancers in SCD HPSC-derived erythroblasts

(A) Experimental protocol used for base-editing experiments in SCD HSPCs. SCD HSPCs were transfected with BE and sgRNAs. Cells were differentiated into mature RBCs in liquid culture or subjected to a CFC assay.

(B) Scheme of *BCL11A* exons 2 to 4 (Ex 2, 3, and 4) and the DNaseI hypersensitive sites (HS) +62-kb, +58-kb, and +55-kb. The sequences of the +58-kb and +55-kb *BCL11A* enhancers are shown, with GATA1 and ATF4 BSs in green. Arrows indicate sgRNAs used in combination with BEs or Cas9, aligned to their DNA targets. Green ovals indicate GATA1 and ATF4.

(C) Table showing combinations of BEs and sgRNAs generating the different editing profiles. GATA_bs_1 sgRNA was used in combination with CBE-SpRY or AncBE4max and ABEmax-SpRY to generate the +58 CBEI and +58 ABEI profile, respectively. GATA_bs_2 sgRNA was coupled with NG-ABEmax and ABEmax-SpRY to generate the +58 ABEII profile, while GATA_bs_3 sgRNA/ABEmax-SpRY generates the +58 ABEIII profile. GATA_BS_4 was combined with Cas9 nuclease to generate the +58 Cas9 profile. CBE-SpRY and AncBE4max coupled with ATF4_bs_1 or ATF4_bs_2 sgRNAs led to +55 CBEI and +55 CBEII profiles, respectively. ATF4_bs_1 sgRNA or ATF4_bs_3 combined with ABEmax-SpRY led to +55 ABEI profile, and ATF4_bs_2 sgRNA combined with ABE8e-SpRY led to +55 ABEII profile. ATF4_BS_4 was combined with Cas9 nuclease to generate the +55 Cas9.

(D and E) C-G to T-A or A-T to G-C base-editing efficiency in erythroblasts derived from SCD HSPCs edited in the +58-kb (D) or +55-kb (E) regions.

(F and G) Frequency of InDels, in erythroblasts derived from SCD HSPCs edited in the +58-kb (F) or +55-kb (G) regions.

For (D–G), data are expressed as mean (± SEM in F and G) (n = 2 to 6 biologically independent experiments, 2 to 5 donors). ****$p \leq 0.0001$. One-way ANOVA. Comparison of controls vs. edited samples. No asterisk (*) = not significant.

(H) CFC frequency for controls (untreated, or transfected with TE buffer, or transfected with a BE mRNA only, or transfected with a BE mRNA and a sgRNA targeting the unrelated *AAVS1* locus) and edited samples.

Data are expressed as mean ± SEM (n = 7 biologically independent experiments, 7 donors). Two-way ANOVA with Dunnett's correction for multiple comparisons; not significant.

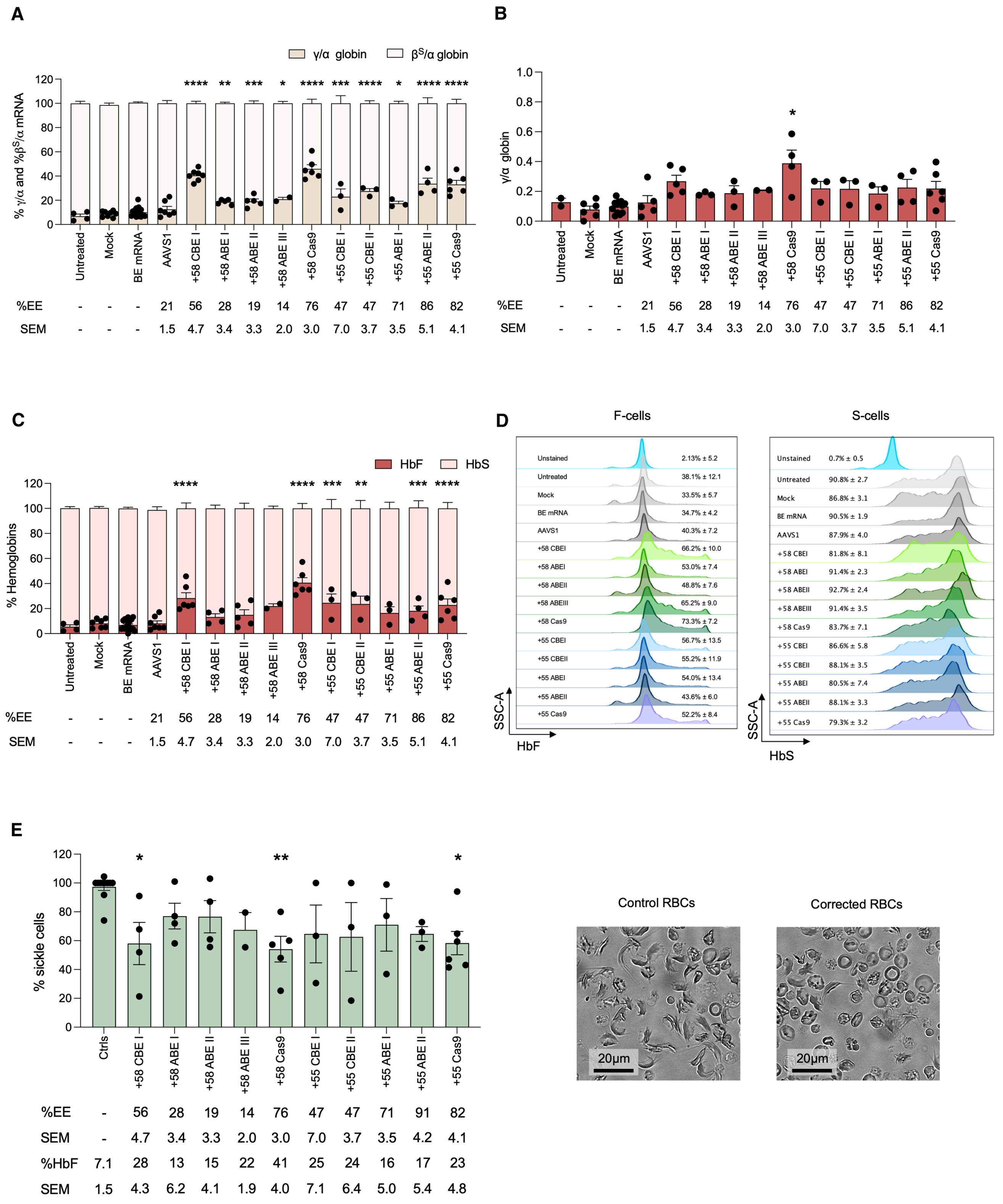

Figure 2. HbF reactivation and correction of the sickle phenotype in edited SCD HPSC-derived erythroblasts

(A) RT-qPCR analysis of γ (^{G}γ + ^{A}γ)- and β^{S}-globin mRNA in control and edited SCD erythroblasts at day 13 of erythroid differentiation.

(B) Expression of γ (^{G}γ + ^{A}γ)-globin chains measured by RP-HPLC in control and edited SCD HSPC-derived RBCs.

(legend continued on next page)

Concerning the +55-kb region, CBE and ABE reactivated HbF to levels comparable to those in Cas9-treated samples, even though the CBE editing frequency was around 2-fold lower compared to ABE and Cas9 (Figures 2A–2C).

Similar results in terms of HbF reactivation were also observed in pools of BFU-E (Figure S1I). Flow cytometry confirmed an increase in HbF+ (F-cells) and slight decrease in HbD+ (S-cells) populations (Figure 2D). Finally, a sickling assay revealed reduced sickle cell frequency particularly in +58 CBEI, +58 Cas9, and +55 Cas9 samples (Figure 2E). In summary, base editing of GATA1 and ATF4 BS enhanced HbF, though the HbF levels in the best case were only approaching those achieved with the approved +58 Cas9 strategy. Phenotypic rescue was incomplete and variable across donors, highlighting the need for optimization by further increasing HbF expression.

Dissecting GATA1 and ATF4 binding motifs to achieve robust HbF reactivation

To evaluate the potency of the different editing profiles, we correlated γ-globin expression and editing efficiency at the clonal level in BFU-Es from edited SCD HSPCs (Figures 3A and 3B). Despite some variability in γ-globin reactivation among samples with similar editing efficiency (likely due to assay-intrinsic factors),[5,19,20] we observed a positive correlation between editing efficiency and γ-globin reactivation. For the +58-kb enhancer, the +58 ABEIII profile showed the highest γ-globin reactivation, outperforming the +58 Cas9 profile (Figure 3A). Attempts to improve the +58 ABEIII editing efficiency (using ABE8e,[14] instead of the first-generation ABEmax) resulted in the generation of the +58 ABEII profile with 80.5% ± 0.7 efficiency but a higher InDel frequency (4.4% ± 2.8; Figures 3C and 3D). Conversely, the +58 ABEI and ABEII profiles generated more bi-allelic colonies (due to higher editing efficiency), making them more suitable for future experiments (Figure 3A). Among these, +58 ABEII was selected for further testing due to the higher frequency of bi-allelic colonies and the better correlation between editing frequency and *HBG1/2* levels. The results emphasize the importance of specific bases in the GATA1 BS for GATA1 binding and γ-globin regulation. Targeting A6 (via +58 ABEIII) significantly increased γ-globin expression, while targeting A6 to A8 (via +58 ABEII) has a more modest effect. In addition, T>C conversions at T2 and T5 (+58 ABEI) were less effective than targeting A6, suggesting they have a weaker impact on GATA1 binding (Figure 3A). Finally, The +58 CBEI profile showed γ-globin reactivation similar to the +58 Cas9 profile, confirming the critical role of G in position 3 for GATA1 binding[21]; thus, the +58 CBEI profile was also chosen for further testing (Figure 3A).

For the +55-kb enhancer, all the profiles exhibited comparable γ-globin reactivation (except for the +55 ABEI showing the lowest *HBG1/2* levels). The +55 CBEII and +55 ABEII profiles showed a better correlation between editing frequency and *HBG1/2* levels and were selected for further testing (Figure 3B). These data suggest that insertion of a C>T conversion at C4 (+55 CBEI, TTGCATCATCC) does not impact the ATF binding more than targeting only the last 3 Cs in the motif (+55 CBEII; TTGCATCATCC). Furthermore, A>G mutations in the +55 ABEI and +55 ABEII profiles (particularly the selective targeting of A5 in the +55 ABEI profile) have a lower impact on ATF4 binding.

Enhanced γ-globin reactivation through multiplex editing

We hypothesized that simultaneous editing of the +58-kb and +55-kb enhancers could lead to a stronger *BCL11A* downregulation, and greater HbF induction compared to enhancer editing of individual enhancers (Figures 3A, 3B, 4A, and 4B). Modestly reduced editing efficiencies were observed when inserting the combination of +58 ABEII and +55 ABEII profiles compared to samples transfected with the individual ABE/sgRNA combinations (Figures 4C and 4D). We then used CBEs to insert the most potent CBE profiles, +58 CBEI and +55 CBEII, reaching editing efficiencies similar to those achieved by single editing (Figures 4C and 4D). We also used a recently developed, dual function, cytosine and ABE (TadDE)[17] to simultaneously introduce C>T and A>G point mutations at the GATA1 and ATF4 BSs, generating two additional profiles (+58 DBEI: GTGATAAA and +55 DBEI: TTGCAT CATCC), reaching high editing efficiencies upon single or multiplex editing (Figures 4C and 4D). Importantly, base-edited samples showed no or few InDels (Figures 4E and 4F). In parallel, we used Cas9 nuclease to simultaneously target both enhancers. The efficiency of concomitant generation of the +58 and +55 Cas9 profiles was similar to that obtained upon single Cas9 editing (Figures 4E and 4F). This approach potentially generates a 3.2-kb deletion or inversion that disrupts the targeted GATA1 and ATF4 BSs and removes additional BSs for transcriptional activators (Figure 4G). ddPCR showed 33.1% ± 8.1 of deletion and 10.4% ± 2.3 of inversion for the +58/+55 Cas9 profile (Figure 4H). No deletion or inversion was detected with the +58 ABEII/+55 ABEII profile, and only 0.5% ± 0.4 and 1.1% ± 0.9 of deletion were detected for +58 CBEI/+55 CBEII and +58 DBEI/+55 DBEI profiles, respectively (Figure 4H). Although we observed a modest (but non-significant) decrease in cell growth, viability, and enucleation upon single or dual editing, expression of erythroid markers was similar across control and edited groups (Figures S3A–S3G).

(C) Analysis of HbF and HbS expression measured by cation-exchange HPLC in control and edited SCD HSPC-derived RBCs.

(D) Representative flow cytometry histograms showing the percentage of HbF-expressing cells (F-cells) and HbS-expressing cells (S-cells) in the $CD235a^+$ population for unstained (CD235a stained only), control, and edited samples.

(E) Frequency of sickling cells upon O_2 deprivation in control and edited samples. The EE ± SEM is indicated for each sample in the lower part of the panel. Representative photomicrographs of SCD patient RBCs under hypoxic conditions are shown. Scale bars, 20 μm.

For (A–C and E): editing efficiency (EE) ± SEM is indicated for each sample. Data are expressed as mean ± SEM ($n = 2$ to 6 biologically independent experiments, 2 to 5 donors). Statistical comparisons (mock vs. edited samples) were performed using one-way or two-way ANOVA with Dunnett's correction. *$p \leq 0.05$, **$p \leq 0.01$, ***$p \leq 0.001$, and ****$p \leq 0.0001$.

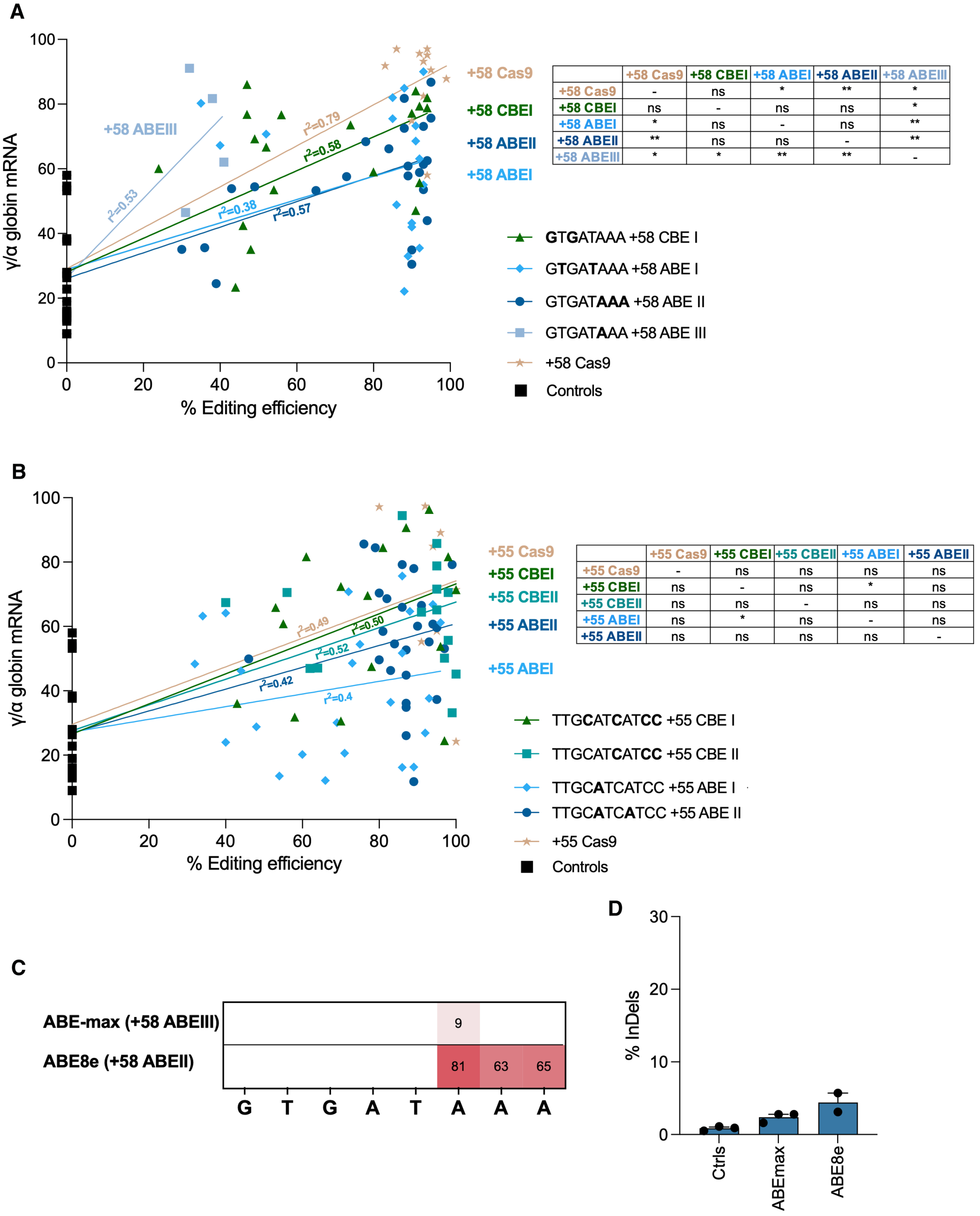

	+58 Cas9	+58 CBEI	+58 ABEI	+58 ABEII	+58 ABEIII
+58 Cas9	-	ns	*	**	*
+58 CBEI	ns	-	ns	ns	*
+58 ABEI	*	ns	-	ns	**
+58 ABEII	**	ns	ns	-	**
+58 ABEIII	*	*	**	**	-

	+55 Cas9	+55 CBEI	+55 CBEII	+55 ABEI	+55 ABEII
+55 Cas9	-	ns	ns	ns	ns
+55 CBEI	ns	-	ns	*	ns
+55 CBEII	ns	ns	-	ns	ns
+55 ABEI	ns	*	ns	-	ns
+55 ABEII	ns	ns	ns	ns	-

Figure 3. Gamma-globin reactivation in single BFU-E colonies derived from edited SCD HSPCs

(A and B) Correlation between *HBG* mRNA relative expression and editing efficiency in single BFU-E colonies differentiated from SCD HSPCs edited in the +58-kb (A) or +55-kb (B) regions (1 donor). Different editing profiles were generated through the combination of different BE and sgRNA, similar to Figures 1C and 1D mRNA expression was measured by RT-qPCR. BFU-Es transfected with TE buffer, or transfected with a BE mRNA only, or transfected with a BE mRNA and a sgRNA targeting the unrelated AAVS1 locus were used as negative controls. Statistical significances are reported in the tables: $^*p \leq 0.05$ and $^{**}p \leq 0.01$ (multiple t test).

(C) A-T to G-C base-editing efficiency measured in pools of BFU-E derived from SCD HSPCs edited in the +58-kb region.

(D) Frequency of InDels measured in pools of BFU-E colonies derived from SCD HSPCs edited in the +58-kb. $^{**}p \leq 0.01$. One-way ANOVA comparison. Statistical significance between mock and edited samples is depicted in the graph.

For (C and D), data are expressed as mean ± SEM (n = 2 to 3 biologically independent experiments, 2 to 3 donors). The GATA_bs_2 sgRNA was combined with NG-ABE-max and NG-ABE8e.

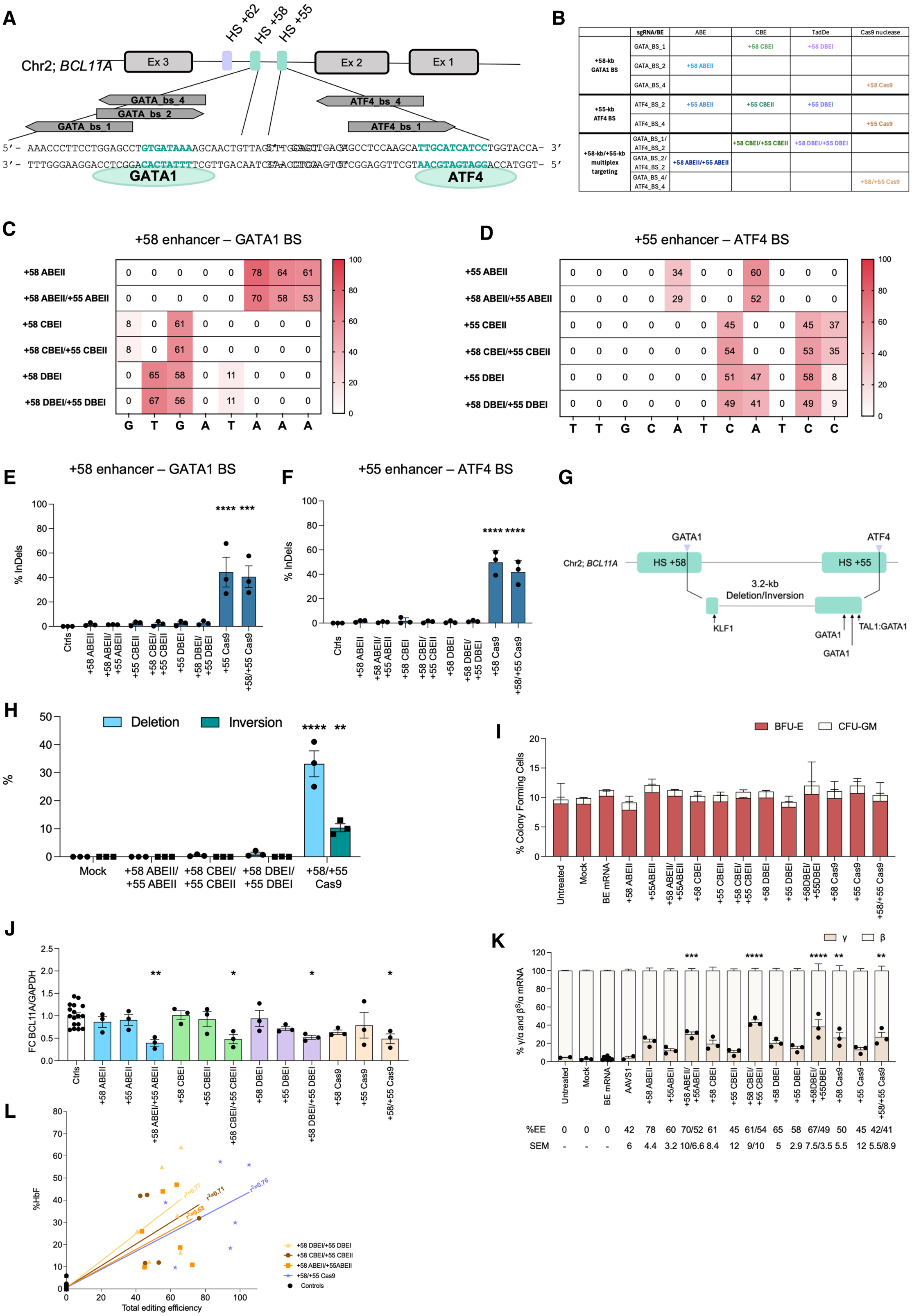

	sgRNA/BE	ABE	CBE	TadDe	Cas9 nuclease
+58-kb GATA1 BS	GATA_BS_1		+58 CBEI	+58 DBEI	
	GATA_BS_2	+58 ABEII			
	GATA_BS_4				+58 Cas9
+55-kb ATF4 BS	ATF4_BS_2	+55 ABEII	+55 CBEII	+55 DBEI	
	ATF4_BS_4				+55 Cas9
+58-kb/+55-kb multiplex targeting	GATA_BS_1/ ATF4_BS_2		+58 CBEI/+55 CBEII	+58 DBEI/+55 DBEI	
	GATA_BS_2/ ATF4_BS_2	+58 ABEII/+55 ABEII			
	GATA_BS_4/ ATF4_BS_4				+58/+55 Cas9

(legend on next page)

A CFC assay demonstrated no difference in terms of viability and clonogenic potential between control, single-, and multiplex-edited SCD HSPCs (Figure 4I). The frequency of base editing, InDel, and 3.2-kb deletion/inversion in the pools of BFU-E and CFU-GM populations was similar to that measured in the liquid erythroid cultures (Figures S4A–S4J).

Overall, multiplex editing led to greater down-regulation of *BCL11A-XL* (encoding the isoform responsible for HbF silencing) and an increase of γ-globin production compared to single editing, regardless of the specific editor used (Figures 4J, 4K, and S4K–S4M). To circumvent the disparity in editing efficiency between the samples, we correlated HbF reactivation and editing efficiency obtained in erythroid liquid cultures and pools of BFU-E (Figures 4L and S5A–S5D). This analysis confirmed that multiplex base editing correlated with stronger HbF reactivation compared to single editing. On the contrary, no additional effect was observed when targeting both BSs with the Cas9 nuclease compared to editing the +58-kb enhancer alone (Figures S5A–S5D). A comparison of multiplex edited samples showed a significantly higher potency of DBE, followed by CBEs, ABEs, and lastly Cas9 (Figure 4K).

CBE- and DBE-mediated multiplex targeting of *BCL11A* efficiently rescues the sickling phenotype

We selected CBE and DBE for further efficacy and safety analyses, as they showed higher HbF reactivation compared to ABE. SCD HSPCs were co-transfected with the AncBE4max CBE-[22] or the TadDE DBE-[17] mRNAs and the same sgRNAs. While most of the enzymes used in the screening phase (Figures 1, 2, and S1) are nearly protospacer adjacent motif (PAM)-less, both these BEs recognize an NGG PAM, to reduce the occurrence of off-targets.[23] Flow cytometry revealed no significant differences in enucleation or expression of erythroid markers, with only a mild reduction in cell growth and viability in the edited samples (Figure S6). Editing efficiency in erythroid liquid cultures was similar with CBE and DBE at both GATA1 and ATF4 (Figures 5A and 5B). Next-generation sequencing (NGS) analysis confirmed high editing efficiency and the expected base modifications (Figures S7A–S7D) with rare C>G and InDel events for both CBE and DBE (Figures 5C, 5D, and S7A–S7H). As previously observed, +58 DBEI/+55 DBEI profile showed a higher frequency of 3.2-kb deletion (1.2% ± 0.5) compared to the +58 CBEI/+55 CBEII profile (0.3% ± 0.2, Figure 5E).

A marked *BCL11A-XL* reduction (Figure 5F) and significant γ-globin reactivation were detected in the edited samples (Figure 5G). This was confirmed at the protein level in mature RBCs by analyzing both globin chains and hemoglobin tetramers, with HbF accounting for 29.1% ± 3.6 and 28.3% ± 9.8 of total hemoglobin in the +58 CBEI/+55 CBEII and +58 DBEI/+55 DBEI profile, respectively (Figures 5H and 5I). Additionally, more HbF-expressing cells and a significant reduction in HbS-expressing cells were detected in edited samples (Figure 5J), with only 43.0% ± 15.0% and 38.7% ± 10.5% sickle cells. Similarly, an increase of HbF-expressing cells was observed by generating the +58 CBEI/+55 CBEII and +58 DBEI/+55 DBEI profiles (Figure 5J), and importantly, a significant and homogeneous reduction in the frequency of HbS-expressing cells was observed in the edited samples compared to controls (Figure 5J). Finally, only 43.0% ± 15.0 and 38.7% ± 10.5 of sickle cells were detected in CBE- and DBE-edited samples, respectively (Figure 5K). These frequencies are similar to those observed in heterozygous, asymptomatic SCD carriers.

CBE- and DBE-mediated multiplex targeting of *BCL11A*: Genotoxicity assessment

To evaluate the locus integrity, we performed long-read sequencing of the region encompassing the GATA1 and ATF4 BSs. Editing efficiencies between 27% and 43% were achieved for the +58 CBEI/+55 CBEII, +58 DBEI/+55 DBEI, and +58/+55 Cas9 profiles (Figures 6A and 6B). As expected, lower frequencies of alleles with 3.2-kb deletion/inversion were

Figure 4. Multiplex base editing of the erythroid-specific *BCL11A* enhancers in SCD HPSC-derived erythroblasts

(A) Schematic representation of *BCL11A* exons and the +58-kb and +55-kb regions. sgRNAs used in combination with BEs or Cas9 nuclease are indicated with arrows.

(B) Table showing combinations of BEs and sgRNAs generating the different editing profiles. GATA_bs_2 or/and ATF4_bs_2 sgRNAs were combined with NG-ABE8e to generate +58 ABEII, +55 ABEII, and +58 ABEII/+55 ABEII profiles. GATA_bs_1 or/and ATF4_bs_2 sgRNAs combined with AncBE4max generated the +58 CBEI, +55 CBEII, and +58 CBEI/+55 CBEII profiles. GATA_bs_1 or/and ATF4_bs_2 sgRNAs combined with TadDE generated the +58 DBEI, +55 DBEI, and +58 DBEI/+55 DBEI profiles. Finally, GATA_BS_4 and/or ATF4_BS_4 were used in combination with Cas9 nuclease to generate +58 Cas9 and +55 Cas9, respectively, and in combination to generate the +58/+55 Cas9 profile.

(C and D) C-G to T-A or/and A-T to G-C base-editing efficiency in erythroblasts derived from SCD HSPCs edited at the +58-kb (C) or +55-kb (D) regions, or both loci simultaneously.

(E and F) Frequency of InDels in the +58-kb (E) or +55-kb (F) regions in samples described in (C and D).

(G) Schematic representation of the generation of a 3.2-kb deletion/inversion following the simultaneous targeting of GATA1 BS in the +58-kb region and ATF4 BS in the +55-kb region (highlighted with violet arrows). Black arrows indicate the location of BSs for additional transcriptional activators.

(H) Frequency of the 3.2-kb deletion/inversion, measured by ddPCR, for samples simultaneously edited at the +58-kb and +55-kb regions.

(I) CFC frequency for control and edited samples. Data are expressed as mean ± SEM ($n = 2$ biologically independent experiments, 2 donors).

(J) ddPCR analysis of *BCL11A* expression in erythroblasts derived from edited SCD HSPCs at day 13 of erythroid differentiation.

(K) RT-qPCR analysis of γ ($^{G}\gamma + {}^{A}\gamma$)- and β^{S}-globin mRNA in control and edited SCD erythroblasts at day 13 of erythroid differentiation. The EE ± SEM is indicated for each sample.

(L) Correlation between HbF expression (measured by cation-exchange HPLC) and total editing efficiency (base-editing or Cas9 editing and the 3.2-kb deletion and inversion) in erythroid samples and BFU-E pools derived from edited SCD HSPCs. The control background was subtracted.

For (G and H) and (J–L), data are expressed as mean ($n = 3$ biologically independent experiments, 3 donors). Statistical analyses were performed using one-way or two-way ANOVA with Dunnett's or Tukey's correction for multiple comparisons, or multiple t tests, as appropriate; significance between mock and edited samples is shown in the graphs: *$p \leq 0.05$, **$p \leq 0.01$, ***$p \leq 0.001$, and ****$p \leq 0.0001$.

CellPress
OPEN ACCESS

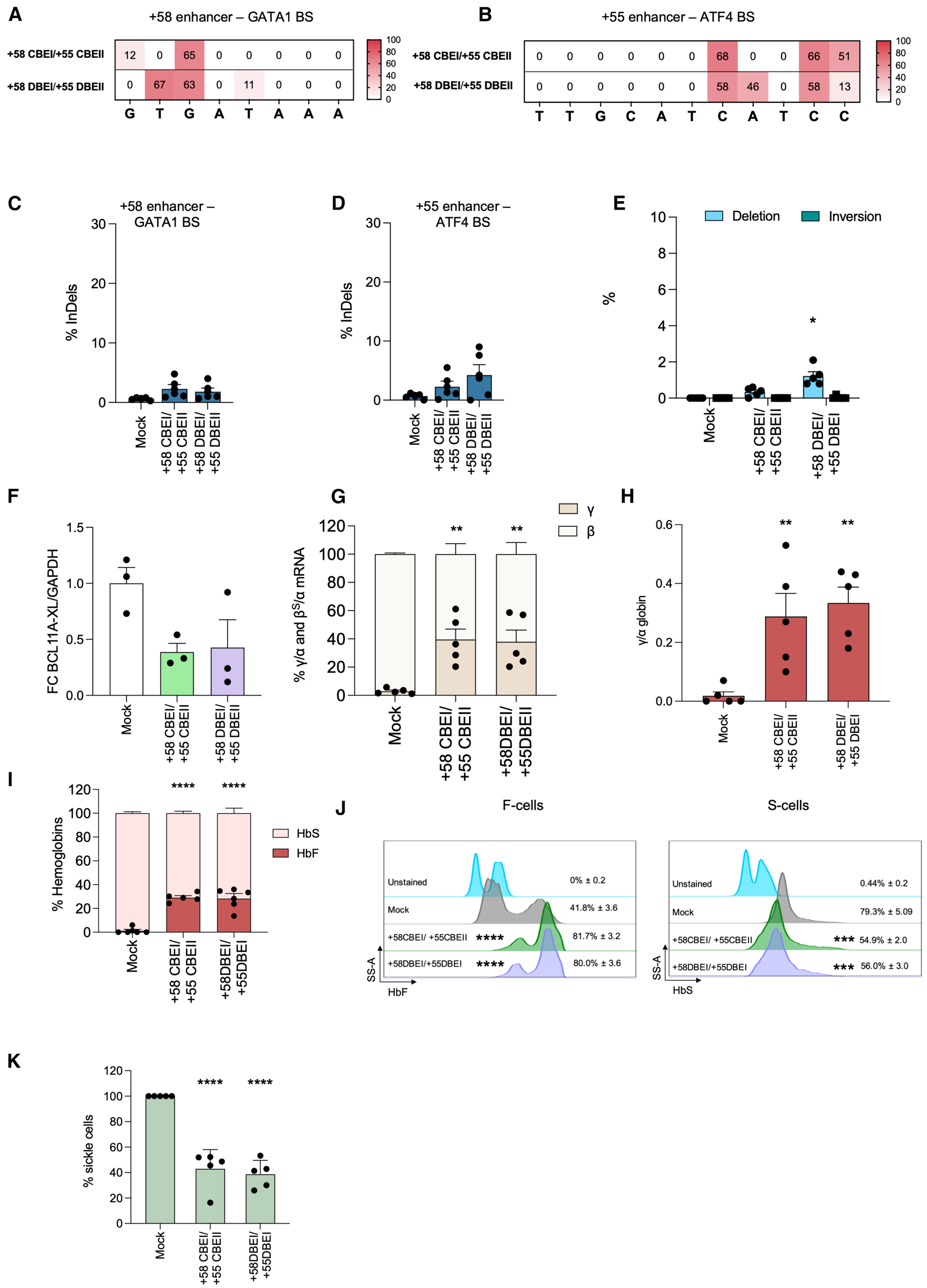

(legend on next page)

observed when using BEs compared to Cas9 nuclease (Figure 6C). For the remaining alleles, InDels associated with the generation of DSBs at the target sites were more frequent for Cas9 nuclease than BEs (Figure 6C). In addition, when using BEs, the majority of InDels were small (<50 bp) except for the +58 DBEI/+55 DBEI profile that showed 3.2% of large deletion (>200 bp) at the GATA1 BS (Figure 6C). Long-read sequencing showed a higher frequency of alleles simultaneously edited at GATA1 and ATF4 BSs when generating the +58 DBEI/+55 DBEI compared to the +58 CBEI/+55 CBEII profile (Figure 6D). To identify sgRNA-dependent potential off-targets, we used *in-silico* tools, and performed an unbiased genome-wide analysis, GUIDE-Seq, in K562 cells. Interestingly, the top ten predicted off-target sites mapped to non-coding sequences and harbored >3 mismatches (Figure 6E). NGS of the top 5 GUIDE-seq predicted off-targets and of the top 5 *in-silico* predicted off-targets (Table S1) in erythroblasts derived from edited SCD HSPCs validated few off-targets, which were limited to intronic and intergenic regions, suggesting no impact at the protein level (Figures 6F and 6G). Additionally, off-target activity did not affect the expression of targeted genes in HSPCs (Table S2). Notably, although on-target activity was similar, CBE showed higher off-target activity compared to DBE, suggesting a higher specificity of the latter enzyme (Figure 6E). Importantly, no InDels were detected at the different off-target sites (Figures 6F and 6G).

Whole-exome sequencing (WES) revealed a similar frequency of A-to-G and C-to-T mutations in the different samples, indicating that BEs do not increase the mutational burden (Figures 6H and 6I).

RNA sequencing (RNA-seq) analysis of SCD HSPCs mock-edited or edited with DBE, CBE, or Cas9 nucleases showed few or no differentially expressed genes in DBE- and CBE-treated samples, respectively (Figure 6J; Table S2; log2 fold change [log2FC] $\geq$ 1 or ≤ -1; false discovery rate [FDR] $\leq$ 0.05). On the contrary, 49 genes were upregulated in Cas9-treated samples (Figure 6J), mainly involved in p53 pathways, inflammatory response, and apoptosis. The frequency of A-to-G and C-to-T conversions in the transcriptome was comparable across all edited samples, indicating that BEs do not increase RNA deamination (Figures 6K and 6L).

Efficient multiplex base editing of *BCL11A* enhancers in repopulating hematopoietic stem cells (HSCs)

To evaluate the ability of BEs to simultaneously target the *BCL11A* enhancers in repopulating HSCs, we xenotransplanted control and CBE- or DBE-edited healthy donor (HD) HSPCs into immunodeficient NOD.Cg-KitW−-41JTyr+PrkdcscidIl2rgtm1Wjl/ThomJ (NBSGW) mice (Figure 7A). Sixteen to seventeen weeks post-transplantation, no significant differences were observed in engraftment or differentiation between edited and control HSPCs, as measured by the frequency of human CD45$^+$ cells in hematopoietic tissues and the proportion of the different lineages (Figures 7B and S8A). However, we observed a modest but non-significant decrease in the proportion of both immature (mCD45-hCD45$^+$) and mature (mCD45-hCD45$^-$) CD235$^+$ erythroid cells (Figure S8B).

The base editing efficiency in human bone marrow (BM) cells (33.7% ± 4.6/59.2% ± 13.3 for +58 CBEI/+55 CBEII, and 68.8% ± 15/63.1% ± 9.4 for +58 DBEI/+55 DBEII) was similar to the frequency observed in the input cells and the other compartments (blood, erythroid precursors, thymus, spleen, and B cells; Figures 7C–7F). However, CBE editing efficiency at the +58 enhancer tends to be reduced *in vivo* compared to the input HSPCs (Figure 7C). InDels and 3.2-kb deletions were detected at low frequencies in engrafted cells (Figures S8C and S8D). Notably, a significant reduction of 3.2-kb deletion frequency was detected *in vivo* compared to the input cells (Figure S8D).

Human CD235a$^+$ erythroid cells sorted from the BM of mice transplanted with edited cells showed increased HbF expression at the RNA and protein levels compared to control groups (Figures 7G, S8E, and S8F). Notably, higher HbF reactivation was observed in samples carrying the +58 DBEI/+55 DBEI profile, which showed the highest base editing efficiency (Figures 7G, S8E, and S8F). Correlation analysis between HbF expression and editing efficiency in BM CD235a$^+$ sorted cells also confirmed a more potent HbF reactivation associated with the +58 DBEI/+55 DBEII profile *in vivo* compared to the +58 CBEI/+55 CBEII profile (Figure S8G). Importantly, *BCL11A* down-regulation was observed in edited human BM CD235a$^+$ erythroid cells (Figure 7H), while no differences were detected between control and edited samples in BM human non-erythroid CD19$^+$ B cells (Figure 7I).

Figure 5. Multiplex CBE and DBE base editing in SCD HPSC-derived erythroblasts

(A and B) C-G to T-A or/and A-T to G-C base-editing efficiency in the +58-kb (A) or +55-kb (B) in erythroblasts derived from edited SCD HSPCs. GATA_bs_1 and ATF4_bs_2 were used in combination with AncBE4max to generate the +58 CBE/+55 CBEII profile and in combination with TadDE to generate the +58 DBEI/+55 DBEI profile.

(C and D) Frequency of InDels in the +58-kb (C) or +55-kb (D).

(E) Frequency of the 3.2-kb deletion/inversion, measured by ddPCR, in erythroblasts derived from edited SCD HSPCs. *$p \leq 0.05$. One-way ANOVA with Dunnett's correction for multiple comparison. Comparison of controls vs. edited samples.

(F) ddPCR analysis of *BCL11A* mRNA in erythroblasts derived from edited SCD HSPCs at day 13 of erythroid differentiation.

(G) RT-qPCR analysis of γ ($^G\gamma$ + $^A\gamma$)- and β^S-globin mRNA in control and edited SCD erythroblasts at day 13 of erythroid differentiation.

(H) Expression of γ ($^G\gamma$ + $^A\gamma$)-globin chains measured by RP-HPLC in RBCs derived from SCD HSPCs.

(I) Analysis of HbF and HbS by cation-exchange HPLC in RBCs derived from edited SCD HSPCs.

(J) Representative flow cytometry histograms showing the percentage of HbF-expressing cells (F-cells) and HbS-expressing cells (S-cells) in the CD235a$^+$ population for unstained (CD235a stained only), control, and edited samples.

(K) Frequency of sickling cells upon O_2 deprivation in mock and edited samples. ****$p \leq 0.0001$. One-way ANOVA with Dunnett's correction for multiple comparisons. Statistical significance between mock and edited samples is depicted in the graph.

Data are expressed as mean (n = 1 biologically independent experiment, 5 donors). Statistical comparisons (mock vs. edited samples) were performed using one-way or two-way ANOVA with Dunnett's correction. Significance: *$p \leq 0.05$, **$p \leq 0.01$, ***$p \leq 0.001$, and ****$p \leq 0.0001$.

CellPress
OPEN ACCESS

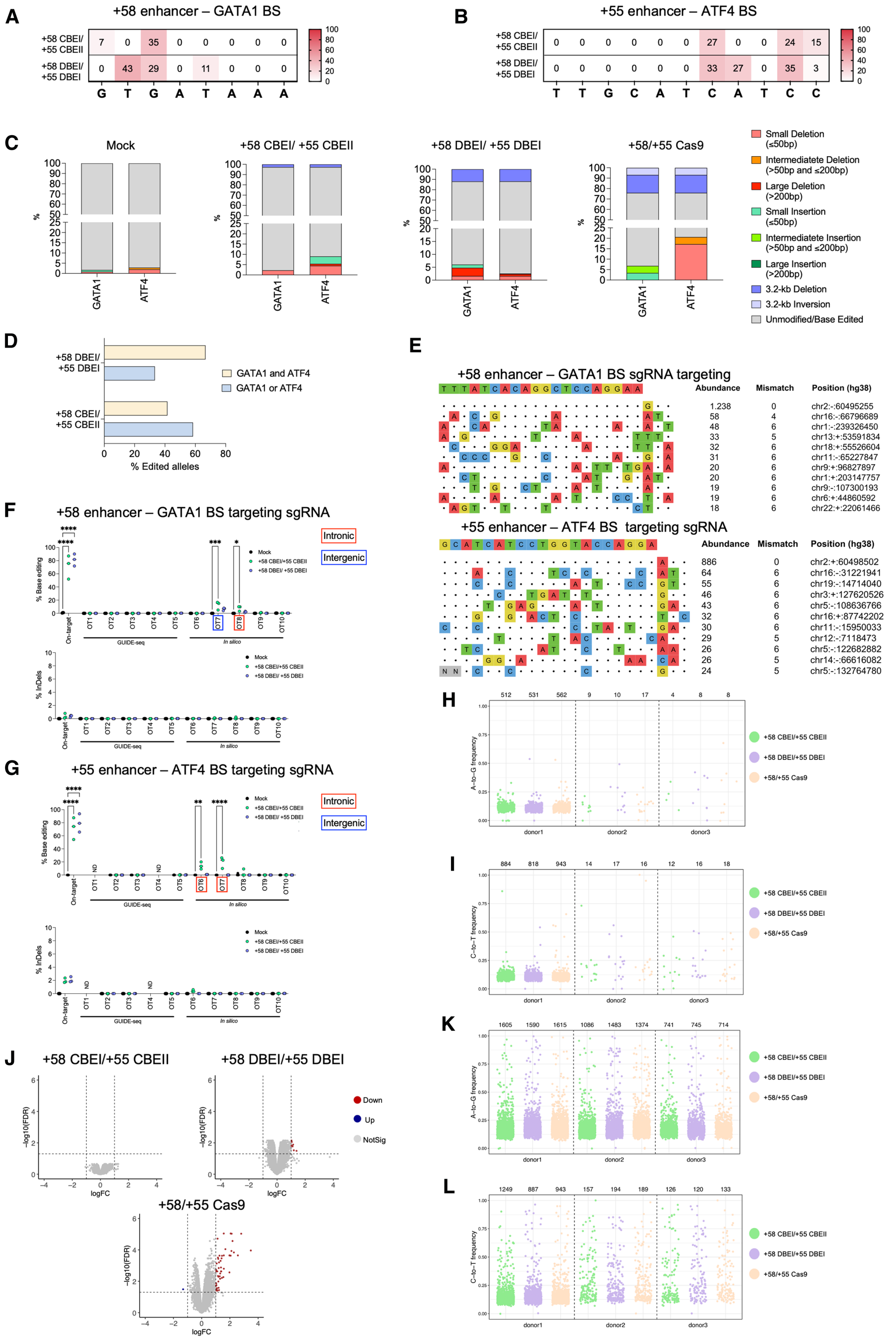

(legend on next page)

Human CD45+ BM cells were isolated and subjected to a CFC assay. Control and edited samples showed a similar number of erythroid and granulo-monocytic colonies, demonstrating no impact of base editing on the clonogenic potential of engrafted human cells (Figure 7J). Editing efficiency in BFU-E and CFU-GM was similar to that measured in the input populations except for the GATA1 BS in the +58-kb enhancer in CBE-treated samples, as observed in human CD45+ BM cells (Figures S8H–S8K). Furthermore, InDels and 3.2-kb deletions were detected at low frequencies (Figures S8L and S8M). *BCL11A-XL* down-regulation was observed in BFU-E (Figure S8N), resulting also in globin reactivation at RNA and protein levels (Figures S8O–S8Q).

Overall, these results suggest high efficiency and a good safety profile of our multiplex base editing approach in HSCs and their differentiated progeny.

DISCUSSION

Since the discovery of elements controlling the expression of HbF, a variety of genome editing strategies based on the disruption of *cis*- or *trans*-regulatory elements of *HBG1/2* to induce HbF reactivation have been developed for the treatment of β-hemoglobinopathies.[7,8,11,19]

Cas9 nuclease-mediated disruption of the +58-kb erythroid-specific *BCL11A* enhancer has been recently approved as a clinical therapy for patients with SCD and β-thalassemia.[9,10] Although significant HbF reactivation was achieved, the clinical study showed variability between the patients in HbF levels. Furthermore, SCD patients retained high HbS levels and showed a modest correction of ineffective erythropoiesis. Thus, further optimization of the strategy is advisable to reach the complete rescue of the phenotype by reaching higher levels of HbF. In addition, the generation of Cas9 nuclease-induced DSBs or large genomic rearrangements can be detrimental to HSCs.[12,24–27] It has been recently proposed to use the base editing technology to disrupt the GATA1 activator BS in the erythroid-specific *BCL11A* enhancer without creating InDels, and reactivate HbF in the progeny of SCD and β-thalassemia patient HSPCs.[16,18,28]

We used SCD HSPCs to perform a screening of different BE/sgRNAs to disrupt the GATA1 and ATF4 activator BS in the erythroid-specific *BCL11A* enhancer regions (+58-kb and +55-kb, respectively) by changing specific targeted nucleotides. Indeed, modifying the GATA1 BS differentially reduces the ability of GATA1 to bind to the *BCL11A* enhancer, and thus modulate gene expression.[21] We were able to create different editing profiles for both motifs by combining different sgRNAs and ABEs or CBEs to generate A>G or C>T base conversions. Additionally, the use of TadDE allowed us to extend the number of edited bases and further disrupt the GATA1 and ATF4 BSs.

When targeting individually the GATA1 and ATF4 BSs, only the generation of the +58 CBEI profile at the GATA1 BS led to HbF expression and consequent rescue of the sickle phenotype, similar to the approved Cas9 nuclease-based approach. To further increase HbF levels, we hypothesized that combined editing of the +58-kb and +55-kb enhancers could lead to a stronger reduction of *BCL11A* expression, and consequently greater HbF induction compared to editing a single enhancer alone. Multiplex targeting of the +58-kb and +55-kb *BCL11A* enhancers using CRISPR-Cas9 (although targeting different binding site locus in the +55-kb enhancer) was recently reported to efficiently reactivate HbF expression.[29,30] However, the use of CRISPR-Cas9 nucleases causes potentially dangerous DSBs and large genomic rearrangements.

Correlation analysis on single cell-derived colonies allowed us to identify the critical bases involved in the binding of GATA1 and

Figure 6. Evaluation of on-target aberrations and off-target activity of multiplex base editing of +58-kb and +55-kb regions

(A and B) C-G to T-A or/and A-T to G-C base-editing efficiency in the +58-kb (A) and +55-kb (B) target region obtained with the generation of +58 CBEI/+55 CBEII profile and +58 DBEI/+55 DBEII.

(C) Comprehensive allele frequencies of diverse types of genomic rearrangements and InDel types in SCD HSPC-derived erythroblasts. We reported frequencies of events observed at individual GATA1 and ATF4 BSs (in the absence of the 3.2-kb deletion/inversion) and the frequencies of 3.2-kb deletion/inversion (GATA1-ATF4). InDels were classified as small, intermediate, and large according to their size ([4–50 bp], [51–200 bp], and >200 bp). Del, deletion; Ins, insertion; Inv, inversion. Unmodified, no deletion, inversion, or insertion observed.

(D) Frequency of alleles edited at the GATA1 and ATF4 BSs, either simultaneously (GATA1 and ATF4) or at only one of the sites (GATA1 or ATF4). The reported data were adjusted for background error typical of this analysis by subtracting the frequency of edited alleles in the mock condition from the fraction of edited alleles in the edited conditions.

(E) sgRNA-dependent off-target sites of the GATA_bs_1 (top) and ATF4_bs_2 (bottom) sgRNAs in K562 cells, as evaluated by GUIDE-seq analysis. The protospacer targeted by each sgRNA is reported on top of each panel, followed by the off-target sites and their mismatches with the on-target (highlighted in color). The number of sequencing reads (abundance), the number of mismatches, and the chromosomal coordinates (hg38) of each off-target site are reported.

(F and G). Base editing and InDel frequency at on-target and off-target (OT) sites, in SCD HSPC-derived erythroblasts, were evaluated for GATA1 BS targeting sgRNA (F) and ATF4 BS targeting sgRNA (G) for control, +58 CBEI/+ 55 CBEII and +58 DBEI/+55 DBEI profiles as measured by targeted NGS. OT sites in introns or intergenic regions for which we detected editing are highlighted by a red or blue square shape, respectively. Data are expressed as individual values and median (n = 3 biologically independent experiments, 3 donors) $^{*}p \leq 0.05$, $^{**}p \leq 0.01$, $^{***}p \leq 0.001$, and $^{****}p \leq 0.0001$. Two-way ANOVA with Dunnett's statistical significance between mock and edited samples is depicted in the graph.

(H and I) Strip plots showing the variant allele frequency (A > G, H; C > T, I) in exons, observed in SCD HSPCs obtained from three different donors and measured by WES. The total number of variants are indicated above each sample.

(J) Volcano plots showing differential gene expression between cells treated with CBE, DBE, or Cas9 nuclease and mock-treated samples. The horizontal dashed line indicates the threshold on the false discovery rate (FDR $\leq$ 0.05), and the vertical dashed lines correspond to the threshold on log2FC ≥ 1 or ≤ -1. Upregulated genes are indicated in red, and downregulated genes are in blue. Genes in gray are not differentially expressed. (n = 1 biologically independent experiments, 3 donors).

(K and L) Strip plots showing the variant allele frequency (A > G, K; C > T, L) in the transcriptome observed in SCD HSPCs obtained from three different donors and measured by RNA-seq. The total number of variants are indicated above each sample.

CellPress
OPEN ACCESS

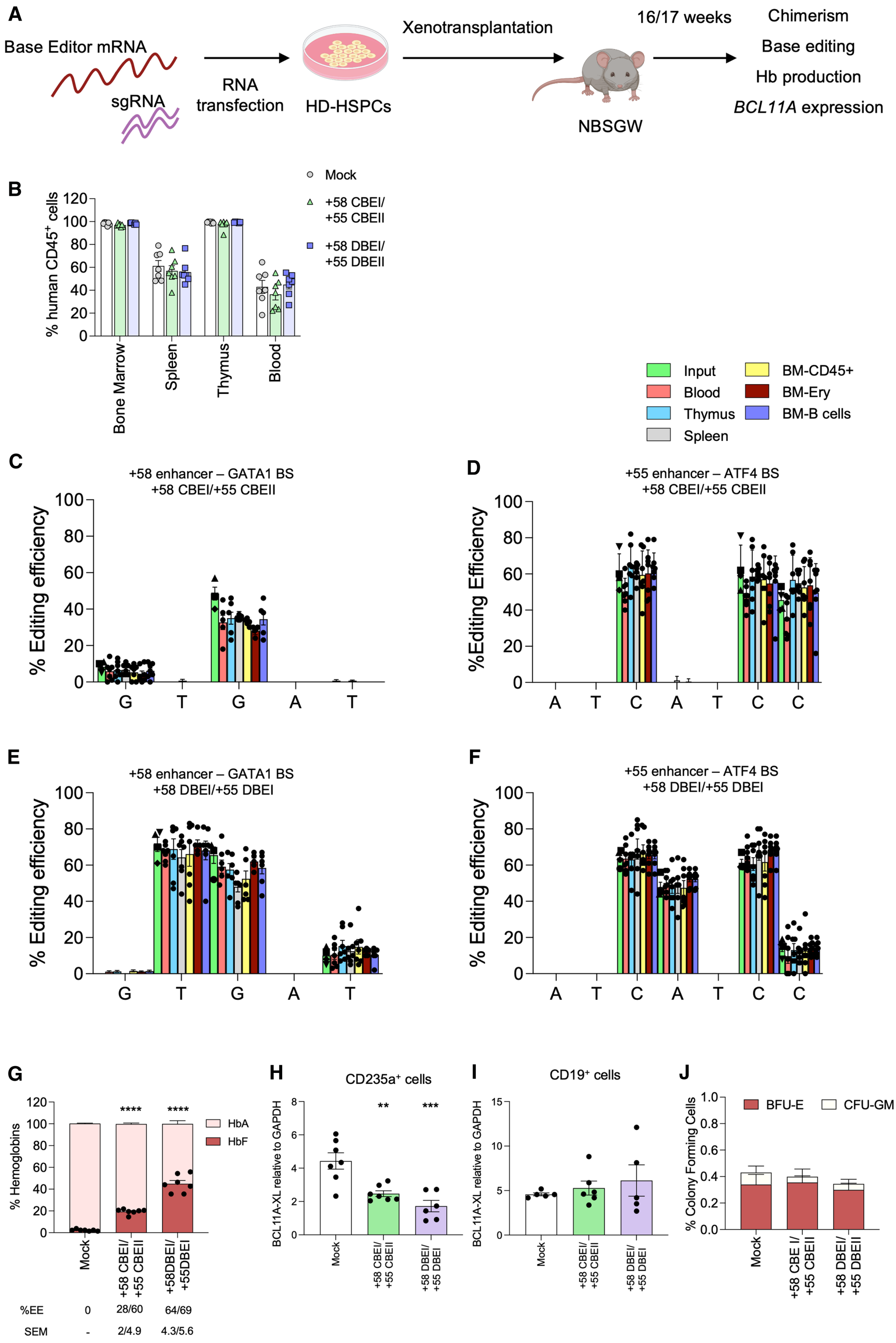

(legend on next page)

ATF4 at the *BCL11A* enhancers and the most potent profiles in terms of HbF reactivation that we can combine to further improve the pathological phenotype.

Regarding the +58-kb region, our findings corroborate previous studies that reported an important role of the G_3 of the GATA binding motif in regulating GATA1 binding.[21] This is supported by the elevated γ-globin levels observed upon generating the +58 CBEI profile (GTGATAAA), effectively replicating the outcomes achieved with the approved Cas9 nuclease strategy. In addition, the A_6 position in the GATA1 BS (GTGATAAA; +58 ABEIII profile) plays an important role in GATA1 binding, as already observed in other works[21] and confirmed by the strong increase of γ-globin reactivation. In contrast, targeting positions A_6 to A_8 in the GATA1 binding motif (GTGATAAA) to generate the +58 ABEII profile resulted in reduced γ-globin expression compared with the +58 ABEIII profile. This reduction suggests that the A>G conversion at the positions A_7 and A_8, in addition to the A_6 base conversion, might restore a binding motif for transcriptional activators, which affects *BCL11A* and, therefore, γ-globin expression.

Similarly, T>C conversions generated at position T_2 and T_5 (GTGATAAA) to create the +58 ABEI profile led to less potent γ-globin re-activation and presumably less efficient disruption of GATA1 BS compared to the generation of the +58 ABEIII profile, although previous studies reported high GATA1 displacement when T>C mutation was occurring at position 5 of the GATA binding motif compared to the targeting of A_6.[21] These results suggest that site-specific requirements for GATA1 binding at the +58-kb enhancer, likely because of the surrounding sequence, could also influence GATA1 occupancy.

Regarding the +55-kb region, the generation of the +55 CBEI profile led to C>T conversion of all the Cs (C_4, C_7, C_{10}, and C_{11}) of the ATF4 binding motif (TTGCATCATCC), while with the generation of +55 CBEII profile only C_7, C_{10}, and C_{11} were converted (TTGCATCATCC). Our data suggest that the insertion of two points mutations at C_4 and C_7 (+55 CBEI profile) does not further displace ATF4 as compared to the generation of C_7 mutation alone (+55 CBEII profile), indicating that the primary factor affecting ATF4 binding (and subsequently *BCL11A* expression and γ-globin reactivation), is the C>T conversion at C_7. Conversions of the A_5 and A_8 of the ATF4 binding motif (TTGCATCATCC, +55 ABEI and TTGCATCATCC, +55 ABEII) led to low *HBG* levels, suggesting a modest impact on ATF4 binding. However, the generation of the +55 ABEII profile (TTGCATCATCC) showed a higher effect in terms of γ-globin reactivation compared to the +55 ABEI profile (TTGCATCATCC). This result can either indicate a stronger impact of the A_8 mutation in the displacement of ATF4 or that the simultaneous generation of A_5 and A_8 mutations enhanced the disruption of the ATF4 binding motif.

Finally, we used DBE to further disrupt the GATA1 and ATF4 BSs; however, *in vitro,* we observed only a modest increase in HbF reactivation compared to the use of CBE alone, suggesting a minor role of As in the binding of these factors to the *BCL11A* enhancers.

This study identifies critical bases within the BS motif and allowed precise modulation of HbF expression, with the goal of enhancing the therapeutic efficacy in patients with SCD while minimizing potential adverse effects associated with excessive HbF levels. Importantly, HbF reactivation obtained upon multiplex base editing reached the levels needed to ameliorate the clinical manifestations of SCD, which have been defined as 70% of HbF-expressing cells and HbF accounting for 30% of the total hemoglobin.[31] While other therapeutic strategies directly targeting the *HBG* promoters can achieve even higher levels of HbF,[16,20,32] such supraphysiological expression may compromise tissue oxygenation due to the higher oxygen affinity of HbF.[33] In contrast, our approach yields a robust yet balanced HbF reactivation, likely minimizing the risk of impaired oxygen delivery. Comparison of strategies targeting these alternative *cis*-regulatory regions in patients will allow the full assessment of their safety and efficacy.

Fewer genomic rearrangements were detected in base-edited samples compared to cells treated with Cas9 nuclease, highlighting the better safety profile of our approach. We showed that large genomic rearrangements (particularly deletions) within the 3.2-kb region between the GATA1 and ATF4 occur more frequently with DBE compared to the CBE, while ABE showed no such large deletions. While higher rearrangements with DBE can be explained simply by the additive effect of CBE and ABE activity, the reduced deletion frequency observed with ABE compared to CBE is probably due to an inefficient excision of inosines (in the case of ABEs) compared to the excision of the uracil base (in the case of CBEs), which reduces the generation of DSBs. In addition, the C>G base conversions that were observed with CBE and DBE have been previously associated with DSB generation, deletions, and other large genomic rearrangements.[34] As for the 3.2-kb deletion, also on-target unintended large deletions (>200 bp) were more frequent in DBE-treated samples (+58 DBEI/+55 DBEI profile) compared to CBE-treated samples (+58 CBEI/+55 CBEII profile); however,

Figure 7. Simultaneous base editing of *BCL11A* enhancers in repopulating HSCs

(A) Xenotransplantation protocol: BE mRNAs and sgRNAs were co-transfected into HD HSPCs, followed by transplantation into NBSGW mice 1 day post-transfection. Mice were euthanized 16–17 weeks later for hematopoietic tissue analysis.

(B) Engraftment of human cells with control (mock-transfected) or edited HSPCs ($n = 7$ mice per group). Engraftment is represented as the percentage of human $CD45^+$ cells in the total murine and human $CD45^+$ cell population in BM, spleen, thymus, and peripheral blood. Each data point represents an individual mouse.

(C–F) C-G to T-A or/and A-T to G-C base-editing efficiency for +58 CBEI/+55 CBEII (C and D) and +58 DBEI/+55 DBEI (E and F) profiles at the +58-kb (C and E) and +55-kb (D and F) regions in the input, blood-, bone marrow (BM)- ($CD45^+$, B, and erythroid cells), thymus-, and spleen-derived human samples. Each data point represents an individual mouse. Input includes cells cultured in the HSPC medium (■), in liquid erythroid cultures (▲), and pools of BFU-E (◆) and CFU-GM (▼).

(G) Analysis of HbF and HbS by cation-exchange HPLC in human $CD235a^+$ BM-sorted erythroid cells from transplanted mice.

(H and I) RT-qPCR analysis of *BCL11A* in BM-sorted $CD235a^+$ (H) or $CD19^+$ (I) cells from transplanted mice. Each data point represents an individual mouse.

(J) Human hematopoietic progenitor content (% BFU-E and CFU-GM) among BM-derived human $CD45^+$ cells from transplanted mice.

Data are expressed as mean ± SEM ($n = 1$ for input; n = 5–7 mice per group). Statistical analysis was performed using one-way or two-way ANOVA with Dunnett's or Tukey's correction, as appropriate; significance between control and edited groups is indicated in the graphs: $^*p \leq 0.01$, $^{**}p \leq 0.001$, and $^{***}p \leq 0.0001$.

deletion frequency remained lower compared to the samples treated with the Cas9 nuclease.

Targeted deep sequencing of the top 5 GUIDE-seq and *in-silico* predicted off-targets in base-edited HSPCs showed a low sgRNA-dependent off-target activity, occurring in non-exonic regions, with no predicted effect at the protein level, and no consequences at the RNA level, as confirmed by RNA-seq. In addition, a comparison between DBE and CBE revealed a higher specificity of DBE. Importantly, no InDel were detected at the off-target sites, suggesting that BEs minimize the possibility of generating DSB-induced genomic rearrangements such as translocations. In addition, we comprehensively assessed the sgRNA-independent off-target DNA activity by WES, demonstrating no sgRNA-independent off-target activity within exons in DBE-and CBE-treated samples. Finally, we analyzed sgRNA-independent off-target RNA activity, showing that the proposed base editing strategies did not lead to deamination of the cellular transcriptome.

Xenotransplantation experiments of multiplex base-edited HSPCs in immunodeficient mice confirmed high editing efficiencies in long-term repopulating HSCs and their progeny, demonstrating that multiplex base editing does not affect engraftment and multilineage differentiation of HSCs. Of note, we achieved higher editing frequency and HbF reactivation with DBE compared to CBE *in vivo*. Correlation analysis between editing efficiency and HbF levels suggested that DBE-induced multiple mutations in the *BCL11A* enhancers better evict GATA1 and ATF4 and downregulate *BCL11A in vivo*. These results also suggest that CBEs might be less efficient in *bona fide* HSCs. In addition, CBEs might have induced some toxicity *in vivo*, which could have affected the cell viability and self-renewal capacity of HSCs, as previously proposed.[19]

Of note, complete inactivation of *BCL11A* adversely affects lymphoid development and vital HSC functions.[35–37] However, *in vivo* experiments confirmed previous observations that the downregulation of *BCL11A* achieved by targeting the GATA1 BS in the +58-kb erythroid-specific enhancer does not impair *BCL11A* expression in other lineages.[38] In addition, here we demonstrated that the targeting of ATF4 BS in the +55-kb enhancer also led to specific downregulation of *BCL11A* in the erythroid lineage without impacting its expression in B cells. Nonetheless, knocking out *BCL11A* can also harm the erythroid lineage by affecting the human RBC enucleation and inducing ineffective erythropoiesis.[39–41] Our study also showed some alterations in cell viability and growth, and enucleation *in vitro* and erythroid cell production *in vivo*. However, these changes were modest, likely because of the partial down-regulation of *BCL11A* expression achieved with our base editing strategy. These findings are in line with previous studies showing that patients with BCL11A haploinsufficiency have normal hematological parameters.[42,43] Furthermore, a recent *in vivo* study in non-human primates[29] reported preserved erythropoiesis following CRISPR-Cas9-mediated *BCL11A* downregulation.

In conclusion, this study provides insights into the applicability of multiplexed base editing strategies to treat SCD and potentially β-thalassemia patients through disruption of the erythroid-specific *BCL11A* enhancers in the +58-kb and +55-kb regions. It is worth noting that our approaches can serve as universal therapeutic strategies for both SCD and β-thalassemia patients, as they do not require the design of mutation-specific CRISPR-Cas9-based tools, as proposed in previous studies.[18,44,45] The levels of HbF achieved with multiplex base editing were sufficient to ameliorate the sickling phenotype.

Limitations of the study

The clinical translation of our approach will require the establishment of a large-scale transfection protocol with clinical-grade reagents, biodistribution studies in mice, and an extensive evaluation of the off-target activity of our base editing systems, i.e., the analysis of a larger panel of *in-silico* predicted sites (e.g., >300 potential off-targets).[46]

RESOURCE AVAILABILITY

Lead contact

Requests for further information and resources should be directed to and will be fulfilled by the lead contact, Miccio Annarita (annarita.miccio@institutimagine.org).

Materials availability

All plasmids generated in this study are available from the lead contact with a completed materials transfer agreement.

Data and code availability

- RNA-seq data have been deposited in the Gene Expression Omnibus repository and are publicly available as of the date of publication. Accession numbers are listed in the key resources table.
- Fastq files generated by WES and the raw long-read sequencing and GUIDE-seq data have been deposited in the SRA database (https://www.ncbi.nlm.nih.gov/sra) and are publicly available as of the date of publication. Accession numbers are listed in the key resources table.
- The original code has been deposited at GitHub and is publicly available at Zenodo as of the date of publication.
- Any additional information required to reanalyze the data reported in this work paper is available from the lead contact upon request.

ACKNOWLEDGMENTS

This work was supported by state funding from the French National Research Agency as part of the Investissements d'Avenir program (ANR-10-IAHU-01) and by the European Research Council (865797 DITSB), the European Commission (HORIZON-RIA EDITSCD, grant no. 101057659), the Fondation pour la Recherche Médicale (FRM PLP202110014595) and the COST (European Cooperation in Science and Technology (the COST Action Gene Editing for the treatment of Human Diseases, CA21113).

We thank Carine Giovannangeli for the production of Cas9 nuclease RNP and the patients for their contribution to this work.

AUTHOR CONTRIBUTIONS

L.F. designed, conducted experiments, analyzed the data, and wrote the paper. P.M., S.A., T.F., M.M., G.C., A.T., A.C., G.H., and J.M. conducted experiments and analyzed data. O.R. analyzed NGS data. M.A. contributed to the design of the experimental strategy. P.A. conceived the study, designed, conducted experiments, analyzed the data, and wrote the paper. A.M. conceived the study, designed experiments, and wrote the paper.

DECLARATION OF INTERESTS

P.A. and A.M. are the inventors of a patent describing base editing approaches for hemoglobinopathies (PCT/EP2022/083904: Methods for increasing HbF content by editing the +55-kb region of the erythroid-specific bcl11a enhancer).

STAR★METHODS

Detailed methods are provided in the online version of this paper and include the following:

- KEY RESOURCES TABLE
- EXPERIMENTAL MODEL AND STUDY PARTICIPANT DETAILS
- METHOD DETAILS
  - Base editor-expressing plasmids
  - Single guide RNA design and production
  - mRNA *in vitro* transcription
  - RNA transfection
  - Ribonucleoprotein (RNP) transfection
  - Colony-forming cell assay
  - Genome-wide, unbiased identification of double-strand breaks enabled by sequencing (GUIDE-seq)
  - Evaluation of editing efficiency
  - RT-qPCR
  - Flow cytometry analysis
  - Reverse phase high-performance liquid chromatography analysis of globin chains
  - CE-HPLC analysis of hemoglobin tetramers
  - Digital droplet PCR
  - Sickling assay
  - Single guide RNA design for Cas9-enrichment library preparation
  - Cas9-enrichment library preparation for nanopore sequencing
  - Bioinformatic analysis of Cas9-enrichment library from nanopore sequencing
  - RNA-seq
  - Detection of RNA editing events by RNA-seq
  - Whole-exome sequencing
  - Hematopoietic stem/progenitor cell xenotransplantation in NOD.Cg-KitW−-41JTyr+PrkdcscidIl2rgtm1Wjl/ThomJ mice
- QUANTIFICATION AND STATISTICAL ANALYSIS

SUPPLEMENTAL INFORMATION

Supplemental information can be found online at https://doi.org/10.1016/j.xcrm.2025.102376.

Received: May 15, 2025
Revised: July 8, 2025
Accepted: September 2, 2025
Published: September 26, 2025

REFERENCES

1. Kato, G.J., Piel, F.B., Reid, C.D., Gaston, M.H., Ohene-Frempong, K., Krishnamurti, L., Smith, W.R., Panepinto, J.A., Weatherall, D.J., Costa, F.F., and Vichinsky, E.P. (2018). Sickle cell disease. Nat. Rev. Dis. Primers *4*, 18010. https://doi.org/10.1038/nrdp.2018.10.

2. Fernandes, Q. (2017). Therapeutic strategies in Sickle Cell Anemia: The past present and future. Life Sci. *178*, 100–108. https://doi.org/10.1016/j.lfs.2017.03.025.

3. Li, L., and Mandal, P.K. (2024). Recent advancements in gene therapy for sickle cell disease and β-thalassemia. Front. Hematol. *3*, 1468952. https://doi.org/10.3389/frhem.2024.1468952.

4. Steinberg, M.H., Chui, D.H.K., Dover, G.J., Sebastiani, P., and Alsultan, A. (2014). Fetal hemoglobin in sickle cell anemia: a glass half full? Blood *123*, 481–485. https://doi.org/10.1182/blood-2013-09-528067.

5. Traxler, E.A., Yao, Y., Wang, Y.-D., Woodard, K.J., Kurita, R., Nakamura, Y., Hughes, J.R., Hardison, R.C., Blobel, G.A., Li, C., and Weiss, M.J. (2016). A genome-editing strategy to treat β-hemoglobinopathies that recapitulates a mutation associated with a benign genetic condition. Nat. Med. *22*, 987–990. https://doi.org/10.1038/nm.4170.

6. Métais, J.-Y., Doerfler, P.A., Mayuranathan, T., Bauer, D.E., Fowler, S.C., Hsieh, M.M., Katta, V., Keriwala, S., Lazzarotto, C.R., Luk, K., et al. (2019). Genome editing of HBG1 and HBG2 to induce fetal hemoglobin. Blood Adv. *3*, 3379–3392. https://doi.org/10.1182/bloodadvances.2019000820.

7. Weber, L., Frati, G., Felix, T., Hardouin, G., Casini, A., Wollenschlaeger, C., Meneghini, V., Masson, C., De Cian, A., Chalumeau, A., et al. (2020). Editing a γ-globin repressor binding site restores fetal hemoglobin synthesis and corrects the sickle cell disease phenotype. Sci. Adv. *6*, eaay9392. https://doi.org/10.1126/sciadv.aay9392.

8. Wu, Y., Zeng, J., Roscoe, B.P., Liu, P., Yao, Q., Lazzarotto, C.R., Clement, K., Cole, M.A., Luk, K., Baricordi, C., et al. (2019). Highly efficient therapeutic gene editing of human hematopoietic stem cells. Nat. Med. *25*, 776–783. https://doi.org/10.1038/s41591-019-0401-y.

9. Frangoul, H., Locatelli, F., Sharma, A., Bhatia, M., Mapara, M., Molinari, L., Wall, D., Liem, R.I., Telfer, P., Shah, A.J., et al. (2024). Exagamglogene Autotemcel for Severe Sickle Cell Disease. N. Engl. J. Med. *390*, 1649–1662. https://doi.org/10.1056/NEJMoa2309676.

10. Locatelli, F., Lang, P., Wall, D., Meisel, R., Corbacioglu, S., Li, A.M., De La Fuente, J., Shah, A.J., Carpenter, B., Kwiatkowski, J.L., et al. (2024). Exagamglogene Autotemcel for Transfusion-Dependent β-Thalassemia. N. Engl. J. Med. *390*, 1663–1676. https://doi.org/10.1056/NEJMoa2309673.

11. Huang, P., Peslak, S.A., Lan, X., Khandros, E., Yano, J.A., Sharma, M., Keller, C.A., Giardine, B., Qin, K., Abdulmalik, O., et al. (2020). The HRI-regulated transcription factor ATF4 activates BCL11A transcription to silence fetal hemoglobin expression. Blood *135*, 2121–2132. https://doi.org/10.1182/blood.2020005301.

12. Cullot, G., Boutin, J., Toutain, J., Prat, F., Pennamen, P., Rooryck, C., Teichmann, M., Rousseau, E., Lamrissi-Garcia, I., Guyonnet-Duperat, V., et al. (2019). CRISPR-Cas9 genome editing induces megabase-scale chromosomal truncations. Nat. Commun. *10*, 1136. https://doi.org/10.1038/s41467-019-09006-2.

13. Fiumara, M., Ferrari, S., Omer-Javed, A., Beretta, S., Albano, L., Canarutto, D., Varesi, A., Gaddoni, C., Brombin, C., Cugnata, F., et al. (2024). Genotoxic effects of base and prime editing in human hematopoietic stem cells. Nat. Biotechnol. *42*, 877–891. https://doi.org/10.1038/s41587-023-01915-4.

14. Richter, M.F., Zhao, K.T., Eton, E., Lapinaite, A., Newby, G.A., Thuronyi, B. W., Wilson, C., Koblan, L.W., Zeng, J., Bauer, D.E., et al. (2020). Phage-assisted evolution of an adenine base editor with improved Cas domain compatibility and activity. Nat. Biotechnol. *38*, 883–891. https://doi.org/10.1038/s41587-020-0453-z.

15. Banskota, S., Raguram, A., Suh, S., Du, S.W., Davis, J.R., Choi, E.H., Wang, X., Nielsen, S.C., Newby, G.A., Randolph, P.B., et al. (2022). Engineered virus-like particles for efficient in vivo delivery of therapeutic proteins. Cell *185*, 250–265. https://doi.org/10.1016/j.cell.2021.12.021.

16. Liao, J., Chen, S., Hsiao, S., Jiang, Y., Yang, Y., Zhang, Y., Wang, X., Lai, Y., Bauer, D.E., and Wu, Y. (2023). Therapeutic adenine base editing of human hematopoietic stem cells. Nat. Commun. *14*, 207. https://doi.org/10.1038/s41467-022-35508-7.

17. Neugebauer, M.E., Hsu, A., Arbab, M., Krasnow, N.A., McElroy, A.N., Pandey, S., Doman, J.L., Huang, T.P., Raguram, A., Banskota, S., et al. (2023). Evolution of an adenine base editor into a small, efficient cytosine base editor with low off-target activity. Nat. Biotechnol. *41*, 673–685. https://doi.org/10.1038/s41587-022-01533-6.

18. Zeng, J., Wu, Y., Ren, C., Bonanno, J., Shen, A.H., Shea, D., Gehrke, J.M., Clement, K., Luk, K., Yao, Q., et al. (2020). Therapeutic base editing of human hematopoietic stem cells. Nat. Med. *26*, 535–541. https://doi.org/10.1038/s41591-020-0790-y.

19. Antoniou, P., Hardouin, G., Martinucci, P., Frati, G., Felix, T., Chalumeau, A., Fontana, L., Martin, J., Masson, C., Brusson, M., et al. (2022). Base-editing-mediated dissection of a γ-globin cis-regulatory element for the therapeutic reactivation of fetal hemoglobin expression. Nat. Commun. *13*, 6618. https://doi.org/10.1038/s41467-022-34493-1.

20. Mayuranathan, T., Newby, G.A., Feng, R., Yao, Y., Mayberry, K.D., Lazzarotto, C.R., Li, Y., Levine, R.M., Nimmagadda, N., Dempsey, E., et al. (2023). Potent and uniform fetal hemoglobin induction via base editing. Nat. Genet. *55*, 1210–1220. https://doi.org/10.1038/s41588-023-01434-7.

21. Behera, V., Evans, P., Face, C.J., Hamagami, N., Sankaranarayanan, L., Keller, C.A., Giardine, B., Tan, K., Hardison, R.C., Shi, J., and Blobel, G. A. (2018). Exploiting genetic variation to uncover rules of transcription factor binding and chromatin accessibility. Nat. Commun. *9*, 782. https://doi.org/10.1038/s41467-018-03082-6.

22. Koblan, L.W., Doman, J.L., Wilson, C., Levy, J.M., Tay, T., Newby, G.A., Maianti, J.P., Raguram, A., and Liu, D.R. (2018). Improving cytidine and adenine base editors by expression optimization and ancestral reconstruction. Nat. Biotechnol. *36*, 843–846. https://doi.org/10.1038/nbt.4172.

23. Huang, T.P., Heins, Z.J., Miller, S.M., Wong, B.G., Balivada, P.A., Wang, T., Khalil, A.S., and Liu, D.R. (2023). High-throughput continuous evolution of compact Cas9 variants targeting single-nucleotide-pyrimidine PAMs. Nat. Biotechnol. *41*, 96–107. https://doi.org/10.1038/s41587-022-01410-2.

24. Kosicki, M., Tomberg, K., and Bradley, A. (2018). Repair of double-strand breaks induced by CRISPR–Cas9 leads to large deletions and complex rearrangements. Nat. Biotechnol. *36*, 765–771. https://doi.org/10.1038/nbt.4192.

25. Schiroli, G., Conti, A., Ferrari, S., Della Volpe, L., Jacob, A., Albano, L., Beretta, S., Calabria, A., Vavassori, V., Gasparini, P., et al. (2019). Precise Gene Editing Preserves Hematopoietic Stem Cell Function following Transient p53-Mediated DNA Damage Response. Cell Stem Cell *24*, 551–565. https://doi.org/10.1016/j.stem.2019.02.019.

26. Blattner, G., Cavazza, A., Thrasher, A.J., and Turchiano, G. (2020). Gene Editing and Genotoxicity: Targeting the Off-Targets. Front. Genome *2*, 613252. https://doi.org/10.3389/fgeed.2020.613252.

27. Leibowitz, M.L., Papathanasiou, S., Doerfler, P.A., Blaine, L.J., Sun, L., Yao, Y., Zhang, C.-Z., Weiss, M.J., and Pellman, D. (2021). Chromothripsis as an on-target consequence of CRISPR–Cas9 genome editing. Nat. Genet. *53*, 895–905. https://doi.org/10.1038/s41588-021-00838-7.

28. Li, C., Georgakopoulou, A., Mishra, A., Gil, S., Hawkins, R.D., Yannaki, E., and Lieber, A. (2021). In vivo HSPC gene therapy with base editors allows for efficient reactivation of fetal γ-globin in β-YAC mice. Blood Adv. *5*, 1122–1135. https://doi.org/10.1182/bloodadvances.2020003702.

29. Demirci, S., Zeng, J., Palchaudhuri, R., Wu, C., Abraham, D.M., Hayal, T. B., Essawi, K., Nguyen, M.A., Stasula, U., Chu, R., et al. (2025). BCL11A +58/+55 enhancer-editing facilitates HSPC engraftment and HbF induction in rhesus macaques conditioned with a CD45 antibody-drug conjugate. Cell Stem Cell *32*, 209–226. https://doi.org/10.1016/j.stem.2024.10.014.

30. Zeng, J., Nguyen, M.A., Liu, P., Da Silva, L.F., Levesque, S., Lin, L.Y., Justus, D.G., Petri, K., Clement, K., Porter, S.N., et al. (2025). Gene editing without ex vivo culture evades genotoxicity in human hematopoietic stem cells. Cell Stem Cell *32*, 191–208. https://doi.org/10.1016/j.stem.2024.11.001.

31. Magnani, A., Pondarré, C., Bouazza, N., Magalon, J., Miccio, A., Six, E., Roudaut, C., Arnaud, C., Kamdem, A., Touzot, F., et al. (2020). Extensive multilineage analysis in patients with mixed chimerism after allogeneic transplantation for sickle cell disease: insight into hematopoiesis and engraftment thresholds for gene therapy. Haematologica *105*, 1240–1247. https://doi.org/10.3324/haematol.2019.227561.

32. Ravi, N.S., Wienert, B., Wyman, S.K., Bell, H.W., George, A., Mahalingam, G., Vu, J.T., Prasad, K., Bandlamudi, B.P., Devaraju, N., et al. (2022). Identification of novel HPFH-like mutations by CRISPR base editing that elevate the expression of fetal hemoglobin. eLife *11*, e65421. https://doi.org/10.7554/eLife.65421.

33. Steinberg, M.H. (2022). Fetal hemoglobin in β hemoglobinopathies: Is enough too much? Am. J. Hematol. *97*, 676–678. https://doi.org/10.1002/ajh.26518.

34. Huang, M.E., Qin, Y., Shang, Y., Hao, Q., Zhan, C., Lian, C., Luo, S., Liu, L. D., Zhang, S., Zhang, Y., et al. (2024). C-to-G editing generates double-strand breaks causing deletion, transversion and translocation. Nat. Cell Biol. *26*, 294–304. https://doi.org/10.1038/s41556-023-01342-2.

35. Liu, P., Keller, J.R., Ortiz, M., Tessarollo, L., Rachel, R.A., Nakamura, T., Jenkins, N.A., and Copeland, N.G. (2003). Bcl11a is essential for normal lymphoid development. Nat. Immunol. *4*, 525–532. https://doi.org/10.1038/ni925.

36. Yu, Y., Wang, J., Khaled, W., Burke, S., Li, P., Chen, X., Yang, W., Jenkins, N.A., Copeland, N.G., Zhang, S., and Liu, P. (2012). Bcl11a is essential for lymphoid development and negatively regulates p53. J. Exp. Med. *209*, 2467–2483. https://doi.org/10.1084/jem.20121846.

37. Guda, S., Brendel, C., Renella, R., Du, P., Bauer, D.E., Canver, M.C., Grenier, J.K., Grimson, A.W., Kamran, S.C., Thornton, J., et al. (2015). miRNA-embedded shRNAs for Lineage-specific BCL11A Knockdown and Hemoglobin F Induction. Mol. Ther. *23*, 1465–1474. https://doi.org/10.1038/mt.2015.113.

38. Bauer, D.E., Kamran, S.C., Lessard, S., Xu, J., Fujiwara, Y., Lin, C., Shao, Z., Canver, M.C., Smith, E.C., Pinello, L., et al. (2013). An Erythroid Enhancer of *BCL11A* Subject to Genetic Variation Determines Fetal Hemoglobin Level. Science *342*, 253–257. https://doi.org/10.1126/science.1242088.

39. Chang, K.-H., Smith, S.E., Sullivan, T., Chen, K., Zhou, Q., West, J.A., Liu, M., Liu, Y., Vieira, B.F., Sun, C., et al. (2017). Long-Term Engraftment and Fetal Globin Induction upon BCL11A Gene Editing in Bone-Marrow-Derived CD34 + Hematopoietic Stem and Progenitor Cells. Mol. Ther. Methods Clin. Dev. *4*, 137–148. https://doi.org/10.1016/j.omtm.2016.12.009.

40. Janoudi, T., Jagdale, M., Wu, M., Gorlla, S., Zhang, P., Shao, Y., Li, L., Bowley, S.R., Marco, E., and Chang, K.-H. (2025). Nonclinical evaluation of *HBG1/2* and *BCL11A* as genome-editing targets for the treatment of β-hemoglobinopathies. Blood Adv. *9*, 808–813. https://doi.org/10.1182/bloodadvances.2024014040.

41. Jang, Y., Feng, R., Palmer, L.E., Mayuranathan, T., Yao, Y., Mayberry, K., Zhou, S., Xu, J., Gossett, J.M., Kang, G., et al. (2025). BCL11A-deficient human erythropoiesis is impaired in vitro and after xenotransplantation into mice. Blood Adv. *9*, 2722–2732. https://doi.org/10.1182/bloodadvances.2024015574.

42. Basak, A., Hancarova, M., Ulirsch, J.C., Balci, T.B., Trkova, M., Pelisek, M., Vlckova, M., Muzikova, K., Cermak, J., Trka, J., et al. (2015). BCL11A deletions result in fetal hemoglobin persistence and neurodevelopmental alterations. J. Clin. Investig. *125*, 2363–2368. https://doi.org/10.1172/JCI81163.

43. Wessels, M.W., Cnossen, M.H., Van Dijk, T.B., Gillemans, N., Schmidt, K. L.J., Van Lom, K., Vinjamur, D.S., Coyne, S., Kurita, R., Nakamura, Y., et al. (2021). Molecular analysis of the erythroid phenotype of a patient with *BCL11A* haploinsufficiency. Blood Adv. *5*, 2339–2349. https://doi.org/10.1182/bloodadvances.2020003753.

44. Newby, G.A., Yen, J.S., Woodard, K.J., Mayuranathan, T., Lazzarotto, C. R., Li, Y., Sheppard-Tillman, H., Porter, S.N., Yao, Y., Mayberry, K., et al. (2021). Base editing of haematopoietic stem cells rescues sickle cell disease in mice. Nature *595*, 295–302. https://doi.org/10.1038/s41586-021-03609-w.

45. Hardouin, G., Antoniou, P., Martinucci, P., Felix, T., Manceau, S., Joseph, L., Masson, C., Scaramuzza, S., Ferrari, G., Cavazzana, M., and Miccio, A. (2023). Adenine base editor–mediated correction of the common and severe IVS1-110 (G>A) β-thalassemia mutation. Blood *141*, 1169–1179. https://doi.org/10.1182/blood.2022016629.

46. Yen, A., Zappala, Z., Fine, R.S., Majarian, T.D., Sripakdeevong, P., and Altshuler, D. (2024). Specificity of CRISPR-Cas9 Editing in Exagamglogene Autotemcel. N. Engl. J. Med. *390*, 1723–1725. https://doi.org/10.1056/NEJMc2313119.

47. Huang, T.P., Zhao, K.T., Miller, S.M., Gaudelli, N.M., Oakes, B.L., Fellmann, C., Savage, D.F., and Liu, D.R. (2019). Circularly permuted and PAM-modified Cas9 variants broaden the targeting scope of base editors. Nat. Biotechnol. *37*, 626–631. https://doi.org/10.1038/s41587-019-0134-y.

48. Walton, R.T., Christie, K.A., Whittaker, M.N., and Kleinstiver, B.P. (2020). Unconstrained genome targeting with near-PAMless engineered CRISPR-Cas9 variants. Science *368*, 290–296. https://doi.org/10.1126/science.aba8853.
49. Kluesner, M.G., Nedveck, D.A., Lahr, W.S., Garbe, J.R., Abrahante, J.E., Webber, B.R., and Moriarity, B.S. (2018). EditR: A Method to Quantify Base Editing from Sanger Sequencing. CRISPR J. *1*, 239–250. https://doi.org/10.1089/crispr.2018.0014.
50. Brinkman, E.K., Chen, T., Amendola, M., and van Steensel, B. (2014). Easy quantitative assessment of genome editing by sequence trace decomposition. Nucleic Acids Res. *42*, e168. https://doi.org/10.1093/nar/gku936.
51. Cradick, T.J., Qiu, P., Lee, C.M., Fine, E.J., and Bao, G. (2014). COSMID: A Web-based Tool for Identifying and Validating CRISPR/Cas Off-target Sites. Mol. Ther. Nucleic Acids *3*, e214. https://doi.org/10.1038/mtna.2014.64.
52. Di Mauro, M., El Hoss, S., Nardo-Marino, A., Stuart-Smith, S., Strouboulis, J., Gibson, J.S., Rees, D.C., and Brewin, J.N. (2023). Males with sickle cell disease have higher risks of cerebrovascular disease, increased inflammation, and a reduced response to hydroxyurea. American J Hematol *98*, E341–E344. https://doi.org/10.1002/ajh.27074.
53. Giarratana, M.-C., Kobari, L., Lapillonne, H., Chalmers, D., Kiger, L., Cynober, T., Marden, M.C., Wajcman, H., and Douay, L. (2005). Ex vivo generation of fully mature human red blood cells from hematopoietic stem cells. Nat. Biotechnol. *23*, 69–74. https://doi.org/10.1038/nbt1047.
54. Tsai, S.Q., Topkar, V.V., Joung, J.K., and Aryee, M.J. (2016). Open-source guideseq software for analysis of GUIDE-seq data. Nat. Biotechnol. *34*, 483. https://doi.org/10.1038/nbt.3534.
55. Tsai, S.Q., Zheng, Z., Nguyen, N.T., Liebers, M., Topkar, V.V., Thapar, V., Wyvekens, N., Khayter, C., Iafrate, A.J., Le, L.P., et al. (2015). GUIDE-seq enables genome-wide profiling of off-target cleavage by CRISPR-Cas nucleases. Nat. Biotechnol. *33*, 187–197. https://doi.org/10.1038/nbt.3117.
56. Clement, K., Rees, H., Canver, M.C., Gehrke, J.M., Farouni, R., Hsu, J.Y., Cole, M.A., Liu, D.R., Joung, J.K., Bauer, D.E., and Pinello, L. (2019). CRISPResso2 provides accurate and rapid genome editing sequence analysis. Nat. Biotechnol. *37*, 224–226. https://doi.org/10.1038/s41587-019-0032-3.
57. Liao, Y., Smyth, G.K., and Shi, W. (2014). featureCounts: an efficient general purpose program for assigning sequence reads to genomic features. Bioinformatics *30*, 923–930. https://doi.org/10.1093/bioinformatics/btt656.
58. Dobin, A., Davis, C.A., Schlesinger, F., Drenkow, J., Zaleski, C., Jha, S., Batut, P., Chaisson, M., and Gingeras, T.R. (2013). STAR: ultrafast universal RNA-seq aligner. Bioinformatics *29*, 15–21. https://doi.org/10.1093/bioinformatics/bts635.
59. Robinson, M.D., McCarthy, D.J., and Smyth, G.K. (2010). edgeR : a Bioconductor package for differential expression analysis of digital gene expression data. Bioinformatics *26*, 139–140. https://doi.org/10.1093/bioinformatics/btp616.
60. Wu, T., Hu, E., Xu, S., Chen, M., Guo, P., Dai, Z., Feng, T., Zhou, L., Tang, W., Zhan, L., et al. (2021). clusterProfiler 4.0: A universal enrichment tool for interpreting omics data. Innovation *2*, 100141. https://doi.org/10.1016/j.xinn.2021.100141.
61. McLaren, W., Gil, L., Hunt, S.E., Riat, H.S., Ritchie, G.R.S., Thormann, A., Flicek, P., and Cunningham, F. (2016). The Ensembl Variant Effect Predictor. Genome Biol. *17*, 122. https://doi.org/10.1186/s13059-016-0974-4.
62. Li, H., and Durbin, R. (2009). Fast and accurate short read alignment with Burrows–Wheeler transform. Bioinformatics *25*, 1754–1760. https://doi.org/10.1093/bioinformatics/btp324.

STAR★METHODS

KEY RESOURCES TABLE

REAGENT or RESOURCE	SOURCE	IDENTIFIER
Antibodies		
CD235a-PE-Cy7	BD Biosciences	Cat #563666;RRID: AB_2738361
HbF-FITC	BD Biosciences	Cat #552829; RRID: AB_394480
Anti-HbS	BioMedomics	Cat #H04181601
anti-rabbit IgG-BV421	BD Biosciences	Cat #565014; RRID: AB_2716308
CD36-V450	BD Horizon	Cat #561535; RRID: AB_10893025
CD71-FITC	BD Biosciences	Cat #555536; RRID: AB_395920
BAND3-PE	IBGRL	Cat #9439
CD49d-APC	BD Biosciences	Cat #559881; RRID: AB_398681
mCD45-VioBlue	Miltenyi Biotec	Cat #130-110-664; RRID: AB_2658223
hCD45-APCvio770	Miltenyi Biotec	Cat #130-110-635; RRID: AB_2658251
CD3-APC	Miltenyi Biotec	Cat #130-113-135; RRID: AB_2725963
CD14-PECy7	BD Biosciences	Cat #562698; RRID: AB_2737729
CD15-PE	Miltenyi Biotec	Cat #130-113-485; RRID: AB_2733765
CD11b-APC	Miltenyi Biotec	Cat #130-110-554; RRID: AB_2654667
CD19-BV510	BD Biosciences	Cat #562947; RRID: AB_2737912
CD235a-PE	BD Biosciences	Cat #555570; RRID: AB_395949
CD71-APC	BD Biosciences	Cat #551374; RRID: AB_398500
CD36-FITC	BD Biosciences	Cat #555454; RRID: AB_2291112
CD34-PE-Vio770	Miltenyi Biotec	Cat #130-124-456; RRID: AB_2811668
CD3 (clone HIT3a, biotinylated)	BD Biosciences	Cat #5555338; RRID: AB_395744
CD19 (clone HIB19, biotinylated)	BD Biosciences	Cat #555411; RRID: AB_395811
B220 (clone RA3-6B2, biotinylated)	BD Biosciences	Cat #553086; RRID: AB_394616
Ter119 (clone TER-119, biotinylated)	BD Biosciences	Cat #553672; RRID: AB_394985
mCD117 (clone 2B8)	BD Biosciences	Cat #553353; RRID: AB_394804
CD3-FITC	BD Biosciences	Cat #561807; RRID: AB_11154575
CD19-PE	BD Biosciences	Cat #345789; RRID: AB_2868815
SA-APC	BD Biosciences	Cat #554067; RRID: AB_10050396
hCD45-BV510	BD Biosciences	Cat #563204; RRID: AB_2738067
CD34-FITC	BD Biosciences	Cat #555821; RRID: AB_396150
CD235a-PE	BD Biosciences	Cat #555570; RRID: AB_395949
CD33-PE-CF594	BD Biosciences	Cat #562492; RRID: AB_2713912
CD38-PE-Cy7	Biolegend	Cat #303516; RRID: AB_2072782
Lin-APC	Biolegend	Cat #348803; RRID: AB_3097661
Anti-Biotin beads	Miltenyi Biotec	Cat #130-090-485
ID-CellStab	Biorad	Cat #005650
Busulfan	Sigma-Aldrich	Cat #55-98-1
Bacterial and virus strains		
pCMV_ABEmax_P2A_GFP	Koblan et al.[22]	Addgene #112101
pCMV_AncBE4max_P2A_GFP	Koblan et al.[22]	Addgene #112100
NG-ABEmax	Huang et al.[47]	Addgene #124163
ABE8e	Richter et al.[14]	Addgene #138489
SpCas9 TadDE	Neugebauer et al.[17]	Addgene #193837

(Continued on next page)

Continued		
REAGENT or RESOURCE	SOURCE	IDENTIFIER
CBE-SpRY-OPT1	Antoniou et al.[19]	N/A
ABE-SpRY-OPT	This paper	N/A
SpRY-ABE8e	This paper	N/A
pCMV-T7-SpRY-P2A-EGFP (RTW4830)	Walton et al.[48]	Addgene #139989
pCMV-T7-ABEmax(7.10)-SpRY-P2A-EGFP (RTW5025)	Walton et al.[48]	Addgene #140003
Biological samples		
Non-mobilized peripheral blood from sickle cell disease patients	Hôpital Necker-Enfants malades	N/A
Mobilized peripheral blood from healthy donors	Hôpital Necker-Enfants malades	N/A
Chemicals, peptides, and recombinant proteins		
StemSpan	STEMCELL Technologies	Cat #9650
Methocult H4435 Enriched	STEMCELL Technologies	Cat #4435
StemRegenin1	STEMCELL Technologies	Cat #72344
Human stem cell factor (SCF)	PeproTech	Cat # 300-07-1MG
FMS-like tyrosine kinase 3 ligand (FLT3)	PeproTech	Cat #300-19-1MG
Thrombopoietin (TPO)	PeproTech	Cat # 300-18-500UG
Interleukin-3 (IL-3)	PeproTech	Cat 200-03-10UG
EPO Eprex	Janssen-Cilag	N/A
Hydrocortisone	Sigma	Cat #H0888
Cas9-GFP protein	Laboratory of Carine Giovannangeli	N/A
DRAQ5	Invitrogen	Cat #65-0880-96
7-AAD	BD Biosciences	Cat #559925
Critical commercial assays		
CD34 MicroBead kit	Miltenyi	Cat #130-046-702
T7 Transcription Kit	MEGAscript	Cat #AM1334
P3 Primary Cell 4D-Nucleofector X Kit S	LONZA	Cat #V4XP-3032
PURE LINK Genomic DNA Mini kit	Life Technologies	Cat #K182002
RNeasy micro kit	QIAGEN	CAT #74106
Quick-DNA/RNA Miniprep	Zymo Research	Cat #D7001
SuperScript First-Strand Synthesis System for RT-qPCR	Invitrogen	Cat # 11904018
iTaq universal SYBR Green master mix	Biorad	Cat #1725120
Deposited data		
Raw nanopore sequencing data	This paper	SRA: PRJNA1192026
RNA-seq data	This paper	GEO: GSE291384
WES-data	This paper	SRA: PRJNA1234896
GUIDE-seq data	This paper	SRA: PRJNA1192026
Scripts used to analyze SRA dataset PRJNA1192026	This paper	https://zenodo.org/records/16736759
Experimental models: Cell lines		
K562 human erythroleukemia cell line	ATCC	Cat #CCL-243
Experimental models: Organisms/strains		
NOD.Cg-KitW-41JTyr+PrkdcscidIl2rgtm1Wjl/ThomJ (NBSGW) mice	Jackson Laboratory	Cat #026622
Oligonucleotides		
sgRNA	N\A	Table S3
Primers to detect on- and off- target BE events and InDels	This paper	Table S3
Primers RT-qPCR	This paper	Table S3
ddPCR primers	This paper	Table S3
sgRNA for Cas9-enrichment library	This paper	Table S3

(*Continued on next page*)

CellPress
OPEN ACCESS

Continued

REAGENT or RESOURCE	SOURCE	IDENTIFIER
Software and algorithms		
EditR	Kluesner et al.[49]	N/A
TIDE	Brinkman et al.[50]	N/A
COSMID	Cradick et al.[51]	N/A
Flow Jo	BD Bioscience	N/A
NexeraX2 SIL-30AC	Shimadzu	N/A
LC Solution software	Shimadzu	N/A
ImageJ Software	Open access	N/A
GraphPad Prism software	GraphPad Software, Inc.	N/A
MinKNOW software	Oxford Nanopore	N/A
Biorender	Biorender.com	N/A
QuantaSoftTM Analysis Pro	BioRad	N/A
CHOPCHOP online design tool	Labun et al.	N/A
Other		
4D-Nucleofector X Unit	Lonza	Cat: AAF-1003X
Gallios Flow cytomer	Beckman Coulter	N/A
NexeraX2 SIL-30AC chromatograph	Shimadzu	N/A
250 × 4.6 mm, 3.6 μm Aeris Widepore column	Phenomenex	N/A
PolyCAT A, PolyLC,	Columbia, MD	N/A
QX200 analyzer	BioRad	N/A
Spinning Disk microscope	Zeiss	N/A
Novocyte Flow cyotmeter	Agilent	N/A

EXPERIMENTAL MODEL AND STUDY PARTICIPANT DETAILS

We obtained adult human non-mobilized $CD34^+$ HSPCs from male and female HD and SCD patients (aged between 20 and 50 years) harboring homozygous SCD mutations. SCD is an autosomal recessive genetic disorder that affects males and females in a similar manner, both in terms of incidence and clinical severity. There is however some evidence to suggest that males experience certain complications more frequently, including stroke and cerebrovascular events, hepatobiliary complications, and hemolysis.[52] To guarantee that during our investigation and development of treatments there was no sex-related bias, we will use primary cells obtained from both male and female patients. Sample size is indicated in the figure legends. HSPCs from each donor were treated with editing reagents targeting the BCL11A enhancers. As controls, HSPCs from each donor were treated either with editing reagents targeting an unrelated locus or only with TE buffer or with the BE mRNA alone. Adult HD and SCD samples eligible for research purposes were obtained from the ‘‘Hôpital Necker-Enfants malades’’ Hospital (Paris, France). Written informed consent was obtained from all adult subjects. All experiments were performed in accordance with the Declaration of Helsinki. The study was approved by the regional investigational review board (reference: DC 2022–5364, CPP Ile-de-France II ‘‘Hôpital Necker-Enfants malades’’). HSPCs were purified by immunomagnetic selection with a manual magnetic cell separator (Miltenyi Biotec) after immunostaining with the CD34 MicroBead Kit (Miltenyi Biotec). Forty-eight hours before transfection, $CD34^+$ cells were thawed and cultured at a concentration of $5x10^5$ cells/ml in the ‘‘HSPC medium’’ containing StemSpan (STEMCELL Technologies) supplemented with penicillin/streptomycin (Gibco), 250 nM StemRegenin1 (STEMCELL Technologies), and the following recombinant human cytokines (PeproTech): human stem cell factor (SCF) (300 ng/mL), FMS-like tyrosine kinase 3 ligand (Flt-3L) (300 ng/mL), thrombopoietin (TPO) (100 ng/mL), and interleukin-3 (IL-3) (60 ng/mL). All HSPC sources were cultured in a 20% O2 and 5% CO2 humidified atmosphere at 37°C.

Transfected $CD34^+$ HSPCs were differentiated into mature RBCs using a three-phase erythroid differentiation protocol, as previously described.[7,53] During the first phase (day 0 to day 6), cells were cultured in a basal erythroid medium supplemented with 100 ng/mL recombinant human SCF (PeproTech), 5 ng/mL recombinant human IL-3 (PeproTech), 3 IU/mL EPO Eprex (Janssen-Cilag) and 10^{-6} M hydrocortisone (Sigma). During the second phase (day 6 to day 9), cells were co-cultured with MS-5 stromal cells in the basal erythroid medium supplemented with 3 IU/mL EPO Eprex (Janssen-Cilag). During the third phase (day 9 to day 20), cells were co-cultured with stromal MS-5 cells in a basal erythroid medium without cytokines. Erythroid differentiation was monitored by flow cytometry analysis of CD36, CD71, CD235a, BAND3, and CD49d erythroid surface markers and of enucleated cells using the DRAQ5 double-stranded DNA dye. 7AAD was used to identify live cells.

NOD.Cg-Kit^{W-41J}Tyr^{+}Prkdc^{scid}Il2rg^{tm1Wjl}/ThomJ (NBSGW) mice were housed in a pathogen-free facility. All experiments were conducted in female mice of 6–9 weeks of age. We used females as they generally support better engraftment of human cells compared to male mice. All experiments and procedures were performed in compliance with the French Ministry of Agriculture's regulations on animal experiments and were approved by the regional Animal Care and Use Committee (APAFIS#2019061312202425_v4). Mice were housed in a temperature- (20°C-22°C) and humidity (40%–50%)-controlled environment with a 12:12 h light-dark cycle and fed *ad libitum* a standard diet.

Human erythroleukemia K562 cells were obtained from ATCC (CCL-243), authenticated by the vendor, and tested for mycoplasma contamination. K562 cells were maintained in RPMI 1640 medium (Gibco –Thermo Fisher Scientific, Waltham, MA, US) containing 2mM glutamine (Sigma, Saint Luis, MO, US) and supplemented with 10% fetal bovine serum (Gibco - Thermo Fisher Scientific, Waltham, MA, US), 10mM HEPES (Sigma, Saint Luis, MO, US), 1mM sodium pyruvate (LifeTechnologies, Carlsbad, CA, US) and 100U/ml each of penicillin and streptomycin (LifeTechnologies, Carlsbad, CA, US).

METHOD DETAILS

Base editor-expressing plasmids

Plasmids used in this study include: pCMV_ABEmax_P2A_GFP (Addgene #112101), pCMV_AncBE4max_P2A_GFP (Addgene #112100), NG-ABEmax (Addgene #124163), ABE8e (Addgene #138489), SpCas9 TadDE (Addgene #193837), CBE-SpRY-OPT1,[19] ABE-SpRY-OPT and SpRY-ABE8e.

A DNA fragment (3′UTR + poly-A) containing two copies of the 3′ untranslated region (UTR) of the *HBB* gene and a poly-A sequence of 96 adenines were purchased from Genscript. ABE-SpRY-OPT plasmid was created by inserting the 3′UTR+poly-A fragment in the pCMV-T7-SpRY-P2A-EGFP (RTW4830) (Addgene #139989) plasmid. SpRY-ABE8e plasmid was created by replacing the Cas9 sequence of the ABE8e plasmid with the SpRY-P2A-EGFP from the pCMV-T7-ABEmax(7.10)-SpRY-P2A-EGFP (RTW5025) (Addgene #140003) plasmid. Plasmids are available upon request.

Single guide RNA design and production

We manually designed sgRNAs targeting the +58-kb and +55-kb regions of *BCL11A* (Table S3). We used chemically modified synthetic gRNAs harboring 2′-*O*-methyl analogs and 3′-phosphorothioate non-hydrolyzable linkages at the first three 5′ and 3′ nucleotides (Synthego).

mRNA *in vitro* transcription

10 μg of BE-expressing plasmids were digested overnight with 20 Units of a restriction enzyme that cleaves once after the poly-A tail. The linearized plasmids were purified using a PCR purification kit (QIAGEN) and were eluted in 30 μL of DNase/RNase-free water. 1 μg of linearized plasmid was used as a template for the *in vitro* transcription (IVT) reaction (MEGAscript, Ambion). The IVT protocol was modified as follows. The GTP nucleotide solution was used at a final concentration of 3.0 mM instead of 7.5 mM, and the anti-reverse cap analog N7-Methyl-3′-*O*-Methyl-Guanosine-5′-Triphosphate-5′-Guanosine (ARCA, Trilink) was used at a final concentration of 12.0 mM, resulting in a final ratio of Cap: GTP of 4:1 that allows efficient capping of the mRNA. The incubation time for the IVT reaction was reduced to 30 min mRNA was precipitated using lithium chloride and resuspended in TE buffer in a final volume that allowed to achieve a concentration of >1 μg/μL. The mRNA quality was evaluated using TapeStation 2200 (Agilent).

RNA transfection

$1x10^5$ to $2x10^5$ or $3x10^6$ CD34$^+$ HSPCs per condition were transfected with 3.0 μg or 15.0 μg of the enzyme-encoding mRNA, respectively, and a synthetic sgRNA at a final concentration of 2.3 μM. We used the P3 Primary Cell 4D-Nucleofector X Kit S or L (Lonza) and the CA137 program (Nucleofector 4D). Untransfected cells or cells transfected with TE buffer or with the enzyme-encoding mRNA only, or with the enzyme-encoding mRNA and a sgRNA targeting the *AAVS1* locus, served as negative controls.

Ribonucleoprotein (RNP) transfection

RNP complexes were assembled at room temperature using a 90 μM Cas9-GFP protein and a 180 μM synthetic sgRNA (ratio Cas9: sgRNA of 1:2). CD34$^+$ HSPCs ($2x10^5$ cells/condition) were transfected with RNP complexes using the P3 Primary Cell 4D-Nucleofector X Kit S (Lonza) and the CA137 program (Nucleofector 4D) in the presence of a transfection enhancer (IDT). Untransfected cells or cells transfected with TE buffer served as negative controls.

Colony-forming cell assay

CD34$^+$ HSPCs were plated at $1x10^3$ cells/mL concentration in a methylcellulose-based medium (Stem Cell Technologies) under conditions supporting erythroid and granulo-monocytic differentiation. BFU-E and CFU-GM colonies were counted after 14 days. Colonies were randomly picked and collected as bulk populations (containing at least 25 colonies) to evaluate base editing efficiency, globin expression by RT-qPCR and RP-HPLC, and hemoglobin expression by CE-HPLC. BFU-Es were randomly picked and collected as single colonies (around 30 colonies per sample) to evaluate base editing efficiency and globin expression by RT-qPCR.

Genome-wide, unbiased identification of double-strand breaks enabled by sequencing (GUIDE-seq)

Human erythroleukemia K562 cells (2.5×10^5) were transfected with 500 ng of Cas9-expressing plasmid (Cas9-nuclease corresponding to the Cas9 nickase included in BE (Cas9-NGG)), together with 250 ng of each sgRNA–coding plasmid or an empty pUC19 vector (background control), 10 pmol of the bait double-stranded oligodeoxynucleotide (dsODN) (designed according to the original GUIDE-seq protocol[54]; and 50 ng of a pEGFP-IRES-Puro plasmid, expressing both enhanced GFP (EGFP) and the puromycin resistance genes. One day after transfection, cells were selected with puromycin (1 μg/mL) for 48 h to enrich for transfected cells. Cells were then collected, and genomic DNA was extracted using the DNeasy Blood and Tissue Kit (Qiagen) and sheared using the Covaris S200 sonicator to an average length of 500 bp. Library preparation was performed using the original adapters and primers according to previous work.[55]

End-repair reaction was performed using NEBNext Ultra End Repair/dA Tailing Module and adaptor ligation using NEBNext UltraTM Ligation Module, as previously described. Amplification steps were then performed following the GUIDE-seq protocol previously described.[55]

Libraries were sequenced with a MiSeq sequencing system (Illumina) using the Illumina MiSeq Reagent kit V2-300 cycles (paired-end sequencing; 2×150-bp). Raw sequencing data (FASTQ files) were analyzed using the GUIDE-seq computational pipeline.[54] The cutoff was set to a level of detection of at least three reads. Identified sites were considered *bona fide* off-targets if a maximum of six mismatches against the on-target were present and if they were absent in the background control.

Evaluation of editing efficiency

Base editing efficiency and InDel frequency were evaluated in HSPC-derived erythroid cells at the end of the first phase of differentiation and in BFU-E and CFU-GM 14 days after plating. Genomic DNA was extracted from control and edited cells using the PURE LINK Genomic DNA Mini kit (Life Technologies) or Quick-DNA/RNA Miniprep (ZYMO Research), following manufacturers' instructions. To evaluate base editing efficiency at sgRNA target sites, we performed PCR using primers listed in Table S3, followed by Sanger sequencing and EditR analysis.[49] TIDE analysis (Tracking of InDels by Decomposition) was also performed to evaluate the percentage of InDels in edited samples.[50]

On- and off-target regions were also PCR-amplified and subjected to NGS. We selected the top 10 predicted off-targets (top 5 nominated by GUIDE-seq and top five nominated by COSMID *in silico* analysis[51] using default parameters of mismatch tolerance of $\leq$3 and DNA bulge size of 1, following the COSMID algorithm criteria) and assessed editing at day 6 of culture. On-target and off-target sites were PCR-amplified using the Phusion High-Fidelity polymerase (M0530; NEB, Ipswich, MA) and primers containing specific DNA stretches (MR3 for forward primers and MR4 for reverse primers; Table S3) located 5′ to the sequence recognizing the off-target. Amplicons were purified using Ampure XP beads (A63881; Beckman Coulter, Brea, CA). Illumina-compatible barcoded DNA amplicon libraries were prepared by a second PCR step using the Phusion High-Fidelity polymerase (M0530; NEB) and primers containing Unique Dual Index barcodes and annealing to MR3 and MR4 sequences. Libraries were pooled, purified using the High Pure PCR product purification kit (11732676001; Sigma-Aldrich, Saint Louis, MO), and sequenced using the Illumina NovaSeq 6000 system (paired-end sequencing; 2×100-bp) to obtain a minimum of 100,000 reads per amplicon. Targeted NGS data were analyzed using CRISPResso2.[56]

RT-qPCR

Total RNA was extracted from SCD HSPCs differentiated toward the erythroid lineage (day 13) using the RNeasy micro kit (QIAGEN) and from pools of BFU-E and single colonies using the Quick-DNA/RNA Miniprep kit (ZYMO Research). RNA was treated with DNase using the DNase I kit (Invitrogen), following the manufacturer's instructions. Mature transcripts were reverse-transcribed using SuperScript First-Strand Synthesis System for RT-qPCR (Invitrogen) with oligo (dT) primers. RT-qPCR was performed using primers listed in Table S3, the iTaq universal SYBR Green master mix (Biorad), and the CFX384 Touch Real-Time PCR Detection System (Biorad). γ- and β^S-globin mRNA expression was normalized to α-globin mRNA. *BCL11A* mRNA expression was normalized to *GAPDH* mRNA.

Flow cytometry analysis

HSPC-derived erythroid cells were fixed with 0.05% cold glutaraldehyde and permeabilized with 0.1% Triton X-100. After fixation and permeabilization, cells were stained with an antibody recognizing CD235a erythroid surface marker (PE-Cy7-conjugated anti-CD235a antibody, 563666, BD Bioscience) and either an antibody recognizing HbF (FITC-conjugated anti-HbF antibody, clone 2D12, 552829 BD) or an antibody recognizing HbS (anti-HbS antibody, H04181601, BioMedomics), followed by the staining with a secondary antibody recognizing rabbit IgG (BV421-conjugated anti-rabbit IgG, 565014, BD). Flow cytometry analysis of CD36, CD71, CD235a, BAND3, and CD49d erythroid surface markers was performed using a V450-conjugated anti-CD36 antibody (561535, BD Horizon), an FITC-conjugated anti-CD71 antibody (555536, BD Bioscience), a PE-Cy7-conjugated anti-CD235a antibody (563666, BD Bioscience), a PE-conjugated anti-BAND3 antibody (9439, IBGRL), and an APC-conjugated anti-CD49d antibody (559881, BD). Flow cytometry analysis of enucleated or viable cells was performed using double-stranded DNA dyes (DRAQ5, 65-0880- 96, Invitrogen, and 7AAD, 559925, BD, respectively). Flow cytometry analyses were performed using Gallios (Beckman Coulter) flow cytometers. Data were analyzed using the FlowJo (BD Biosciences) software.

Reverse phase high-performance liquid chromatography analysis of globin chains

RP-HPLC analysis was performed using a NexeraX2 SIL-30AC chromatograph and the LC Solution software (Shimadzu). A 250 × 4.6 mm, 3.6 μm Aeris Widepore column (Phenomenex) was used to separate globin chains by HPLC. Samples were eluted with a gradient mixture of solution A (water/acetonitrile/trifluoroacetic acid, 95:5:0.1) and solution B (water/acetonitrile/trifluoroacetic acid, 5:95:0.1). The absorbance was measured at 220 nm. γ-globin expression was normalized to α-globin.

CE-HPLC analysis of hemoglobin tetramers

Cation-exchange HPLC analysis was performed using a NexeraX2 SIL-30AC chromatograph and the LC Solution software (Shimadzu). A 2-cation-exchange column (PolyCAT A, PolyLC, Columbia, MD) was used to separate hemoglobin tetramers by HPLC. Samples were eluted with a gradient mixture of solution A (20mM bis Tris, 2mM KCN, pH = 6.5) and solution B (20mM bis Tris, 2mM KCN, 250mM NaCl, pH = 6.8). The absorbance was measured at 415 nm. The percentage of each Hb type was calculated over the total Hb tetramer.

Digital droplet PCR

The 3.2-kb deletion and inversion frequencies were measured by Digital Droplet PCR (ddPCR) using a primer/probe mix (Bio-Rad) containing the primers and probes listed in Table S3. Control primers annealing to hALB (located on chr 4) were used as DNA loading control (Table S3). Synthetic double-stranded DNA fragments recapitulating the sequence generated after the occurrence of the 3.2-kb deletion or inversion served as positive controls.

The abundance of BCL11A-XL mRNA in control and edited samples was evaluated by ddPCR. cDNA was prepared as previously described. A primer/probe mix (Bio-Rad) containing the primers and probes listed in Table S3 was used to detect BCL11A-XL. Synthetic double-stranded fragments containing BCL11A-XL sequences were used as positive controls. To detect GAPDH, we used the TaqMan Gene Expression assays, VIC (Thermo). *BCL11A* mRNA expression was normalized to *GAPDH* mRNA.

Data were acquired through a QX200 analyzer (Bio-Rad), and results were analyzed with QuantaSoftTM Analysis Pro (Bio-Rad). A positive droplet count threshold was set at 30 to allow proper calculation of copy/μL concentration through the application of the Poisson distribution.

Sickling assay

180,000 *in vitro* differentiated RBCs (at day 19 or 20) were resuspended in CellStab (BioRad), placed in an 8-well m-Slide (Ibidi), and incubated under gradual hypoxic conditions (20% O_2 for 20 min; 10% O_2 for 20 min; 5% O_2 for 20 min; 0% O_2 for 60-180min). A time course analysis of sickling was performed in real time by video microscopy. Images were captured every 20 min using a Zeiss Spinning Disk microscope and a 40x objective. Throughout the time course, images were captured and then processed with ImageJ to determine the percentage of non-sickle RBCs per field of acquisition in the total RBC population. More than 400 cells were counted per condition. Counting was normalized to the mock.

Single guide RNA design for Cas9-enrichment library preparation

SgRNAs were designed to introduce cuts on complementary strands flanking the region of interest (ROI), using the CHOPCHOP online design tool (https://chopchop.cbu.uib.no/) and selected for the highest predicted on-target efficiency and minimal off-target activity. The sgRNAs were assembled as a duplex from synthetic CRISPR RNAs (crRNAs) (Integrated DNA Technologies-IDT, custom designed) and *trans*-activating crRNAs (tracrRNAs) (IDT, catalog no. 1072532). SgRNA sequences are provided in Table S3. The ROI was centered on the expected cut sites of the gene editing approaches on the *BCL11A* gene and has a size of 14.3 kb.

Cas9-enrichment library preparation for nanopore sequencing

High molecular weight DNA was extracted from the patient's HSPC with the Nanobind CBB kit (PacBio, catalog no. 102-301-900) according to the manufacturer's instructions. DNA was size selected using Short Read Eliminator XS kit (PacBio, catalog no. 102-208-200) and quantified using the Qubit fluorometer (Thermo Fisher Scientific). 5 μg of DNA was used for the library preparation using the Cas9 Sequencing Kit (Oxford Nanopore Technologies-ONT, SQK-CS9109) and following the Cas9-mediated PCR-free enrichment protocol (version: CAS_9106_v109_revC_16Sep2020) available through ONT. Libraries were loaded onto MinION flow cells with R9.4.1 nanopores (ONT, catalog no. FLO-MIN106D). One flow cell was used per biological condition and run on GridION using MinKNOW software for 72 h.

Bioinformatic analysis of Cas9-enrichment library from nanopore sequencing

Raw reads from Nanopore sequencing were preprocessed using cutadapt (v4.4) to remove low-quality bases (q = 5 threshold at 5′ and 3′ ends) and to select reads longer than 4 kb. Adaptors were then trimmed using porechop (v 0.2.4), and reads were aligned on the human reference genome (GRCh38.p13) using minimap2 (v 2.26-r1175; arguments*: -x map-ont -a -Y – secondary=no*). Reads that align at the enrichment locus (11:5220519-55121185) were re-aligned on this region using minimap2 *(*arguments*: -x map-ont -n 20 -I 1K -r 400,1000 -k 15 -w 15 -a -Y – secondary=no).*

Finally, the depth of coverage at each position was measured using samtools (v1.17), the base composition extracted using pysamstats (v1.1.2), and the presence of INDELS detected using the variant caller sniffles (v 1.0.12; arguments*: -n -1 -r 1000 -s 1 -day 1 -L 4*). Additional processing was performed using R (v4.4.1) to prepare figures and tables. InDels were classified as small, intermediate, and large according to their size ([4-50bp], [51-200bp], >200bp). The co-editing at GATA1 and ATF4 loci was assessed using mpileup output from samtools (1.21) using a 5-bp window around editing positions of the respective sgRNA (GATA_bs_3 and ATF4_bs_2).

Script & pipeline available on demand. Raw data were deposited on SRA under accession number PRJNA1192026.

RNA-seq

Total RNA was isolated from HD HSPCs 48 h after RNA transfection using the RNeasy Kit (QIAGEN), including a DNase treatment step. Libraries were prepared using 30–50 ng of total RNA with a Watchmaker RNA kit, incorporating rRNA/Globin Polaris Depletion, following the manufacturer's recommendations. On average, 235 million paired-end reads were produced per exome library. Read quality was assessed using FastQC [version 0.11.9; https://www.bioinformatics.babraham.ac.uk/projects/fastqc/]. Adapter sequences and low-quality bases (Q < 20) were trimmed from raw reads with BBDuk [version 38.92; https://sourceforge.net/projects/bbmap/]; moreover, the first 10 nucleotides were force-trimmed for low quality. Reads shorter than 35 bp post-trimming were discarded. Trimmed reads were aligned to the human reference genome (hg38) using STAR [version 2.7.9a]. Raw gene counts were generated in R-4.1.1 using the featureCounts function of the Rsubread package [version 2.8.1[57,58]] and the GENCODE 44 basic gene annotation for the hg38 reference genome. Raw gene counts were normalized to counts per million mapped reads (CPMs) and to fragments per kilobase of exon per million mapped reads (FPKMs) using the edgeR R package [version 3.36.0[59]]; only genes with a CPM greater than 1 in at least 3 samples were retained for differential analysis. Differential gene expression analysis was performed using the glmQLFTest function of the edgeR R package, using the donor as a blocking variable. Genes with FDR <0.05 and absolute log2FC ≥1 were defined as differentially expressed. Functional enrichment analysis was performed with the clusterProfiler R package [version 4.12.5[60]]. Fastq files and gene expression matrix from RNA-seq have been deposited in the GEO database (https://www.ncbi.nlm.nih.gov/geo/) under accession code GSE291384.

Detection of RNA editing events by RNA-seq

RNA editing analysis was performed according to GATK Best Practices for RNA-seq variant calling (GATK v4.2.2.0). In brief, trimmed reads were two-pass aligned to the hg38 human reference genome with STAR (v. 2.7.9a)[58] using parameters to specify the ReadGroup and output the aligned BAM file sorted by coordinate; then duplicates were marked using GATK *MarkDuplicates*. After splitting reads containing Ns in their cigar string because they span splicing sites using GATK *SplitNCigarReads*, base quality recalibration was performed using GATK *BaseRecalibrator* and *ApplyBQSR*, and the known variants collected in dbSNP155. RNA base-editing variant calling was performed using GATK *HaplotypeCaller* only on canonical (1–22, X, Y, and M) chromosomes. Single-nucleotide variants (SNVs) were hard-filtered using GATK *VariantFiltration,* applying suggested basic thresholds from GATK Best Practices. SNVs annotation was performed using the Variant Effect Predictor (VEP) tool from Ensembl.[61] Multiallelic variants (mainly involving repetitive sequences) were removed. Only SNVs with coverage ≥30 reads and genotype quality ≥30 were retained in each sample. To define SNVs private to treated samples (CBE, DBE, and Cas9), we required a reference allele frequency ≥0.99 in the untreated sample (mock) at the position of the variant.

Whole-exome sequencing

Genomic DNA was isolated from HD HSPCs 48 h after RNA transfection using the Quik-DNA/RNA Miniprep kit (Zymo), following the manufacturer's instructions. Exome libraries were prepared using 10 ng–50 ng of total DNA using the Twist Human Core Exome (+RefSeq) kit as recommended by the manufacturer. On average, 235 million paired-end reads were produced per exome library. Read quality was evaluated using FastQC (v. 0.11.9). Adapters and low-quality tails (quality < Q20) were trimmed from raw reads with BBDuk (v. 38.92). Reads shorter than 35 bp after trimming were removed.

Variant calling was carried out according to GATK Best Practices for germline short variant discovery (GATK v4.2.2.0). In brief, FASTQ files were mapped on the hg38 human reference genome with BWA (v 0.7.17),[62] specifying the ReadGroup. Duplicates were marked using GATK *MarkDuplicates*. Base quality recalibration was performed using GATK *BaseRecalibrator* and *ApplyBQSR*, specifying the list of target exons with a padding region of 100 bp. Variant calling was performed using GATK *HaplotypeCaller* only on canonical (1–22, X, Y, and M) chromosomes. SNVs and InDels were hard-filtered using GATK *VariantFiltration,* applying suggested basic thresholds from GATK Best Practices. SNVs annotation was performed using the VEP tool from Ensembl.[61] Multiallelic variants (mainly involving repetitive sequences) were removed. Only SNVs with coverage ≥30 reads and genotype quality ≥30 were retained in each sample. To define SNVs private to treated samples (CBE, DBE, and Cas9), we required a reference allele frequency ≥0.99 in the untreated sample (mock) at the position of the variant.

Fastq files generated by WES have been deposited in the SRA database (https://www.ncbi.nlm.nih.gov/sra) under accession code PRJNA1234896.

Hematopoietic stem/progenitor cell xenotransplantation in NOD.Cg-KitW−-41JTyr+PrkdcscidIl2rgtm1Wjl/ThomJ mice

Control or edited mobilized CD34+ cells (3.5×10^5 cells per mouse) were transplanted into non-irradiated mice via retro-orbital sinus injection. NBSGW female mice were conditioned with busulfan (Sigma-Aldrich) injected intraperitoneally (15 mg/kg body weight) 24 h before transplantation. Sixteen weeks after transplantation, NBSGW primary recipients were euthanized. Cells were harvested from BM, thymus, spleen, and blood, and stained with antibodies against the following murine and human surface markers: murine CD45 (1/50 mCD45-VioBlue; Miltenyi Biotec), human CD45 (1/50 hCD45-APCvio770; Miltenyi Biotec). BM cells were also stained with human CD3 (1/50 CD3-APC; Miltenyi Biotec), human CD14 (1/50 CD14-PECy7; BD Biosciences), human CD15 (1/50 CD15-PE; Miltenyi Biotec), human CD11b (1/100 CD11b-APC; Miltenyi Biotec), human CD19 (1/100 CD19-BV510; BD Biosciences), human CD235a (1/50 CD235a-PE; BD Biosciences), human CD71 (1/10 CD71-APC; BD Biosciences), CD36 (1/50 CD36-FITC; BD Bio-sciences), and CD34 (1/100 CD34-PE-Vio770; Miltenyi Biotec). Cells were analyzed by flow cytometry using the Novocyte analyzer (Agilent) and the FlowJo software (BD Biosciences).

Human BM CD45+ cells were sorted by immunomagnetic selection with CD45 MicroBeads (Miltenyi Biotec). Furthermore, BM cells were subjected to immunostaining with biotinylated antibodies that recognized the following surface markers: CD3 (dilution 1/25, clone HIT3a; BD), CD19 (dilution 1/25, clone HIB19; BD), B220 (dilution 1/50, clone RA3-6B2; BD), Ter119 (dilution 1/50, clone TER-119; BD), and mCD117 (clone 2B8; BD). BM cells were washed and incubated with 20 µL of Anti-Biotin beads (Miltenyi Biotec). After washing, the cells were magnetically purified using an LS column (Miltenyi Biotec) according to the manufacturer's instructions. Cells from the positive fraction were immuno-stained with the following antibodies: CD19-PE (dilution 1/20, BD), streptavidin (SA)-APC (dilution 1/20, BD), and hCD45-BV510 (dilution 1/100, BD). The hCD45^{high}/CD19^{high} cells were sorted using the MA900 cell sorter and subjected to RT-qPCR analysis. Cells from the negative fraction were immuno-stained with the following antibodies: CD235a-PE (dilution 1/5000, BD) and hCD45-BV510 (dilution 1/100, BD). The hCD45$^{low/-}$/CD235a^{high} cells were sorted using the MA900 cell sorter (Sony Biotechnology, San Jose, CA) and subjected to flow cytometry, RP-HPLC, and RT-qPCR analysis.

QUANTIFICATION AND STATISTICAL ANALYSIS

The number of biologically independent samples, animals, or experiments (n) is indicated in the figure legends. The number of human donors used per experiment is also specified in the legends. Data are presented as median or mean ± SEM, as appropriate based on data distribution. One-way or two-way ANOVA with correction for multiple comparisons and Multiple t test was used to assess group differences where applicable, as detailed in the figure legends. Statistical analyses were performed using GraphPad Prism (v10.0.2; GraphPad Software) and R (v4.4.1; https://cran.r-project.org). Statistical significance was defined as *$p \leq 0.05$ (*), $p \leq 0.01$ (**), $p \leq 0.001$ (***), $p \leq 0.0001$ (****), and ''ns'' stands for not significant.

Cell Reports Medicine, Volume *6*

Supplemental information

Multiplex base editing of *BCL11A* regulatory elements to treat sickle cell disease

Letizia Fontana, Pierre Martinucci, Simone Amistadi, Tristan Felix, Margaux Mombled, Alexandra Tachtsidi, Guillaume Corre, Anne Chalumeau, Giulia Hardouin, Jeanne Martin, Oriana Romano, Mario Amendola, Panagiotis Antoniou, and Annarita Miccio

SUPPLEMENTARY FIGURE TITLES AND LEGENDS

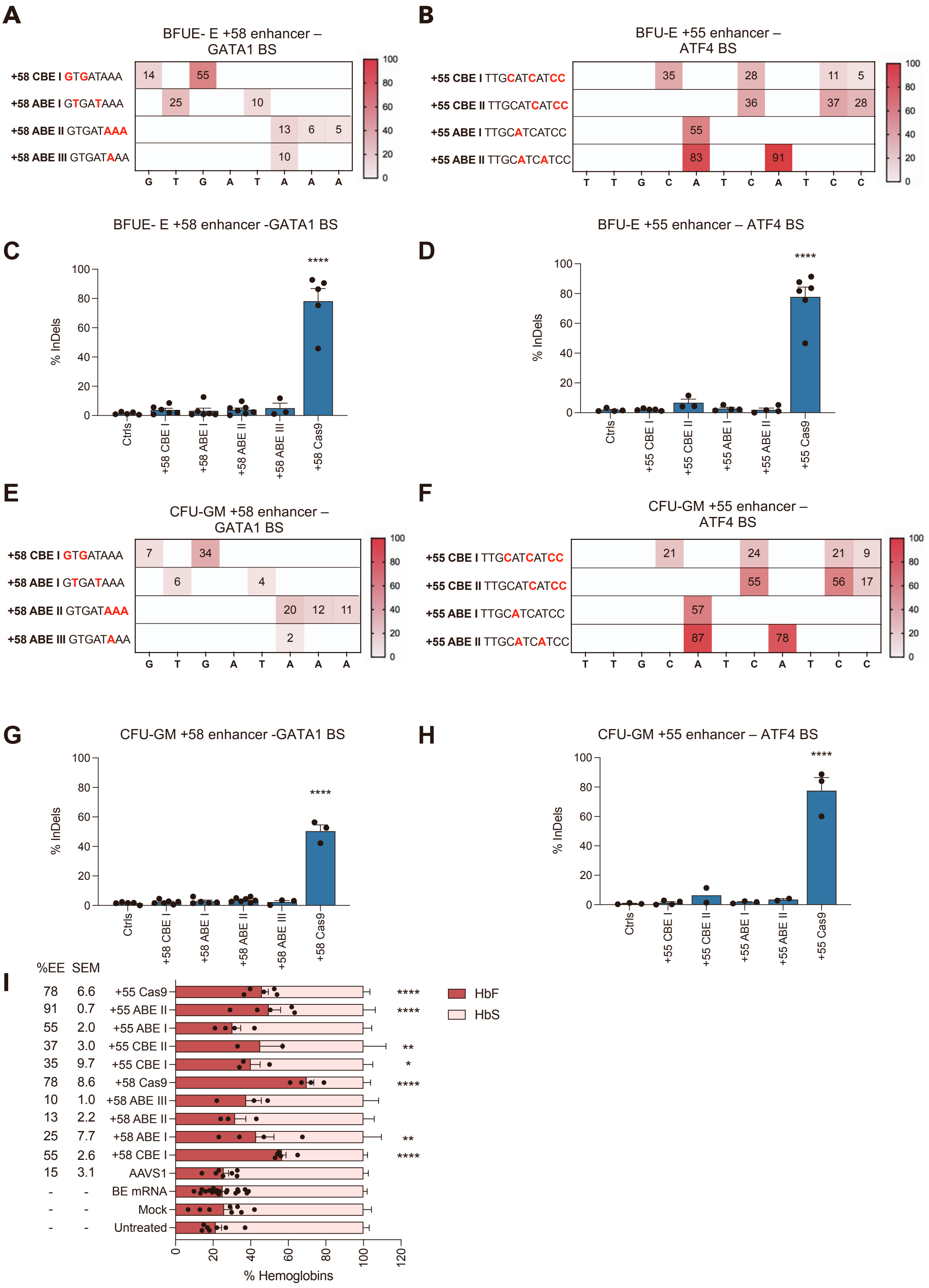

Figure S1. Base editing of the erythroid-specific *BCL11A* enhancers in SCD HPSC-derived BFU-E and CFU-GM colonies.

A-B. C-G to T-A or A-T to G-C base-editing efficiency calculated by the EditR software in samples subjected to Sanger sequencing in pools of BFU-E colonies derived from SCD HSPCs edited in the +58-kb (**A**) or +55-kb (**B**) regions. Data are expressed as mean (n = 2 to 4 biologically independent experiments, 2 to 5 donors).

C-D. Frequency of InDels, measured by TIDE analysis, in samples subjected to Sanger sequencing in pools of BFU-E colonies derived from SCD HSPCs edited in the +58-kb (**C**) or +55-kb (**D**) regions. Data are expressed as mean ± SEM (n = 2 to 4 biologically independent experiments, 2 to 5 donors). ****P ≤ 0.0001 (One-way ANOVA. Comparison of controls vs edited samples)

E-F. C-G to T-A or A-T to G-C base-editing efficiency calculated by the EditR software in samples subjected to Sanger sequencing in pools of CFU-GM colonies derived from SCD HSPCs edited in the +58-kb (**E**) or +55-kb (**F**) regions. Data are expressed as mean (n = 2 to 4 biologically independent experiments, 2 to 5 donors).

G-H. Frequency of InDels, measured by TIDE analysis, in samples subjected to Sanger sequencing in pools of CFU-GM colonies derived from SCD HSPCs edited in the +58-kb (**G**) or +55-kb (**H**) regions. Data are expressed as mean ± SEM (n = 2 to 4 biologically independent experiments, 2 to 5 donors). ****P ≤ 0.0001 (One-way ANOVA. Comparison of controls vs edited samples)

I. Analysis of HbF and HbS by cation-exchange HPLC in pools of BFU-E colonies. We calculated the percentage of each Hb type over the total Hb tetramers. Data are expressed as mean ± SEM (n = 3 to 4 biologically independent experiments, 3 to 4 donors). *P ≤ 0.05; **P ≤ 0.01; ****P ≤ 0.0001 (two-way ANOVA with Dunnett correction for multiple comparisons. Statistical significance between mock and edited samples is depicted in the graph).

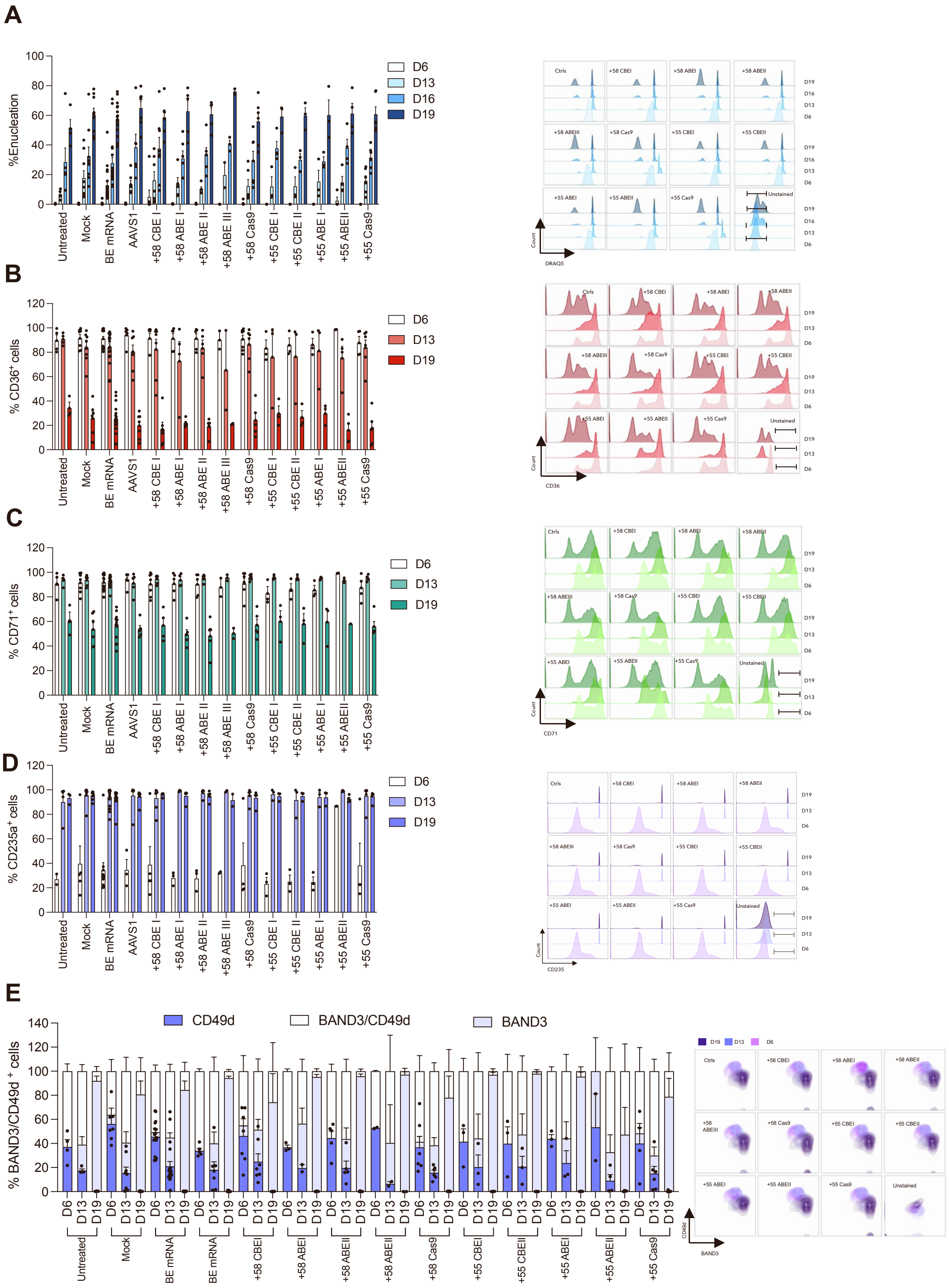
A
%Enucleation
D6
D13
D16
D19
Untreated
Mock
BE mRNA
AAVS1
+58 CBE I
+58 ABE I
+58 ABE II
+58 ABE III
+58 Cas9
+55 CBE I
+55 CBE II
+55 ABE I
+55 ABEII
+55 Cas9
Ctrls
+58 CBEI
+58 ABEI
+58 ABEII
+58 ABEIII
+58 Cas9
+55 CBEI
+55 CBEII
+55 ABEI
+55 ABEII
+55 Cas9
Unstained
Count
DRAQ5
B
% CD36+ cells
D6
D13
D19
CD36
C
% CD71+ cells
CD71
D
% CD235a+ cells
CD235
E
% BAND3/CD49d + cells
CD49d
BAND3/CD49d
BAND3
D6
D13
D19
Untreated
Mock
BE mRNA
+58 CBEI
+58 ABEI
+58 ABEII
+58 ABEII
+58 Cas9
+55 CBEI
+55 CBEII
+55 ABEI
+55 ABEII
+55 Cas9
CD49d
BAND3

Figure S2. Erythroid differentiation of SCD HSPCs upon base editing of the erythroid-specific *BCL11A* enhancers.

A. Frequency of enucleated cells at day 6, 13, 16, and 19 of erythroid differentiation, as measured by flow cytometry analysis of DRAQ5 nuclear staining in control and edited samples. Data are expressed as mean ± SEM (n = 2 to 6 biologically independent experiments, 2 to 5 donors). Representative flow cytometry histograms showing the DRAQ5$^-$ cell population for control and edited samples are reported.

B-D. Frequency of CD36$^+$ (**B**), CD71$^+$ (**C**), and CD235a$^+$ (**D**) cells at day 6, 13, and 19 of erythroid differentiation, as measured by flow cytometry analysis of CD36, CD71, and CD235a erythroid markers. Data are expressed as mean ± SEM (n = 2 to 6 biologically independent experiments, 2 to 5 donors). Representative flow cytometry histograms showing the CD36$^+$ (**B**), CD71$^+$ (**C**), and CD235a$^+$ (**D**) cell population for control and edited samples are reported.

E. Frequency of CD49d$^+$, BAND3$^+$ and CD49d$^+$/BAND3$^+$ in 7AAD$^-$/CD235a$^+$ cells at day 6, 13, and 19 of erythroid differentiation, as measured by flow cytometry analysis of CD49d and BAND3 erythroid markers. Data are expressed as mean ± SEM (n = 2 to 6 biologically independent experiments, 2 to 5 donors). Representative flow cytometry contour plots showing the CD49d$^+$, BAND3$^{+,}$ and CD49d$^+$/BAND3$^+$ cell population for control and edited samples are reported.

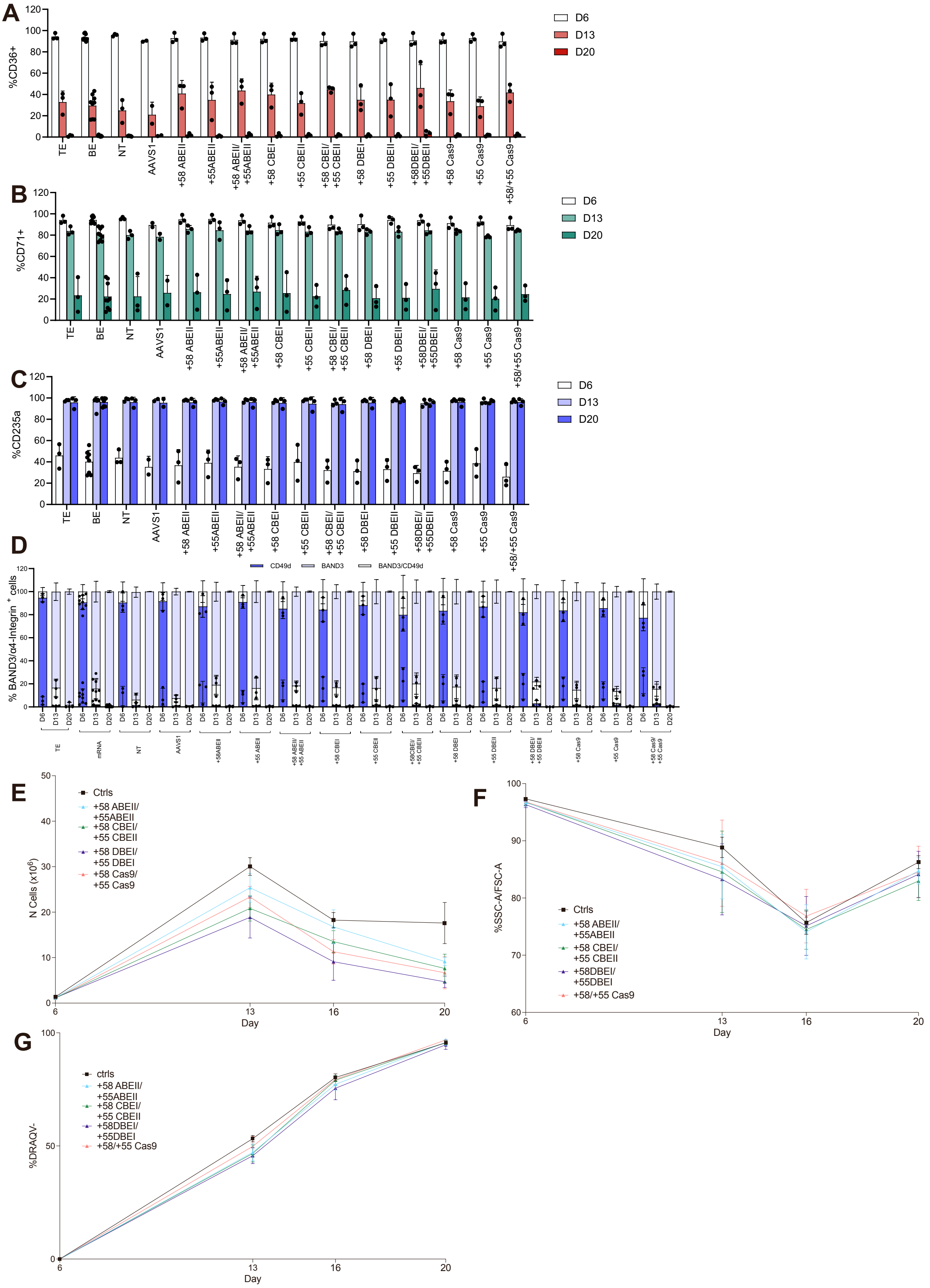
A
%CD36+
D6
D13
D20
TE
BE
NT
AAVS1
+58 ABEII
+55ABEII
+58 ABEII/
+55ABEII
+58 CBEI
+55 CBEII
+58 CBEI/
+55 CBEII
+58 DBEI
+55 DBEII
+58DBEI/
+55DBEII
+58 Cas9
+55 Cas9
+58/+55 Cas9
B
%CD71+
C
%CD235a
D
CD49d
BAND3
BAND3/CD49d
% BAND3/α4-Integrin + cells
D6
D13
D20
mRNA
E
N Cells (x10^6)
Day
Ctrls
+58 ABEII/
+55ABEII
+58 CBEI/
+55 CBEII
+58 DBEI/
+55 DBEI
+58 Cas9/
+55 Cas9
F
%SSC-A/FSC-A
+58DBEI/
+55DBEI
+58/+55 Cas9
G
%DRAQV-
ctrls

Figure S3. Erythroid differentiation and cell growth of SCD HSPCs after single- and multiplex base editing of the erythroid-specific *BCL11A* enhancers.

A-C. Frequency of $CD36^+$ (**A**), $CD71^+$ (**B**), and $CD235a^+$ (**C**) cells at day 6, 13, and 20 of erythroid differentiation, as measured by flow cytometry analysis of CD36, CD71, and CD235a erythroid markers. Data are expressed as mean ± SEM (n = 3 biologically independent experiments, 3 donors).

D. Frequency of $CD49d^+$, $BAND3^+$ and $CD49d^+/BAND3^+$ in $7AAD^-/CD235a^+$ cells at day 6, 13, and 19 of erythroid differentiation, as measured by flow cytometry analysis of CD49d and BAND3 erythroid markers. Data are expressed as mean ± SEM (n = 3 biologically independent experiments, 3 donors).

E. Analysis of the cell growth over time. The number of cells was measured at multiple time points to assess growth dynamics (day 6,13,16, and 20 of erythroid differentiation). Data are expressed as mean ± SEM (n = 3 biologically independent experiments, 3 donors).

F. Analysis of cell viability over time. We reported the percentage of live cells over the total live and dead/apoptotic cells identified based on size and granularity. Viability was assessed at days 6, 13, 16, and 20 of erythroid differentiation. Data are presented as mean ± SEM (n = 3 biologically independent experiments, from 3 donors).

G, Frequency of enucleated cells as measured by flow cytometry analysis of DRAQ5 nuclear staining in control and edited samples was reported at day 6, 13, 16, and 20 of erythroid differentiation. Data are expressed as mean ± SEM (n = 3 biologically independent experiments, 3 donors).

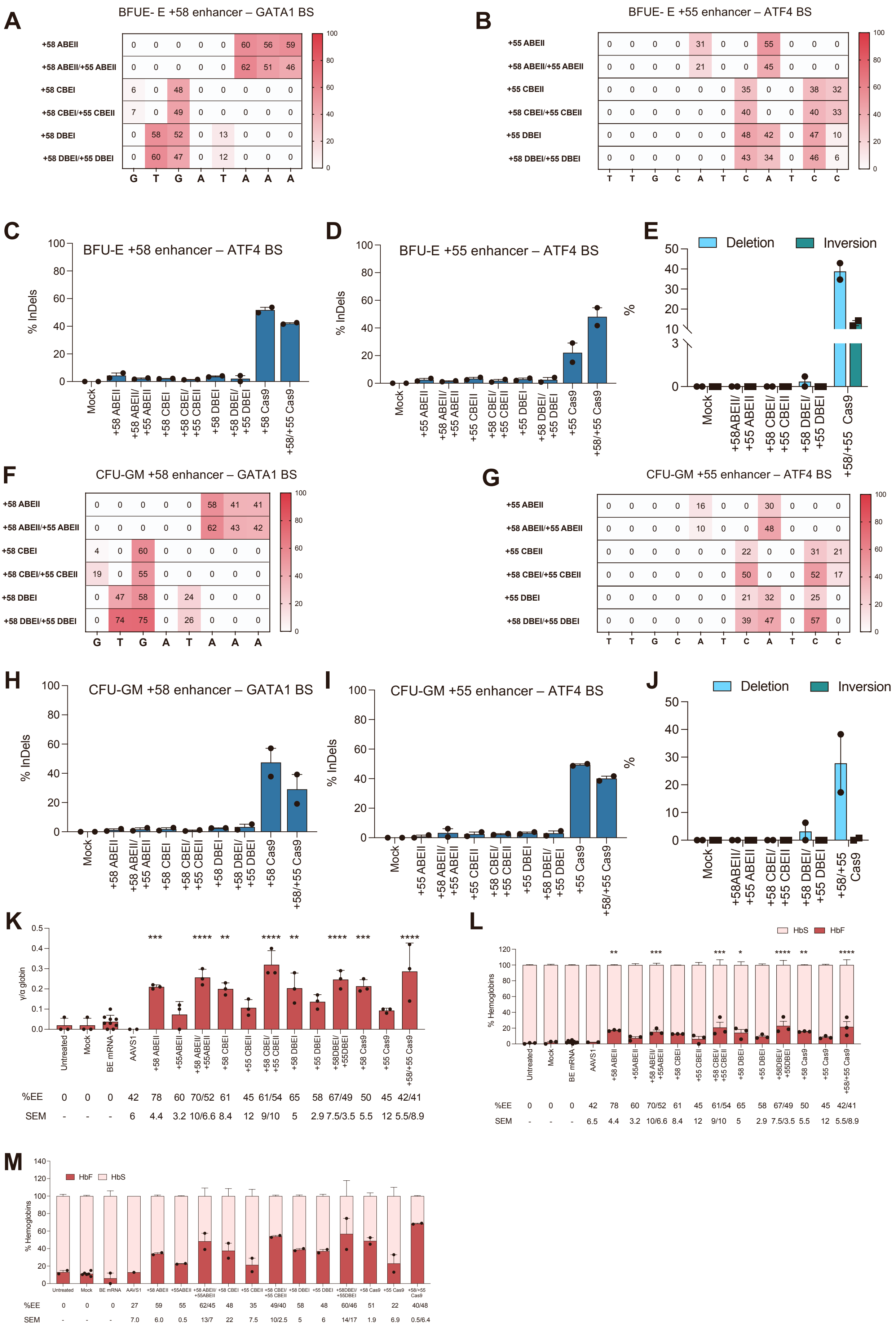
A
BFUE- E +58 enhancer – GATA1 BS
+58 ABEII
+58 ABEII/+55 ABEII
+58 CBEI
+58 CBEI/+55 CBEII
+58 DBEI
+58 DBEI/+55 DBEI
G T G A T A A A
B
BFUE- E +55 enhancer – ATF4 BS
+55 ABEII
+58 ABEII/+55 ABEII
+55 CBEII
+58 CBEI/+55 CBEII
+55 DBEI
+58 DBEI/+55 DBEI
T T G C A T C A T C C
C
BFU-E +58 enhancer – ATF4 BS
% InDels
D
BFU-E +55 enhancer – ATF4 BS
E
Deletion
Inversion
F
CFU-GM +58 enhancer – GATA1 BS
G
CFU-GM +55 enhancer – ATF4 BS
H
CFU-GM +58 enhancer – GATA1 BS
I
CFU-GM +55 enhancer – ATF4 BS
J
K
γ/α globin
%EE 0 0 0 42 78 60 70/52 61 45 61/54 65 58 67/49 50 45 42/41
SEM - - - 6 4.4 3.2 10/6.6 8.4 12 9/10 5 2.9 7.5/3.5 5.5 12 5.5/8.9
L
HbS
HbF
% Hemoglobins
%EE 0 0 0 42 78 60 70/52 61 45 61/54 65 58 67/49 50 45 42/41
SEM - - - 6.5 4.4 3.2 10/6.6 8.4 12 9/10 5 2.9 7.5/3.5 5.5 12 5.5/8.9
M
%EE 0 0 0 27 59 55 62/45 48 35 49/40 58 48 60/46 51 22 40/48
SEM - - - 7.0 6.0 0.5 13/7 22 7.5 10/2.5 5 6 14/17 1.9 6.9 0.5/6.4

Figure S4. Multiplex base editing of the erythroid-specific *BCL11A* enhancers.

A-B. C-G to T-A or/and A-T to G-C base-editing efficiency in the +58-kb (**A**) or +55-kb (**B**) regions calculated using EditR in samples subjected to Sanger sequencing in BFU-E colonies derived from SCD HSPCs edited at either the +58-kb or the +55-kb region, or simultaneously edited at both the +58-kb and the +55-kb regions. Data are expressed as mean (n = 2 biologically independent experiments, 2 donors).

C-D. Frequency of InDels in the +58-kb (**C**) or +55-kb (**D**) regions measured by TIDE analysis, in samples subjected to Sanger sequencing in pools of BFU-E colonies differentiated from SCD HSPCs edited at either the +58-kb or the +55-kb regions, or simultaneously edited at both the +58-kb and the +55-kb regions. Data are expressed as mean ± SEM (n = 2 biologically independent experiments, 2 donors).

E. Frequency of the 3.2-kb deletion/inversion, measured by ddPCR, in BFU-E colonies simultaneously edited at the +58-kb and +55-kb regions. Data are expressed as mean ± SEM (n = 2 biologically independent experiments, 2 donors).

F-G. C-G to T-A or/and A-T to G-C base-editing efficiency in the +58-kb (**F**) or +55-kb (**G**) regions calculated using EditR in samples subjected to Sanger sequencing in CFU-GM colonies derived from SCD HSPCs edited at either the +58-kb or the +55-kb region, or simultaneously edited at both the +58-kb and the +55-kb regions. Data are expressed as mean (n = 2 biologically independent experiments, 2 donors).

H-I. Frequency of InDels in the +58-kb (**H**) or +55-kb (**I**) regions measured by TIDE analysis, in samples subjected to Sanger sequencing in pools of CFU-GM colonies, differentiated from SCD HSPCs edited at either the +58-kb or the +55-kb regions, or simultaneously edited at both the +58-kb and the +55-kb regions. Data are expressed as mean ± SEM (n = 2 biologically independent experiments, 2 donors).

J. Frequency of the 3.2-kb deletion/inversion, measured by ddPCR, in CFU-GM colonies simultaneously edited at the +58-kb and +55-kb regions. Data are expressed as mean ± SEM (n = 2 biologically independent experiments, 2 donors). *$P \leq 0.05$; (two-way ANOVA with Sidak correction for multiple comparisons. Comparison of mock vs edited samples).

K. Expression of γ ($^{G}\gamma$ + $^{A}\gamma$)-globin chains measured by RP-HPLC in RBCs derived from SCD HSPCs. γ-globin expression was normalized to α-globin. The EE ± SEM is indicated for each sample in the lower part of the panel. Data are expressed as mean ± SEM (n=3 biologically independent experiments, 3 donors). **$P \leq 0.01$; ***$P \leq 0.001$; ****$P \leq 0.0001$ (one-way ANOVA with Dunnett correction for multiple comparisons. Statistical significance between mock and edited samples is depicted in the graph).

L. Analysis of HbF and HbS by cation-exchange HPLC in RBCs derived from edited SCD HSPCs. We calculated the percentage of each Hb type over the total Hb tetramers. The EE ± SEM is indicated for each sample in the lower part of the panel. Data are expressed as mean ± SEM (n = 3 biologically independent experiments, 3 donors). *$P \leq 0.05$; **$P \leq 0.01$; ***$P \leq 0.001$; ****$P \leq 0.0001$ (two-way ANOVA with Dunnett correction for multiple comparisons. Statistical significance between mock and edited samples is depicted in the graph).

M. Analysis of HbF and HbS by cation-exchange HPLC in pools of BFU-E colonies derived from edited SCD HSPCs. We calculated the percentage of each Hb type over the total Hb tetramers. Data are expressed as mean ± SEM (n = 2 biologically independent experiments, 2 donors).

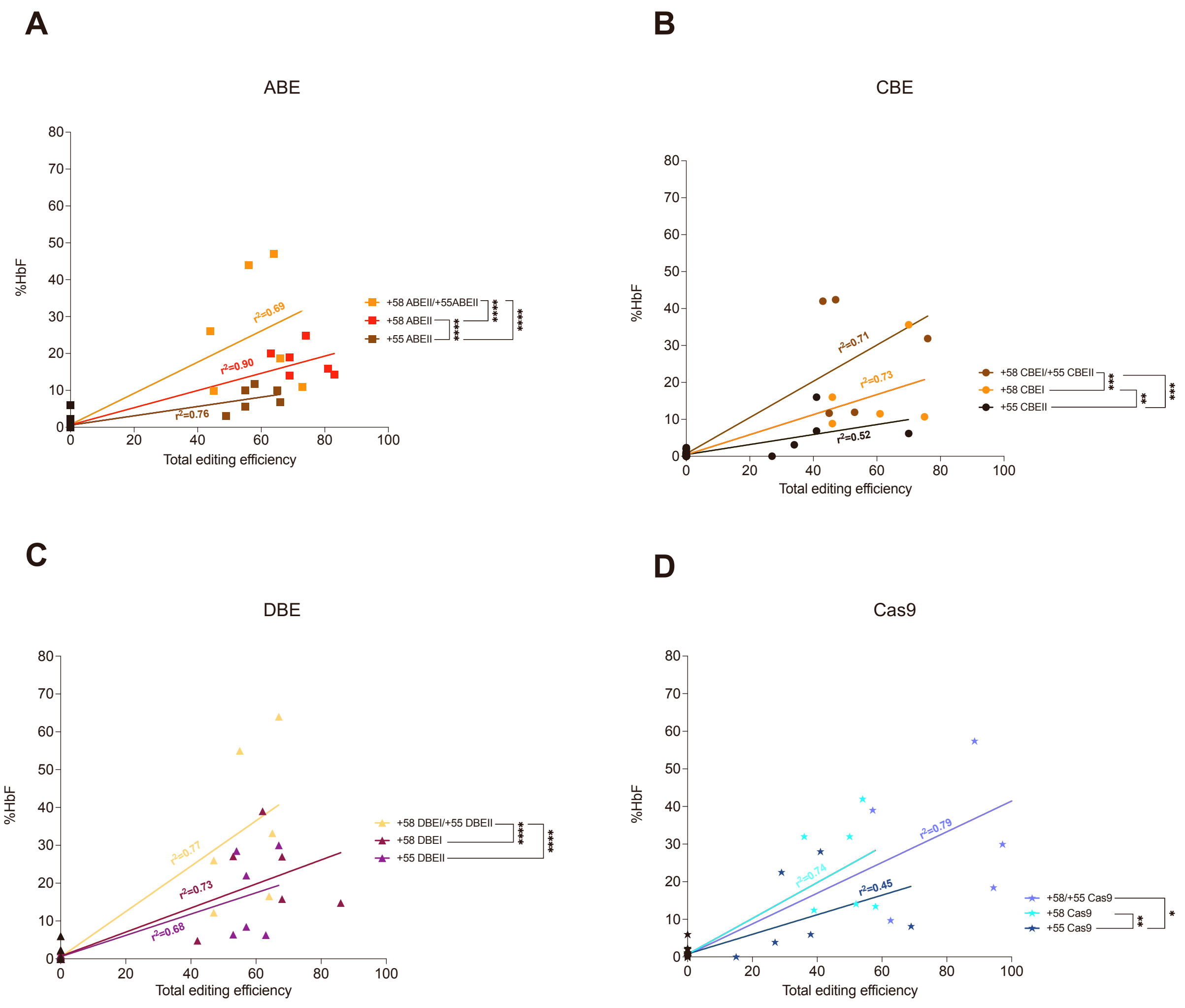

Figure S5. Correlation between HbF expression and editing efficiency obtained with ABE, CBE, DBE, and Cas9 nuclease.

Correlation between HbF expression and editing efficiency in erythroid cells derived from SCD HSPCs edited with either ABE (**A**), CBE (**B**), DBE (**C**), or Cas9-nuclease (**D**) (erythroblasts and pools of BFU-E; n = 3 biological independent experiments, 3 donors). HbF expression was measured by cation-exchange HPLC and calculated over the total Hb tetramers. Total editing efficiency was calculated by adding the base editing and Cas9 editing efficiency (determined by Sanger sequencing at the individual sites) to the frequency of the 3.2-kb deletion/inversion detected by ddPCR. Base-editing efficiency was calculated using EditR, and Cas9-editing efficiency (InDels) was calculated using TIDE in samples subjected to Sanger sequencing. *$P \leq 0.05$; **$P \leq 0.01$; ***$P \leq 0.001$; ****$P \leq 0.0001$ (Multiple t test).

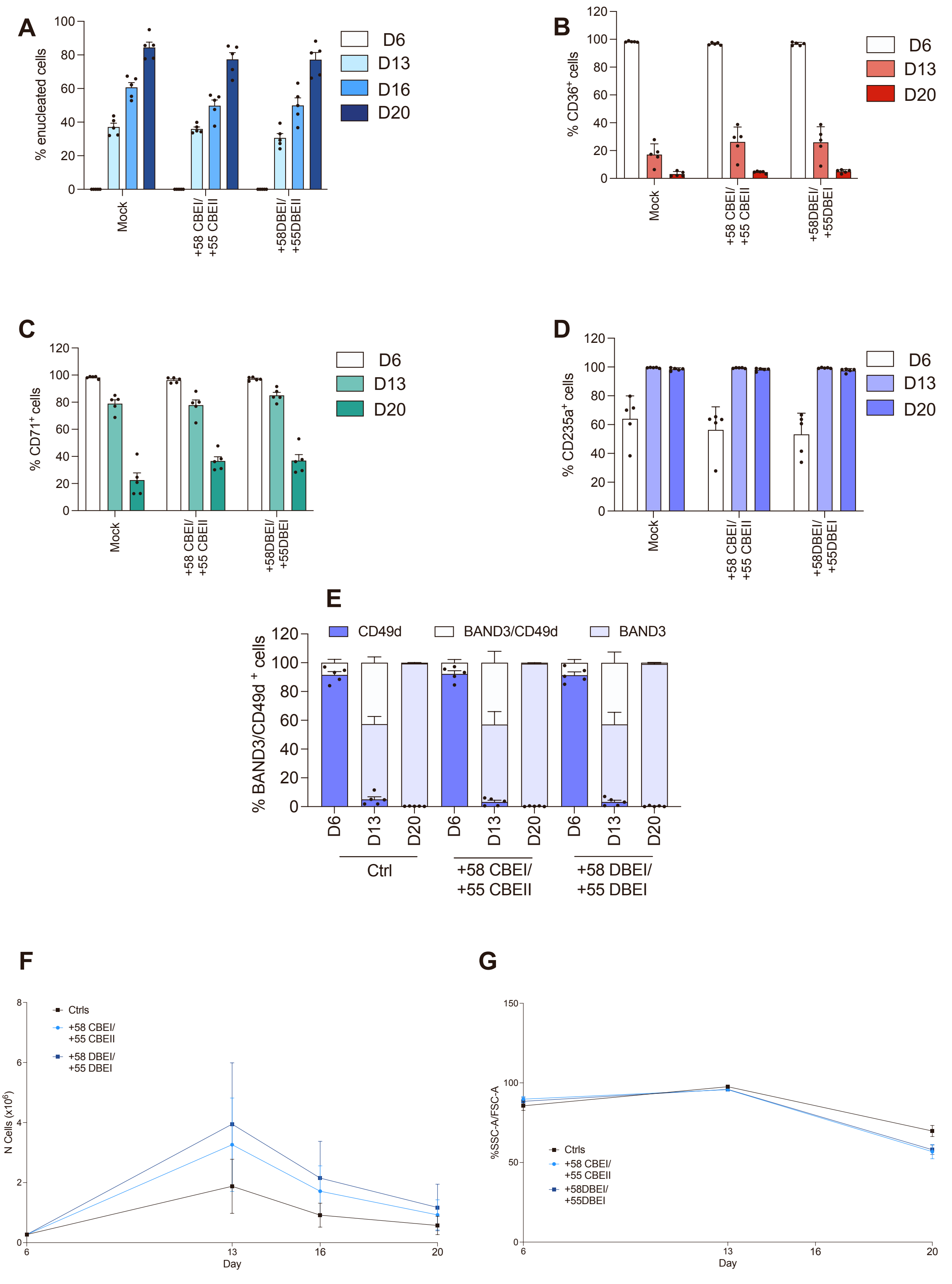

A
% enucleated cells
D6
D13
D16
D20
Mock
+58 CBEI/
+55 CBEII
+58DBEI/
+55DBEII
B
% CD36+ cells
D6
D13
D20
Mock
+58 CBEI/
+55 CBEII
+58DBEI/
+55DBEI
C
% CD71+ cells
D6
D13
D20
Mock
+58 CBEI/
+55 CBEII
+58DBEI/
+55DBEI
D
% CD235a+ cells
D6
D13
D20
Mock
+58 CBEI/
+55 CBEII
+58DBEI/
+55DBEI
E
% BAND3/CD49d + cells
CD49d
BAND3/CD49d
BAND3
D6
D13
D20
Ctrl
+58 CBEI/
+55 CBEII
+58 DBEI/
+55 DBEI
F
N Cells (x10^6)
Day
Ctrls
+58 CBEI/
+55 CBEII
+58 DBEI/
+55 DBEI
G
%SSC-A/FSC-A
Day
Ctrls
+58 CBEI/
+55 CBEII
+58DBEI/
+55DBEI

Figure S6. Erythroid differentiation of SCD HSPCs upon multiplex base editing of the +58-kb and +55-kb regions.

A. Frequency of enucleated cells at day 6, 13, 16, and 20 of erythroid differentiation, as measured by flow cytometry analysis of DRAQ5 nuclear staining in control and edited samples. Data are expressed as mean ± SEM (n = 1 biologically independent experiment, 5 donors).

B-D. Frequency of $CD36^+$ (**B**), $CD71^+$ (**C**), and $CD235a^+$ (**D**) cells at day 6, 13, and 20 of erythroid differentiation, as measured by flow cytometry analysis of CD36, CD71, and CD235a erythroid markers. Data are expressed as mean ± SEM (n = 1 biologically independent experiment, 5 donors).

E. Frequency of $CD49d^+$, $BAND3^+$ and $CD49d^+/BAND3^+$ in $7AAD^-/CD235a^+$ cells at day 6, 13, and 20 of erythroid differentiation, as measured by flow cytometry analysis of CD49d and BAND3 erythroid markers. Data are expressed as mean ± SEM (n = 1 biologically independent experiment, 5 donors).

F. Analysis of the cell growth over time. The number of cells was measured at multiple time points (day 6, 13, 16, and 20 of erythroid differentiation) to assess growth dynamics. Data are expressed as mean ± SEM (n = 1 biologically independent experiment, 5 donors).

G. Analysis of cell viability over time. We reported the percentage of live cells over the total live and dead/apoptotic cells identified based on size and granularity. Viability was assessed at days 6, 13, 16, and 20 of erythroid differentiation. Data are presented as mean ± SEM (n = 1 biologically independent experiment, from 5 donors).

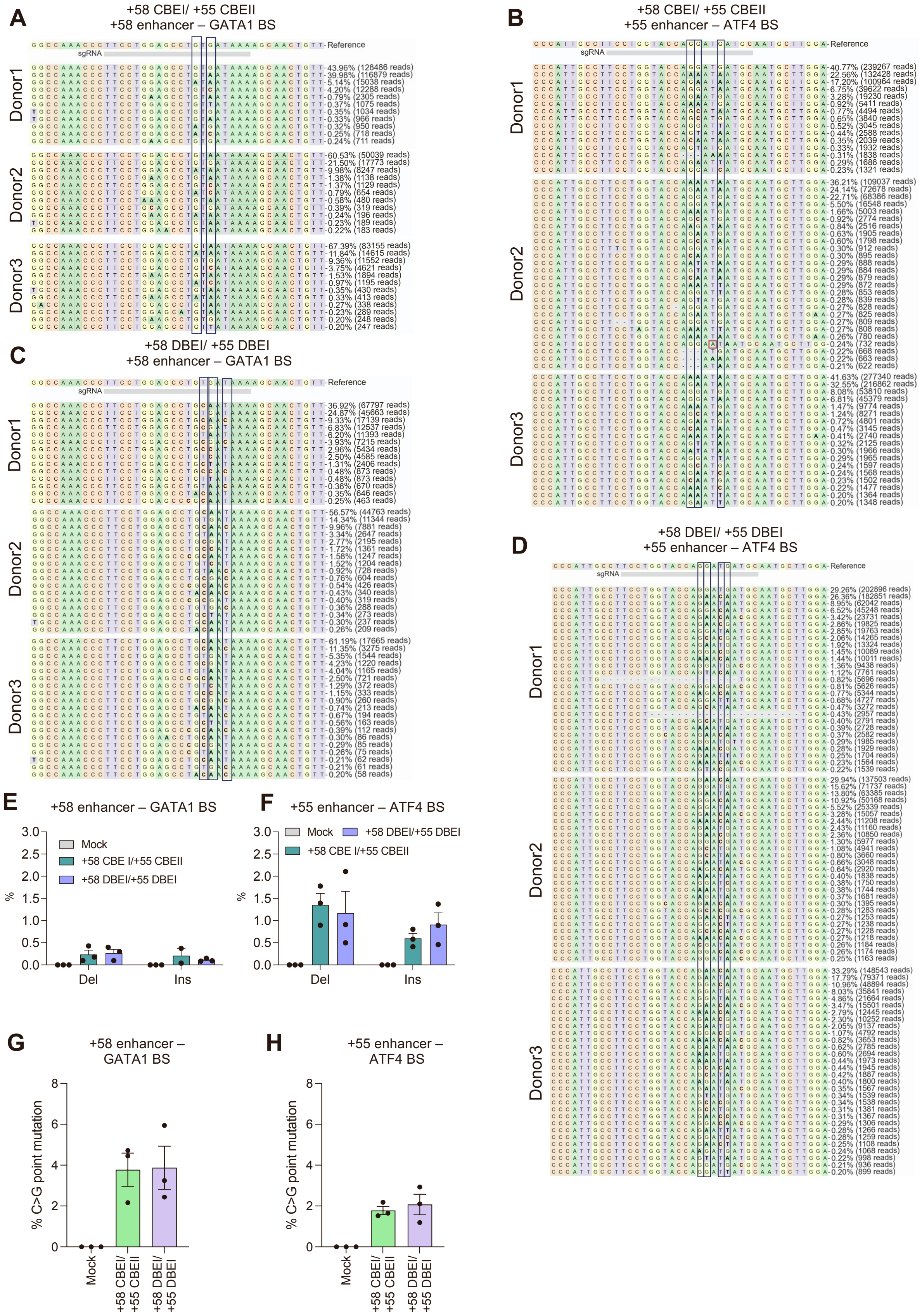
A
+58 CBEI/ +55 CBEII
+58 enhancer – GATA1 BS
Reference
sgRNA
Donor1
Donor2
Donor3
B
+58 CBEI/ +55 CBEII
+55 enhancer – ATF4 BS
C
+58 DBEI/ +55 DBEI
+58 enhancer – GATA1 BS
D
+58 DBEI/ +55 DBEI
+55 enhancer – ATF4 BS
E
+58 enhancer – GATA1 BS
Mock
+58 CBE I/+55 CBEII
+58 DBEI/+55 DBEI
%
Del
Ins
F
+55 enhancer – ATF4 BS
G
+58 enhancer – GATA1 BS
% C>G point mutation
Mock
+58 CBEI/ +55 CBEII
+58 DBEI/ +55 DBEI
H
+55 enhancer – ATF4 BS

Figure S7. NGS analysis of on-target editing in erythroid cells derived from SCD HSPCs upon multiplex base editing of the +58-kb and +55-kb regions.

A-D. Frequency and sequence of modified and unmodified alleles in edited SCD samples, for +58 CBEI/ +55 CBEII (**A** and **B**) and +58 DBEI/ +55 DBEI (**C** and **D**) profiles at the +58-kb (**A** and **C**) and +55-kb (**B** and **D**) regions, as measured by targeted NGS. Target base positions are highlighted with a blue box. The red box indicates inserted bases. Grey squares indicate deletions. (n = 3 biologically independent experiments, 3 donors).

E-F. Deletions and Insertions frequency in edited SCD samples for +58 CBEI/ +55 CBEII and +58 DBEI/ +55 DBEI profiles at the +58-kb (**E**) and +55-kb (**F**) regions, as measured by targeted NGS. Data are expressed as mean ± SEM (n = 3 biologically independent experiments, 3 donors).

G-H. C-G to G-C base-editing frequency in the +58-kb (**G**) or +55-kb (**H**) regions as measured by targeted NGS in SCD HSPCs simultaneously edited at the +58-kb and the +55-kb region. Data are expressed as mean ± SEM (n = 3 biologically independent experiments, 3 donors).

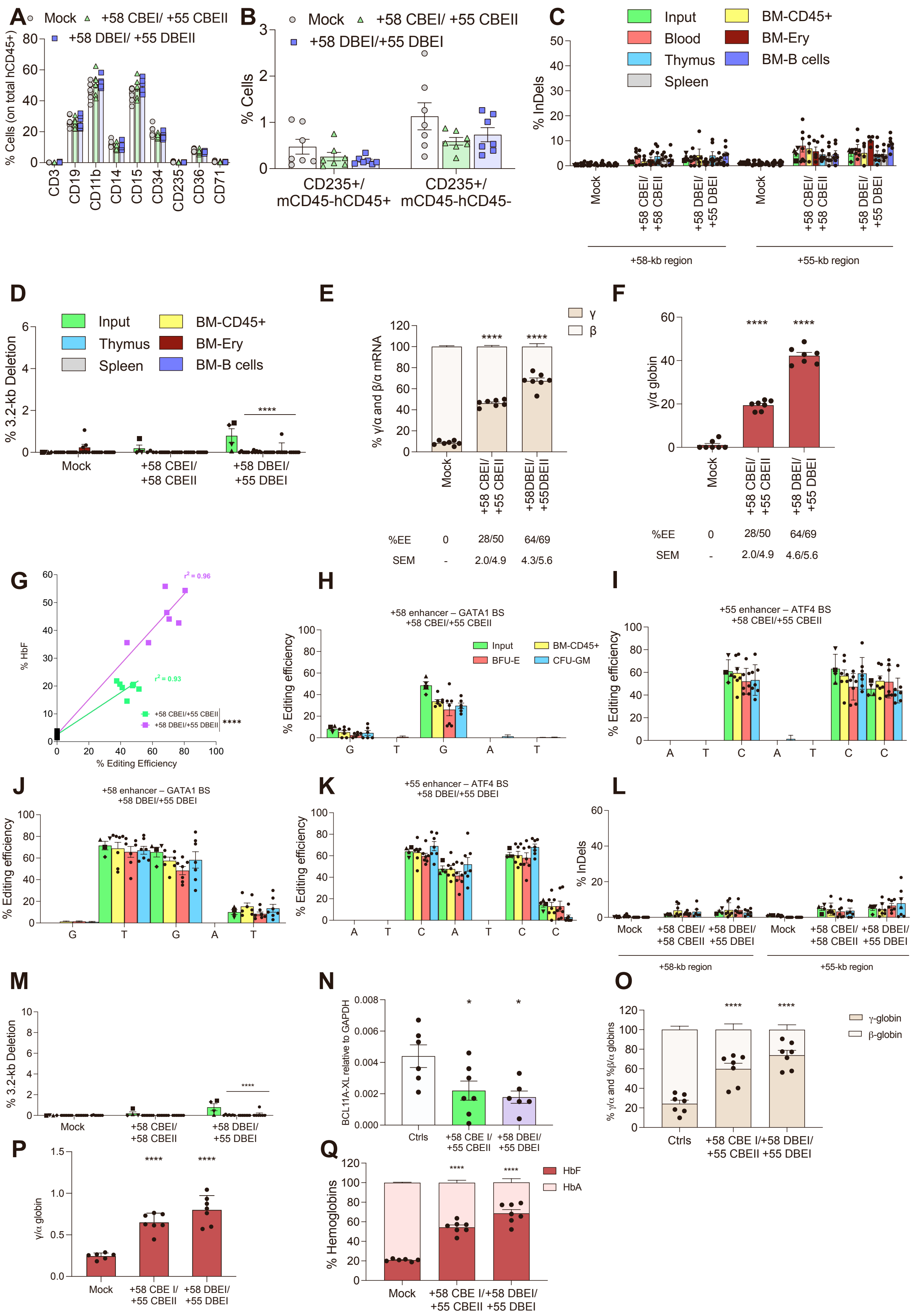
A
Mock
+58 CBEI/ +55 CBEII
+58 DBEI/ +55 DBEII
% Cells (on total hCD45+)
CD3
CD19
CD11b
CD14
CD15
CD34
CD235
CD36
CD71
B
Mock
+58 CBEI/ +55 CBEII
+58 DBEI/+55 DBEI
% Cells
CD235+/ mCD45-hCD45+
CD235+/ mCD45-hCD45-
C
Input
Blood
Thymus
Spleen
BM-CD45+
BM-Ery
BM-B cells
% InDels
Mock
+58 CBEI/ +58 CBEII
+58 DBEI/ +55 DBEI
+58-kb region
+55-kb region
D
Input
Thymus
Spleen
BM-CD45+
BM-Ery
BM-B cells
% 3.2-kb Deletion
Mock
+58 CBEI/ +58 CBEII
+58 DBEI/ +55 DBEI
****
E
γ
β
% γ/α and β/α mRNA
****
Mock
+58 CBEI/ +55 CBEII
+58DBEI/ +55DBEI
%EE 0 28/50 64/69
SEM - 2.0/4.9 4.3/5.6
F
γ/α globin
****
Mock
+58 CBEI/ +55 CBEII
+58 DBEI/ +55 DBEI
%EE 0 28/50 64/69
SEM - 2.0/4.9 4.6/5.6
G
% HbF
% Editing Efficiency
r² = 0.96
r² = 0.93
+58 CBEI/+55 CBEII
+58 DBEI/+55 DBEII
****
H
+58 enhancer – GATA1 BS
+58 CBEI/+55 CBEII
% Editing efficiency
Input
BFU-E
BM-CD45+
CFU-GM
G T G A T
I
+55 enhancer – ATF4 BS
+58 CBEI/+55 CBEII
% Editing efficiency
A T C A T C C
J
+58 enhancer – GATA1 BS
+58 DBEI/+55 DBEI
% Editing efficiency
G T G A T
K
+55 enhancer – ATF4 BS
+58 DBEI/+55 DBEI
% Editing efficiency
A T C A T C C
L
% InDels
Mock
+58 CBEI/ +58 CBEII
+58 DBEI/ +55 DBEI
+58-kb region
+55-kb region
M
% 3.2-kb Deletion
Mock
+58 CBEI/ +58 CBEII
+58 DBEI/ +55 DBEI
****
N
BCL11A-XL relative to GAPDH
*
Ctrls
+58 CBE I/ +55 CBEII
+58 DBEI/ +55 DBEI
O
γ-globin
β-globin
% γ/α and %β/α globins
****
Ctrls
+58 CBE I/ +55 CBEII
+58 DBEI/ +55 DBEI
P
γ/α globin
****
Mock
+58 CBE I/ +55 CBEII
+58 DBEI/ +55 DBEI
Q
HbF
HbA
% Hemoglobins
****
Mock
+58 CBE I/ +55 CBEII
+58 DBEI/ +55 DBEI

Figure S8. Simultaneous base editing of *BCL11A* enhancers in repopulating HSCs and erythroid and granulo-monocytic progenitors derived from engrafting HSPCs.

A. Frequency of human T (CD3) and B (CD19) lymphoid, myeloid (CD11b, CD14, and CD15), HSPC (CD34), and erythroid (CD235a, CD36 and CD71) cells in BM in mice transplanted with control and edited HSPCs, 16/17 weeks after the transplantation (n = 7 mice per condition). Each data point represents an individual mouse. Data are expressed as mean ± SEM. (two-way ANOVA; not significant).

B. Frequency of human CD235a cells in mCD45-hCD45+ and mCD45-hCD45- BM populations in mice transplanted with control and edited HSPCs, 16/17 weeks after transplantation (n = 7 mice per condition). Each data point represents an individual mouse. Data are expressed as mean ± SEM. (two-way ANOVA; not significant).

C. Frequency of InDels calculated by TIDE for the +58 CBEI/+55 CBEII and +58 DBEI/+55 DBEI profiles at the +58-kb and +55-kb regions in the input, blood-, BM-, thymus-, and spleen-derived human samples subjected to Sanger sequencing. BM samples include $CD45^+$ cells, B cells, and erythroid cells (Ery). Data are expressed as mean ± SEM [n = 1 biologically independent experiment (Input), n = 4 to 7 mice per group]. Each data point represents an individual mouse (two-way ANOVA; not significant). The frequency of Indels in the input was calculated in cells cultured in the HSPC medium (■), in liquid erythroid cultures (▲), and pools of BFU-E (◆) and CFU-GM (▼).

D. Frequency of the 3.2-kb deletion, measured by ddPCR, for samples simultaneously edited at the +58-kb and +55-kb regions for the input, BM-, thymus-, and spleen-derived human samples. BM samples include $CD45^+$ cells, B cells, and erythroid cells (Ery). Data are expressed as mean ± SEM [n = 1 biologically independent experiment (Input), n = 7 mice per group]. Each data point represents an individual mouse. ****$P \leq 0.0001$ between the input and the different human hematopoietic populations derived from engrafted HSCs (two-way ANOVA with Tukey's correction for multiple comparisons). The frequency of 3.2-kb deletion in the input was calculated in cells cultured in the HSPC medium (■), in liquid erythroid cultures (▲), and in pools of BFU-E (◆) and CFU-GM (▼).

E. RT-qPCR analysis of γ ($^G\gamma$ + $^A\gamma$)- and β-globin mRNA in BM sorted human $CD235a^+$ erythroid cells in mice transplanted with control and edited HSPCs, 16/17 weeks post-transplantation. γ- and β^S-globin mRNA expression was normalized to α-globin mRNA and expressed as a percentage of the γ- + β^S-globin mRNA. The EE ± SEM is indicated for each sample in the lower part of the panel. Each data point represents an individual mouse. Data are expressed as mean ± SEM (n = 7 mice per group). ****$P \leq 0.0001$ (two-way ANOVA with Dunnett correction for multiple comparisons. Comparison mock vs edited samples).

F. Expression of γ ($^G\gamma$ + $^A\gamma$)-globin chains measured by RP-HPLC in BM-sorted human $CD235a^+$ erythroid cells in mice transplanted with control and edited HSPCs, 16/17 weeks post-transplantation. γ-globin expression was normalized to α-globin. The EE ± SEM is indicated for each sample in the lower part of the panel. Each data point represents an individual mouse. Data are expressed as mean ± SEM (n = 7 mice per group). ****$P \leq 0.0001$ (one-way ANOVA with Dunnett correction for multiple comparisons. Comparison mock vs edited samples).

G. Correlation between HbF expression and editing efficiency in human $CD235a^+$ BM-sorted erythroid cells obtained from mice transplanted with control and edited HSPCs. HbF was measured by cation-exchange HPLC and calculated over the total Hb tetramers. Base-editing efficiency was calculated using EditR software. ****$P \leq 0.0001$ (Multiple t test).

H-K. C-G to T-A or/and A-T to G-C base-editing efficiency, calculated using EditR for the +58 CBEI/ +55 CBEII (**H** and **I**) and +58 DBEI/ +55 DBEI (**I** and **J**) profiles at the +58-kb (**G** and **I**) and +55-kb (**J** and **K**) regions in the input, BM $CD45^+$ cells, and pooled BFU-E and CFU-GM derived from BM $CD45^+$ cells and subjected to Sanger sequencing. Data are expressed as mean ± SEM [n = 1 biologically independent experiment (Input), n = 7 mice per group]. Each data point represents an individual mouse. The frequency of base editing in the input was calculated in cells cultured in the HSPC medium (■), in liquid erythroid cultures (▲), and in pools of BFU-E (◆) and CFU-GM (▼).

L. Frequency of InDels calculated by TIDE for the +58 CBEI/+55 CBEII and +58 DBEI/+55 DBEI profiles at the +58-kb and +55-kb regions in the input, BM $CD45^+$ cells, and pooled BFU-E and CFU-GM derived from BM $CD45^+$ cells and subjected to Sanger sequencing. Data are expressed as mean ± SEM [n = 1 biologically independent experiment (Input), n = 7 mice per group]. Each data point represents an individual mouse (two-way ANOVA; not significant). The frequency of Indels in the input was calculated in cells cultured in the HSPC medium (■), in liquid erythroid cultures (▲), and in pools of BFU-E (◆) and CFU-GM (▼).

M. Frequency of the 3.2-kb deletion, measured by ddPCR, for samples simultaneously edited at the +58-kb and +55-kb regions for the input, BM $CD45^+$ cells, and pooled BFU-E and CFU-GM derived from BM $CD45^+$ cells. Data are expressed as mean ± SEM [n = 1 biologically independent experiment (Input), n = 7 mice per group]. Each data point represents an individual mouse. ****$P \leq 0.0001$ between the

input and the different human hematopoietic populations derived from engrafted HSCs (two-way ANOVA with Tukey's correction for multiple comparisons). The frequency of 3.2-kb deletion in the input was calculated in cells cultured in the HSPC medium (■), in liquid erythroid cultures (▲), and in pools of BFU-E (◆) and CFU-GM (▼).

N. RT-qPCR analysis of *BCL11A-XL* expression in pooled BFU-E derived from BM $CD45^+$ cells. *BCL11A-XL* mRNA expression was normalized to *GAPDH*. Data are expressed as mean ± SEM (n = 7 mice per group). Each data point represents an individual mouse. *P ≤ 0.05 (One-way ANOVA with Dunnett's correction for multiple comparison. Comparison of mock vs edited samples).

O. RT-qPCR analysis of γ ($^G\gamma$ + $^A\gamma$)- and β-globin mRNA in pooled BFU-E derived from BM $CD45^+$ cells. γ- and β^-globin mRNA expression was normalized to α-globin mRNA and expressed as a percentage of the γ- + β- β-globin mRNA. Each data point represents an individual mouse. Data are expressed as mean ± SEM (n = 7 mice per group). ****$P \leq 0.0001$ (two-way ANOVA with Dunnett)

P. Expression of γ ($^G\gamma$ + $^A\gamma$)-globin chains measured by RP-HPLC in pooled BFU-E derived from BM $CD45^+$ cells. γ-globin expression was normalized to α-globin. Each data point represents an individual mouse. Data are expressed as mean ± SEM (n = 7 mice per group). ****$P \leq 0.0001$ (one-way ANOVA with Dunnett correction for multiple comparisons. Comparison mock vs edited samples).

Q. Analysis of HbF and HbS by CE-HPLC in pooled BFU-E derived from BM $CD45^+$ cells. We calculated the percentage of each Hb type over the total Hb tetramers. Data are expressed as mean ± SEM (n = 7 mice per group). Each data point represents an individual mouse. ****P ≤ 0.0001 (two-way ANOVA with Dunnett correction for multiple comparisons. Comparison of mock vs edited samples).

SUPPLEMENTARY TABLES

sgRNA	Sequence (5' to 3')	Mismatches	Position (hg38)	Strand	Score	Type
GATA_bs_1	TTCCTCCCAGGCTCCAGGAAAGG	3	Chr1:150511390-150511412	-	0.55	Exonic
	CCTATCACTGGCTCCAGGAAGGG	3	Chr4:4338932-4338954	-	0.6	Intergenic
	CATTCACAGGCTCCAGGAATGG	2	Chr12:125257599-125257620	-	0.93	Intronic
	GTTATCTCAGCCTCCAGGAATGG	3	Chr18:45013184-45013206	-	1.05	Intronic
	TTTCTCAGAGCCTCCAGGAAGGG	3	Chr10:57611578-57611600	-	1.14	Intergenic
	CTGATCACAGCTCCAGGAAAGG	2	Chr19:42519879-42519900	-	1.28	Intronic
	TTTGTCTCAGCTCCAGGAAAGG	2	Chr1:49994908-49994929	-	1.41	Intronic
	TTTGTCTCAGCTCCAGGAAAGG	2	Chr3:3404786-3404807	-	1.41	Intronic
	TTGATCTCAGGCACCAGGAACGG	3	Chr3:11574097-11574119	+	1.48	Intronic
	CTTATCATAGGCCCCAGGAAAGG	2	Chr17:73712637-73712660	-	1.49	Intergenic
ATF4_bs_2	TCTTCTTCCTGGTACCAGGAGGG	3	Chr1:184873483-184873505	-	0.48	Intergenic
	GTACCTTCCTGGTACCAGGAAGG	3	Chr7:148128963-148128985	+	0.51	Intronic
	GTGTCAACCTGGTACCAGGATGG	3	Chr7:71049683-71049705	-	0.51	Intergenic
	GCGTCTTCCGGTACCAGGAGGG	2	ChrX:154158066-154158087	-	1.37	Intronic
	ACTCATCCTTGTACCAGGAAGG	2	Chr2:211197131-211197152	+	1.48	Intergenic
	GAAACATCCTGTACCAGGAGGG	2	Chr9:33373626-33373647	+	1.51	Intergenic
	GATCTTCCTTGTACCAGGAAGG	2	Chr11:16986847-16986868	+	1.55	Intronic
	GCAGCTCCTTGTACCAGGAAGG	2	Chr15:99988930-99988951	+	1.59	Intronic
	GCATCAGGCTGGCACCAGGAAGG	3	Chr16:50373084-50373106	-	1.6	Intergenic
	GCATCATCTAGTTACCAGGAAGG	3	Chr19:54317646-54317668	+	1.65	Intronic
	GCAGCCTCCTGGTGCCAGGAAGG	3	Chr14:105646275-105646297	+	1.68	Intergenic

Supplementary table 1. Predicted off-target sites.
Excel table containing the list of the top 5 GUIDE-seq predicted off-targets and of the top 5 *in silico* predicted off-targets.
